# Evidence-Based Checklist to Delay Cardiac Arrest in Brain-Dead Potential Organ Donors: The DONORS Cluster Randomized Clinical Trial

**DOI:** 10.1001/jamanetworkopen.2023.46901

**Published:** 2023-12-14

**Authors:** Glauco A. Westphal, Caroline Cabral Robinson, Natalia Elis Giordani, Cassiano Teixeira, Adriane Isabel Rohden, Bruna dos Passos Gimenes, Cátia Moreira Guterres, Itiana Cardoso Madalena, Luiza Vitelo Andrighetto, Sabrina Souza da Silva, Daiana Barbosa da Silva, Daniel Sganzerla, Alexandre Biasi Cavalcanti, Cristiano Augusto Franke, Fernando Augusto Bozza, Flávia Ribeiro Machado, Joel de Andrade, Luciano Cesar Pontes Azevedo, Silvana Schneider, Bianca Rodrigues Orlando, Cintia Magalhães Carvalho Grion, Fernando Albuerne Bezerra, Fernando Roberto Roman, Francisco Olon Leite, Íris Lima Ferraz Siqueira, João Fernando Piccolo Oliveira, Lúcio Couto de Oliveira, Maria de Fátima Rodrigues Buarque de Melo, Patrícia Berg Gonçalves Pereira Leal, Pedro Carvalho Diniz, Rafael Barbarena Moraes, Daniela Ferreira Salomão Pontes, Josélio Emar Araújo Queiroz, Luciano Serpa Hammes, Maureen O. Meade, Regis Goulart Rosa, Maicon Falavigna

**Affiliations:** 1 Responsabilidade Social–Programa de Apoio ao Desenvolvimento Institucional do Sistema Único de Saúde (PROADI-SUS), Hospital Moinhos de Vento (HMV), Porto Alegre, Rio Grande do Sul, Brazil; 2 Central Estadual de Transplantes de Santa Catarina, Rua Esteves Júnior, Florianópolis, Santa Catarina, Brazil; 3 Centro Hospitalar Unimed Joinville and Hospital Municipal São José, Joinville, Santa Catarina, Brazil; 4 Postgraduate Programme in Epidemiology, Universidade Federal do Rio Grande do Sul (UFRGS), Porto Alegre, Rio Grande do Sul, Brazil; 5 Hospital de Clínicas de Porto Alegre (HCPA), Porto Alegre, Rio Grande do Sul, Brazil; 6 Universidade Federal de Ciências da Saúde de Porto Alegre (UFCSPA), Porto Alegre, Rio Grande do Sul, Brazil; 7 HCor Research Institute, São Paulo, São Paulo, Brazil; 8 Hospital de Pronto de Socorro (HPS), Porto Alegre, Rio Grande do Sul, Brazil; 9 National Institute of Infectious Disease Evandro Chagas, Fundação Oswaldo Cruz (FIOCRUZ), Rio de Janeiro, Rio de Janeiro, Brazil; 10 Instituto D’Or de Pesquisa e Ensino (IDOR), Rio de Janeiro, Rio de Janeiro, Brazil; 11 Disciplina de Anestesiologia, Dor e Medicina Intensiva, Universidade Federal de São Paulo (UNIFESP), São Paulo, São Paulo, Brazil; 12 Hospital Israelita Alber Einstein, Morumbi, São Paulo, São Paulo, Brazil; 13 Department of Statistics, UFRGS, Porto Alegre, Rio Grande do Sul, Brazil; 14 Hospital Universitário São Francisco de Paula, Pelotas, Rio Grande do Sul, Brazil; 15 Hospital Escola, Universidade Federal de Pelotas (UFPEL), Pelotas, Rio Grande do Sul, Brazil; 16 Hospital Universitário Regional do Norte do Paraná, Londrina, Paraná, Brazil; 17 Hospital Evangélico de Londrina, Londrina, Paraná, Brazil; 18 Hospital Regional Tarcísio de Vasconcelos Maia, Mossoró, Rio Grande do Norte, Brazil; 19 Hospital Bom Jesus, Toledo, Paraná, Brazil; 20 Hospital Regional Norte, Centro Universitário Inta (UNINTA), Sobral, Ceará, Brazil; 21 Hospital de Urgência e Emergência de Rio Branco (HUERB), Rio Branco, Acre, Brazil; 22 Hospital de Base, Faculdade de Medicina de São José do Rio Preto (FAMERP), São José do Rio Preto, São Paulo, Brazil; 23 Hospital Geral Cleriston Andrade, Feira de Santana, Bahia, Brazil; 24 Hospital da Restauração, Recife, Pernambuco, Brazil; 25 Associação Beneficente da Santa Casa de Campo Grande, Campo Grande, Mato Grosso do Sul, Brazil; 26 Hospital Universitário, Universidade Federal do Vale do São Francisco (HU/UNIVASF), Petrolina, Pernambuco, Brazil; 27 Intensive Care, HCPA, Porto Alegre, Rio Grande do Sul, Brazil; 28 General Coordination Office, National Transplant System, Brazilian Ministry of Health, Esplanada dos Ministérios, Bloco G, Edifício Sede, Brasília, Distrito Federal, Brazil; 29 Department of Medicine, Department of Health Research Methods, Evidence and Impact, McMaster University, Hamilton, Ontario, Canada; 30 National Institute for Health Technology Assessment, UFRGS, Porto Alegre, Rio Grande do Sul, Brazil; 31 Department of Health Research Methods, Evidence, and Impact (HEI), McMaster University, Hamilton, Ontario, Canada

## Abstract

**Importance:**

The effectiveness of goal-directed care to reduce loss of brain-dead potential donors to cardiac arrest is unclear.

**Objective:**

To evaluate the effectiveness of an evidence-based, goal-directed checklist in the clinical management of brain-dead potential donors in the intensive care unit (ICU).

**Design, Setting, and Participants:**

The Donation Network to Optimize Organ Recovery Study (DONORS) was an open-label, parallel-group cluster randomized clinical trial in Brazil. Enrollment and follow-up were conducted from June 20, 2017, to November 30, 2019. Hospital ICUs that reported 10 or more brain deaths in the previous 2 years were included. Consecutive brain-dead potential donors in the ICU aged 14 to 90 years with a condition consistent with brain death after the first clinical examination were enrolled. Participants were randomized to either the intervention group or the control group. The intention-to-treat data analysis was conducted from June 15 to August 30, 2020.

**Interventions:**

Hospital staff in the intervention group were instructed to administer to brain-dead potential donors in the intervention group an evidence-based checklist with 13 clinical goals and 14 corresponding actions to guide care, every 6 hours, from study enrollment to organ retrieval. The control group provided or received usual care.

**Main Outcomes and Measures:**

The primary outcome was loss of brain-dead potential donors to cardiac arrest at the individual level. A prespecified sensitivity analysis assessed the effect of adherence to the checklist in the intervention group.

**Results:**

Among the 1771 brain-dead potential donors screened in 63 hospitals, 1535 were included. These patients included 673 males (59.2%) and had a median (IQR) age of 51 (36.3-62.0) years. The main cause of brain injury was stroke (877 [57.1%]), followed by trauma (485 [31.6%]). Of the 63 hospitals, 31 (49.2%) were assigned to the intervention group (743 [48.4%] brain-dead potential donors) and 32 (50.8%) to the control group (792 [51.6%] brain-dead potential donors). Seventy potential donors (9.4%) at intervention hospitals and 117 (14.8%) at control hospitals met the primary outcome (risk ratio [RR], 0.70; 95% CI, 0.46-1.08; *P* = .11). The primary outcome rate was lower in those with adherence higher than 79.0% than in the control group (5.3% vs 14.8%; RR, 0.41; 95% CI, 0.22-0.78; *P* = .006).

**Conclusions and Relevance:**

This cluster randomized clinical trial was inconclusive in determining whether the overall use of an evidence-based, goal-directed checklist reduced brain-dead potential donor loss to cardiac arrest. The findings suggest that use of such a checklist has limited effectiveness without adherence to the actions recommended in this checklist.

**Trial Registration:**

ClinicalTrials.gov Identifier: NCT03179020

## Key Points

**Question:**

Compared with usual care, is an evidence-based checklist to guide clinical management effective in reducing cardiac arrest among brain-dead potential donors?

**Findings:**

In this cluster randomized trial of 1535 brain-dead potential donors from 63 hospitals, donor losses to cardiac arrest were not significantly lower in the intervention group than in the control group (9.4% vs 14.8%). In the intervention group with high adherence to the checklist, loss through cardiac arrest was significantly lower compared with the control group (5.3% vs 14.8%).

**Meaning:**

Findings of this study suggest that use of such a checklist has limited effectiveness without adherence to the actions recommended in this checklist.

## Teaser

This cluster randomized clinical trial evaluates the use of a clinical management checklist and incidence of cardiac arrest in brain-dead potential organ donors in Brazil.

## Introduction

Missed opportunities for organ transplant from neurologically deceased donors is a global concern.^1^ Even within hospitals with support for organ donation, there are numerous factors at play, including delays in identifying potential organ donors, delays in referring brain-dead potential donors to organ procurement organizations, and variation in skills of health professionals in discussing organ donation with family members. Furthermore, suboptimal and unstandardized management of brain-dead potential donors is one of the reasons for low quality of donated organs or even loss of brain-dead potential donors to cardiac arrest.^2,3,4^ Loss to cardiac arrest varies from 3.1% to 10.0%^5,6,7^ among eligible brain-dead potential donors (defined by the World Health Organization as “medically suitable persons who have been declared dead on the basis of neurological criteria, as stipulated by law”^1^^[pS31]^). This rate reaches above 20.0%^4,8,9,10,11^ among potential organ donors (defined by the World Health Organization as persons “whose clinical condition is suspected to fulfill brain death criteria”^1^^[pS31]^).

Goal-directed checklists have been found to improve the quality of care and consequently the conversion of brain-dead potential donors to actual organ donors,^8,9,11^ the number of organs transplanted per donor,^12,13,14,15^ and posttransplant graft function.^16,17^ Given the observational nature of these findings and the variability of their protocols for deceased donor care, clinicians and researchers have called for randomized trials to confirm the results.^16,18^ The Donation Network to Optimize Organ Recovery Study (DONORS) was a cluster randomized trial designed to evaluate the effectiveness of an evidence-based, goal-directed checklist in the clinical management of brain-dead potential donors in the intensive care unit (ICU).

## Methods

DONORS was an open-label, parallel-group, cluster randomized clinical trial conducted from June 20, 2017, to November 30, 2019, at 63 hospital ICUs across Brazil. We followed the prespecified study protocol^19^ and statistical analysis plan (Supplement 1)^20^ as well as the logic model depicted in eFigure 1 in Supplement 2. The institutional review boards of the participating hospitals approved the study and waived the informed consent requirement according to national Brazilian laws.^21^ We followed the Consolidated Standards of Reporting Trials (CONSORT) reporting guideline for cluster randomized trials.^22^

### Study Sites and Participants

**Figure 1.**
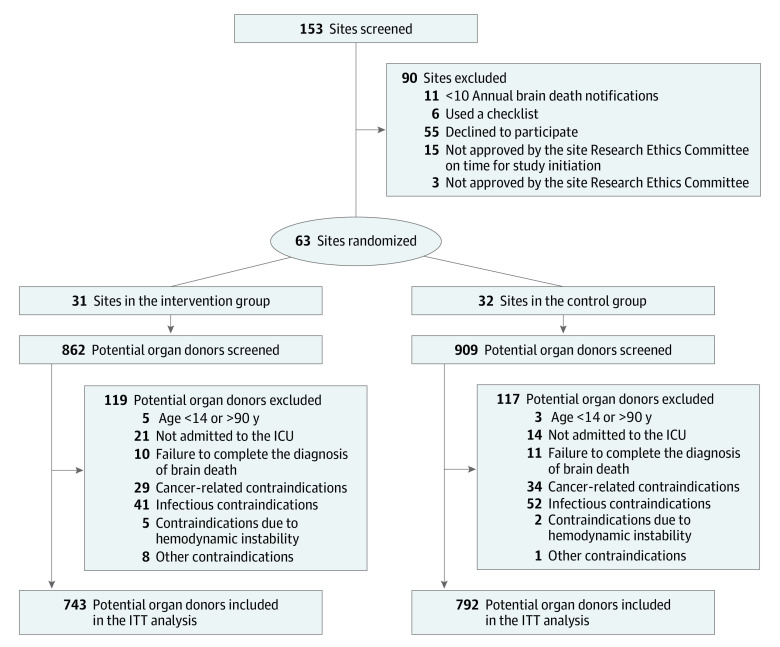
Patient Flowchart ICU indicates intensive care unit; ITT, intention to treat.

At cluster level, eligible hospitals were those with an annual mean number of 10 or more brain-dead potential donors over the previous 2 years. We excluded hospitals with any clinical decision-making tools already in place. The concealed randomization of hospitals used variable block sizes and stratification by the previous median annual number of reported brain deaths (eg, >29 vs ≤29). Each cluster was a single hospital. Sites were randomized to the intervention group (provided checklist guidance) or control group (provided usual care) (Figure 1).

We enrolled patients in the ICU aged 14 to 90 years who had a condition consistent with brain death after the first clinical examination.^1,23^ According to Brazilian regulations, brain death is determined after 2 clinical examinations by 2 independent physicians at an interval of at least 1 hour, 1 apnea test, and 1 ancillary test.^23^ We excluded individuals who were not suitable for organ donation according to the criteria of Brazil’s National Transplant System (eTable 1 in Supplement 2).^24^ Potential organ donors were randomized to the intervention group (received checklist guidance) or control group (received usual care) (Figure 1).

### Study Interventions

**Figure 2.**
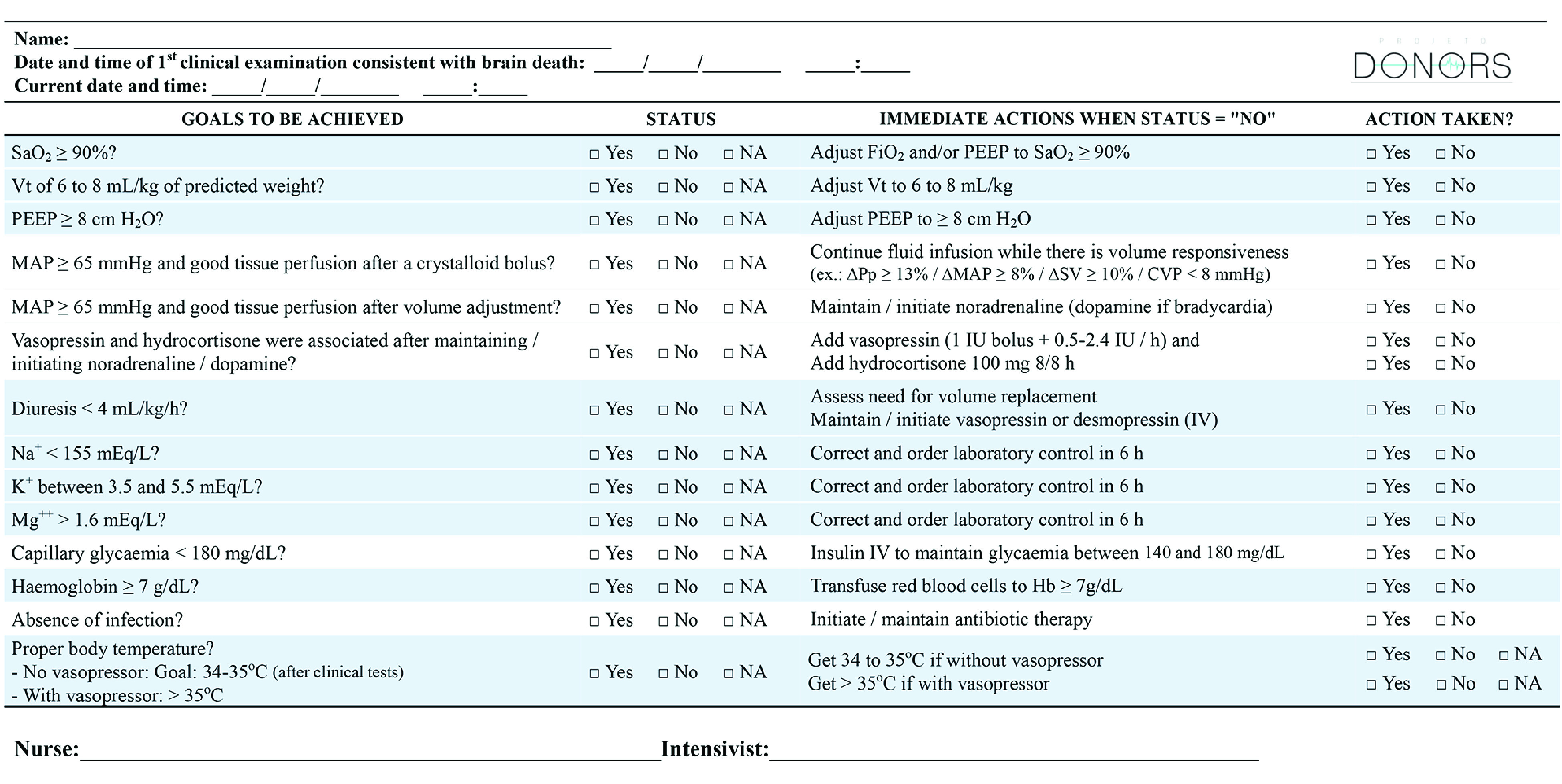
Bedside Goal-Directed Checklist to Guide the Care of Brain-Dead Potential Donors ΔPp indicates pulse pressure respiratory variation; ΔSV, stroke volume respiratory variation; CVP, central venous pressure; FiO_2_, fraction of inspired oxygen; H_2_O, water; Hb, hemoglobin; IV, intravenous; K^+^, potassium; MAP, mean arterial pressure; Mg^++^, magnesium; Na^+^, sodium; PEEP, positive end-expiratory pressure; SaO_2_, arterial oxygen saturation; and Vt, tidal volume.

For patients in the intervention group, a goal-directed checklist to guide brain-dead potential donor care (Figure 2) was administered bedside.^19^ The DONORS investigators convened a task force to develop an evidence-based clinical practice guideline for the management of brain-dead potential donors,^25^ using the GRADE (Grading of Recommendations, Assessment, Development, and Evaluations) method^26^ in accordance with standards of the Guidelines International Network and the US Institute of Medicine^27^ (eMethods 1 in Supplement 2). The recommendations from the guideline served as the basis for the evidence-based checklist tested in this trial.

The checklist was designed to maximize the conversion of potential to actual organ donors by preventing the loss of brain-dead potential donors to cardiac arrest and to increase organ viability. It consists of 13 goals and 14 actions, including mechanical ventilation, vasoactive support, hormonal supplementation, electrolyte control, body temperature control, and administration of antibiotics and blood products (Figure 2).

Expert clinicians visited all sites randomized to the intervention group to provide standardized 4-hour training sessions to the ICU and in-hospital donation and transplant coordination staff. The content of these sessions included world and national scenarios of organ donation, recommendations from the evidence-based clinical practice guideline, and the composition and administration of the paper-based bedside checklist.^19^

Based on studies that analyzed the effect of meeting physiological goals on referral after 6 hours^11^ and after 12 to 18 hours of the donor referral, as well as before procurement,^12,13,14,16^ the checklist was administered every 6 hours, while the patient was considered a brain-dead potential donor. Given the nature of the intervention, both investigators and health care staff were aware of the study group assignment.

For patients in the control group, usual care was provided. We instructed the ICU and in-hospital donation and transplant coordination staff at control sites to maintain routine care without sharing information about the checklist.

For both study groups, a family interview was conducted. The staff were trained, through an online course and a face-to-face course, to prepare for this task according to the standardized Spanish model of family interview regarding organ transplant (eTable 2 in Supplement 2).^28,29^

### Data Collection and Monitoring

The ICU and in-hospital donation and transplant coordination staff recorded data (eg, inclusion and exclusion criteria, baseline clinical variables, brain death diagnosis variables, clinical management variables, and family interview information) from both study groups at the time of enrollment; at 6, 12, and 24 hours after enrollment; and every 24 hours for 14 days or until transfer from the ICU to the operating room. The same staff transferred data from case report forms to an electronic data capture system (REDCap; Vanderbilt University). Central study personnel produced monthly reports of data completeness and accuracy for all sites based on site visits and central statistical data monitoring to assess data quality and data sources.^19^

### Study Outcomes

The primary outcome was loss of brain-dead potential donors to cardiac arrest. Secondary outcomes were the conversion of brain-dead potential donors to actual organ donors (defined by the initiation of organ retrieval surgery^1^) and the number of solid organs recovered per actual organ donor. Exploratory outcomes were 12 clinical goals related to the brain-dead potential donor management (eMethods 2 in Supplement 2).

### Statistical Analysis

All analyses followed a published statistical analysis plan,^20^ unless otherwise specified. An independent statistics committee that was unaware of group assignments accessed the research database and reviewed all analyses for clarity, suitability, and accuracy.

We determined that a sample of 60 clusters and 1140 brain-dead potential donors (19 per site) would provide at least 80% power to detect an absolute reduction of 10% in brain-dead potential donor loss to cardiac arrest (from 28% in the control group to 18% in the intervention group, based on pilot study findings^11^), with an intracluster correlation coefficient of 0.05^20^ and a 2-sided α level of 5%. We limited the enrollment at each hospital to 30 patients.

The main analysis for each outcome was performed at the individual level. All participants were analyzed with an intention-to-treat approach, according to their assigned study group at the cluster level, regardless of the extent of adherence to protocol. Although a survival analysis that was adjusted for cluster effect (frailty model) was planned according to the statistical analysis plan, after an extensive discussion we realized that a method to properly account for competing risks was needed. Therefore, we assessed the treatment effect on the outcomes with a generalized estimating equation model that considered Poisson distribution and an exchangeable working correlation matrix.^20^ Intracluster correlation coefficients were estimated using the same method.

The prespecified subgroup analyses for the primary outcome considered age (>60 vs ≤60 years), cause of brain death (traumatic vs nontraumatic), and patient illness severity on ICU admission (>median vs ≤ median Simplified Acute Physiology Score [SAPS] 3).^20^ A set of prespecified sensitivity analyses evaluated whether checklist adherence higher than the median adherence in the intervention group was associated with the primary outcome.^20^ Adherence to each action was considered complete if the recommended course of action was performed or if no action was needed according to the checklist. For each brain-dead potential donor, we identified the proportion of all checklist recommendations with adherence (eFigure 2 in Supplement 2) and the median checklist adherence across donors in the intervention group. Next, we reproduced the generalized estimating equation model to stratify data from the intervention group according to whether their checklist adherence was greater than vs less than or equal to the prespecified median adherence.^20^ Post hoc analyses explored the relationship between checklist adherence and the rate of brain-dead potential donor loss to cardiac arrest. We extended the sensitivity analysis of checklist adherence among donors to quintiles of adherence, assessing for a dose-response relationship. Reasoning that adherence may have more to do with sites than patients, we assessed for a modifying effect of high vs low levels of adherence at the hospital level.

Prespecified exploratory analyses were conducted at the individual level.^20^ To each exploratory outcome (eMethods 2 in Supplement 2), we considered data collected from all the time points along the clinical management. Post hoc analyses besides the sensitivity analyses and the reasons for conducting them are described in eTable 3 in Supplement 2.

A sensitivity analysis was conducted for the primary outcome of the study according to occurrence of potential failures in the screening and inclusion of consecutive patients, estimated number of brain death notifications in the ICU, and donation rate for each site before the study.

*P* < .05 indicated statistical significance. We did not impute outcome data except in the context of the sensitivity analysis for the primary outcome.^20^ We conducted all analyses from June 15 to August 30, 2020, using the survival and geepack packages in R, version 3.5.2 (R Development Core Team).

## Results

Figure 1 shows hospital selection and patient flow throughout the study. Among the 63 hospitals across Brazil (eFigure 3 in Supplement 2), 31 (49.2%) were assigned to the intervention group and 32 (50.8%) to the control group. The median (IQR) numbers of hospital beds and ICU beds were 265 (195-635) and 45 (29-69), respectively. Almost all ICU types were mixed (62 [98.5%]), and most of the hospitals were emergency (57 [90.5%]) and/or neurological (48 [76.2%]) referral centers. Twenty-five hospitals (39.7%) were transplant centers, which was the only characteristic that differed between groups. The median (IQR) number of annual brain death notifications was 23 (16-36) (Table 1).

**Table 1.** Characteristics of the Study Sites and Patients at Baseline

Characteristic	Study group, No. (%)	
	Intervention	Control
**Sites**		
No./total No. of hospitals	31/63 (49.2)	32/63 (50.8)
No. of hospital beds, median (IQR)	282.5 (193.2-607.0)	248.5 (198.5-469.2)
No. of ICU beds, median (IQR)	50.0 (29.5-68.5)	39.0 (28.0-68.0)
No. of ICU beds or hospital beds, median (IQR)	13.2 (10.7-17.9)	14.1 (12.1-18.1)
No. of adult ICU beds, median (IQR)	34.0 (24.0-50.5)	26.0 (20.0-47.2)
ICU type		
Surgical	1 (3.2)	0
Mixed	30 (96.7)	32 (100)
Hospital type		
Public	17 (54.8)	17 (53.0)
Private	14 (45.2)	15 (47.0)
Emergency referral center	28 (90.3)	29 (91.0)
Neurological referral center	24 (77.4)	24 (75.0)
Teaching activity	23 (74.2)	26 (81.0)
Transplant center	10 (32.2)	15 (47.0)
No. of annual brain death notifications, median (IQR)^a^^,^^b^	24.0 (16.2-34.5)	22.0 (16.1-37.2)
**Patients**		
No./total No. of brain-dead potential donors	743/1535 (48.4)	792/1535 (51.6)
Age		
Median (IQR), y	50.8 (35.8-61.2)	51.5 (36.8-62.9)
>60 y	203 (27.3)	242 (30.6)
≤60 y	440 (72.7)	550 (69.4)
Sex		
Female	312 (42.0)	314 (39.6)
Male	431 (58.0)	242 (60.4)
SOFA score at enrollment, median (IQR)	11.1 (9.0-13.2)	10.0 (9.4-12.1)
SAPS 3 score at ICU admission, median (IQR)	73.5 (64.2-80.3)	72.0 (63.0-80.0)
Comorbidities		
Diabetes	96 (12.9)	89 (11.2)
Hypertension	311 (41.9)	314 (39.6)
Kidney failure requiring dialysis	16 (2.2)	20 (2.5)
Chronic respiratory disease^c^	14 (1.9)	11 (1.4)
Heart failure	17 (2.3)	21 (2.7)
Chronic liver disease^d^	2 (0.3)	2 (0.3)
Cause of brain injury		
Trauma	245 (33.0)	240 (30.3)
Stroke	409 (55.0)	468 (59.1)
Anoxia	56 (7.5)	52 (6.6)
Other^e^	33 (4.4)	32 (4.0)
Use of antimicrobial medication^a^	467 (62.9)	500 (63.1)
LOS before brain-death diagnosis, median (IQR), d	4.3 (1.8-8.5)	4.1 (1.9-7.9)

Abbreviations: ICU, intensive care unit; LOS, length of stay; SAPS, Simplified Acute Physiology Score; SOFA, Sequential Organ Failure Assessment.

^a^ Identified at the time of the first clinical examination.

^b^ Number of annual brain death notifications considered the percentage of brain-dead potential donors clinically managed in the ICU.

^c^ Chronic respiratory disease was defined as restrictive, obstructive, or vascular disease severe enough to limit performance of the activities of daily living or chronic hypoxia, hypercapnia, polycythemia, pulmonary hypertension, or ventilator dependence.

^d^ Chronic liver disease was defined as biopsy-proven cirrhosis or proven portal hypertension or previous history of hepatic insufficiency, encephalopathy, or coma.

^e^ Other causes included subarachnoid hemorrhage (aneurysm or venous artery malformation), brain tumor, exogenous intoxication, and meningitis.

Of the 1771 brain-dead potential donors screened, 1535 were included (intervention group: 743 [48.4%]; control group: 792 [51.6%]). These patients included 626 females (40.8%) and 673 males (59.2%), with a median (IQR) age of 51 (36.3-62.0) years. The main cause of brain injury was stroke (877 [57.1%]), followed by trauma (485 [31.6%]) (Table 1).

### Primary and Secondary Outcomes

**Figure 3.**
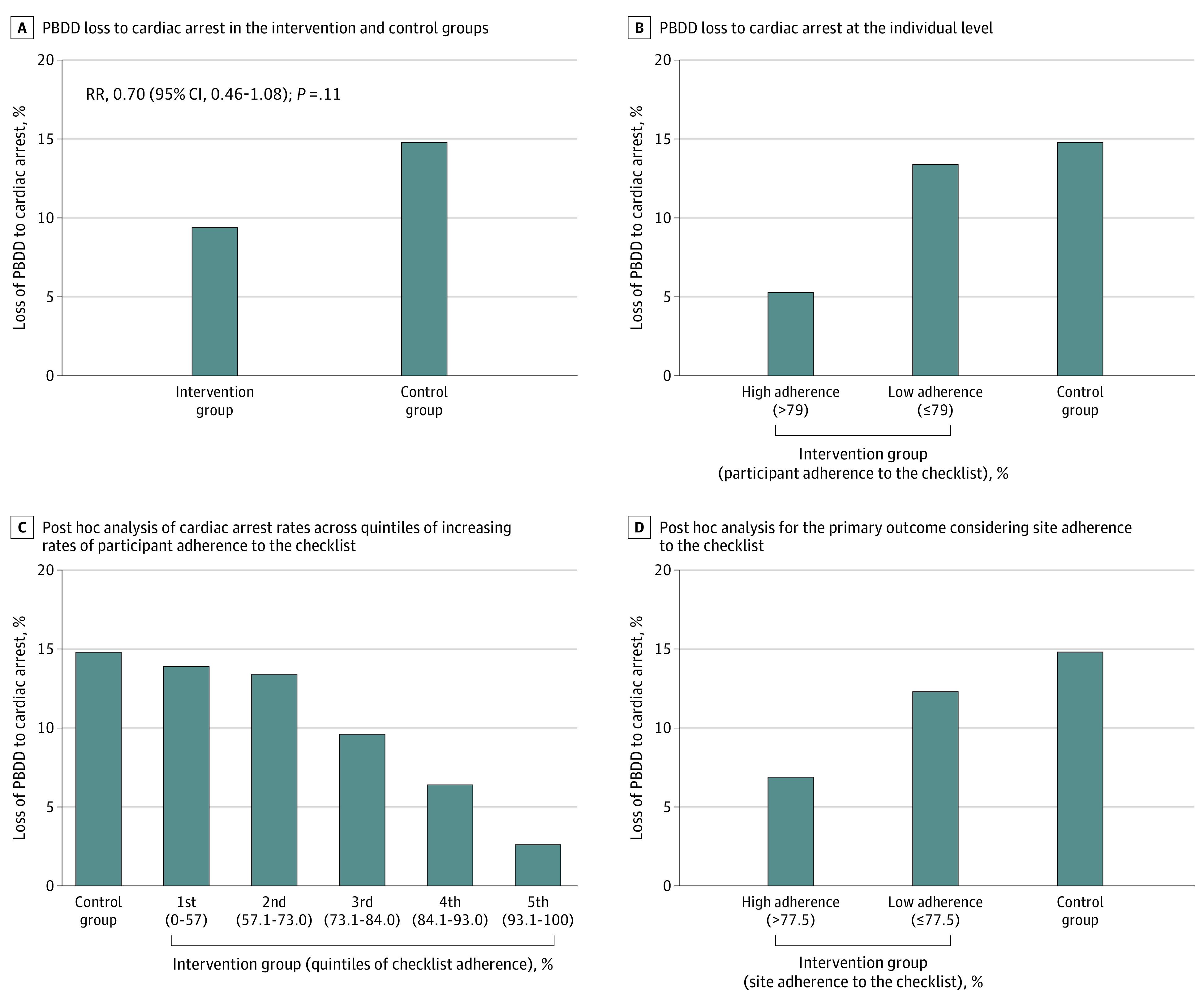
Brain-Dead Potential Donors Lost Due to Cardiac Arrest in the Study Groups and by Adherence to the Intervention RR indicates risk ratio.

One hundred eighty-seven brain-dead potential donors were lost to cardiac arrest, of whom 70 (9.4%) were in the intervention group and 117 (14.8%) were in the control group (risk ratio [RR], 0.70; 95% CI, 0.46-1.08; *P* = .11; number needed to treat [NNT] = 18.5) (Figure 3A). Although the point estimate decrease in brain-dead potential donor losses to cardiac arrest was 5.2% (relative risk difference, 36.5%), the absence of significance was maintained after adjusting for time to event (eTable 4 in Supplement 2). The proportion of actual organ donors was also similar between the intervention and control groups (RR, 1.04; 95% CI, 0.87-1.26; *P* = .65), as was the number of solid organs recovered per donor (mean difference, 0.05; 95% CI, −0.15 to 0.25; *P* = .63) (Table 2).

**Table 2.** Primary and Secondary Outcomes

Outcome	Overall (N = 1535)	Intervention group (n = 743)	Control group (n = 792)	Effect estimate (95% CI)^a^	*P* value
Primary					
Potential organ donors lost due to cardiac arrest, No. (%)^b^	187 (12.2)	70 (9.4)	117 (14.8)	RR: 0.70 (0.46 to 1.08)	.11
Secondary					
Actual organ donors, No. (%)	653 (42.5)	327 (44.0)	326 (41.2)	RR: 1.04 (0.87 to 1.26); RD: 1.80 (−6.01 to 9.62)	.65
Organs recovered per actual organ donor, mean (SD)	2.8 (1.07)	2.8 (1.11)	2.8 (1.04)	MD: 0.05 (−0.15 to 0.25)	.63

Abbreviations: RR, risk ratio, RD, risk difference, MD, mean difference.

^a^ Effect estimates were adjusted for cluster effect.

^b^ Intracluster correlation coefficient, 0.06 (95% CI, 0.02-0.10).

### Subgroup and Sensitivity Analyses

Subgroup analyses found no effect modifiers for the outcomes (eFigure 4 in Supplement 2). Sensitivity analyses at the individual level indicated the degree of adherence to the checklist as a modifier for the primary outcome (Figure 3) but not for the secondary outcomes (eTables 5 and 6 in Supplement 2). The median (IQR) checklist adherence per participant was 79.0% (64%-90%), and the characteristics of the participants with adherence greater than 79.0% were similar to those with adherence of 79.0% or less (eTable 7 in Supplement 2). Among participants with checklist adherence greater than 79%, brain-dead potential donor loss to cardiac arrest was lower than loss for the control group (5.3% vs 14.8%; RR, 0.41; 95% CI, 0.22-0.78; *P* = .006; NNT = 12.5). Among participants with checklist adherence of 79.0% or less, brain-dead potential donor loss to cardiac arrest was comparable with loss for the control group (13.4% vs 14.8%; RR, 0.93; 95% CI, 0.61-1.42; *P* = .75). The comparison between high and low adherence to the checklist showed fewer cardiac arrest in the higher-adherence subgroup (RR, 0.42; 95% CI, 0.25-0.73; *P* = .002; NNT = 10.5) (Figure 3B). In a post hoc analysis, we observed the following cardiac arrest rates across quintiles of increasing rates of donor checklist adherence: 14% (first quintile: 0%-57.0% adherence); 13% (second quintile: 57.1%-73.0% adherence); 9.6% (third quintile: 73.1%-84.0% adherence); 6.4% (fourth quintile: 84.1%-93.0% adherence); and 2.6% (fifth quintile: 93.1%-100% adherence) (Figure 3C).

For checklist adherence at the cluster level, hospitals were quite balanced (eTable 8 in Supplement 2). High-adherence hospitals (>77.5%) presented a lower rate of brain-dead potential donor losses to cardiac arrest than the control group (6.9% vs 14.8%; RR, 0.52; 95% CI, 0.29-0.95; *P* = .03) as well as a higher rate of effective donors (eTable 9 in Supplement 2). In contrast, in hospitals with checklist adherence of 77.5% or less, brain-dead potential donor losses to cardiac arrest were comparable with losses in the control group (12.3% vs 14.8%; RR, 0.90; 95% CI, 0.56-1.42; *P* = .64). In a direct comparison of hospitals with high vs low checklist adherence, brain-dead potential donor losses to cardiac arrest were lower (6.9% vs 12.3%; RR, 0.57; 95% CI, 0.32-0.99; *P* = .04; NNT = 18.5) (Figure 3D) and actual organ donors were higher (eTable 9 in Supplement 2) at high-adherence sites. The combination of high patient and site adherence potentiated the effect of the intervention on the primary outcome (eTable 10 in Supplement 2), but many low-adherence sites presented a good concentration of participants with high adherence (eFigure 5 in Supplement 2).

### Exploratory Analyses

An imbalance in physiological goals at baseline was detected between control and intervention groups, as between the high- and low-adherence clusters (eTable 11 in Supplement 2).

Considering repeated measures within subject adjusted for site, the intervention group exhibited higher global adherence with vasopressin (45.3% vs 23.6%; RR, 1.82; 95% CI, 1.53-2.17; *P* < .001), adequate circulatory parameters (52.8% vs 44.7%; RR, 1.20; 95% CI, 1.04-1.38; *P* = .003), and sodium level less than 155 mEq/L (to convert to millimoles per liter, multiply by 1.0) (66.2% vs 56.6%; RR, 1.15; 95% CI, 1.02-1.29; *P* < .001) (eTable 12 in Supplement 2). The adherence to the goals in the intervention group was substantially higher over time when the goal had already been met at baseline (eTable 13 in Supplement 2).

## Discussion

In DONORS, use of an evidence-based, goal-directed checklist did not result in significant reduction in brain-dead potential donor losses to cardiac arrest. Goal-directed checklists can serve as tools to promote adherence to the existing evidence-based clinical interventions, which could translate into better quality of care and improved outcomes.^30,31,32^ The checklist seemed to have contributed to greater adherence to essential goals: adequate circulatory parameters and serum sodium level less than 155 mEq/L. These findings could be explained by higher adherence to vasopressin use in the intervention group, an important factor in both hemodynamic and diabetes insipidus control. However, we were unable to demonstrate the effect of the checklist on brain-dead potential donor loss to cardiac arrest.

There might be alternative explanations for the lack of a statistically significant effect of the checklist on brain-dead potential donor loss to cardiac arrests. First, although DONORS, to our knowledge, was the largest randomized clinical trial of donor management ever conducted, it may have been underpowered to detect a clinically relevant effect size. The point estimate decrease in brain-dead potential donor loss to cardiac arrest was 5.2% (relative risk difference, 36.5%). In previous sample size calculations, a 10% decrease in cardiac arrests among brain-dead potential donors was estimated based on the pilot study.^11^ However, the cardiac arrest rate in the present study (14.8%) was lower than the rate in the pilot study (27.1%).^11^ It is possible that an overall improvement in the management of brain-dead potential donors over time, regardless of the study context, leads to lower baseline rates of cardiac arrests. Furthermore, the quality of the hospitals apparently did not influence the main outcome (eTables 14 and 15 in Supplement 2); thus, as previously reported,^33,34^ we cannot disregard that the mere participation in research activity may improve the control group outcomes.

The prespecified subgroup analysis was consistent with the main analysis, demonstrating that the effect of the intervention was the same, regardless of age, cause of brain death, or SAPS 3 scores. A sensitivity analysis showed that higher adherence to the checklist was associated with decreased cardiac arrest in brain-dead potential donors, which may have been influenced by a higher proportion of ICU beds and by the expertise acquired from previous brain-dead potential donor notifications in the most adherent sites (eTable 16 in Supplement 2). Among the less-adherent sites, many were able to promote good adherence in a large portion of participants, suggesting that the specific pattern of these sites is subject to modification. Nevertheless, these analyses are at risk for confounding bias and should be interpreted cautiously.

Despite the balance in demographic characteristics between the 2 groups, there was an imbalance in meeting physiological goals at baseline. This finding may be expected in open-label cluster studies as an effect of the implementation, indicating that eligible intervention sites may have instituted clinical measures even before they were formally included.

### Strengths and Limitations

Strengths of this study include the development of a clinical practice guideline^25^ using state-of-the-art methods to support the evidence-based checklist. The study generated a wide spectrum of sociodemographic scenarios, representing a clinical context with reproducible interventions. Additionally, it applied a standardized approach to the early recognition and enrollment of patients. It followed the recommended analytical approaches and reporting standards for cluster randomized clinical trials. The statistical analysis plan was published in advance, and the analyses were adjudicated by an independent statistical board.

This study also has several limitations. First, some of the checklist goals (lung-protective ventilation and glycemic control) have a low likelihood of preventing cardiac arrest. Second, the use of a checklist to improve donor management is only one of the many factors that affect clinical outcomes. Third, the relatively high rate of cardiac arrests stemmed from the early inclusion of patients in their course from brain injury to organ donation, after the first formal clinical examination that ascertained brain death.^1^ This factor may limit comparability with other countries that account for rates of cardiac arrest in brain-dead potential donors occurring only after consented donation. Fourth, allograft function in transplant recipients was not assessed. Fifth, lack of blinding may have introduced risk of bias due to modifications in health care associated with knowledge of group assignment. Sixth, we did not collect data on race and ethnicity; therefore, we cannot assess whether the results were associated with race and ethnicity. Seventh, limiting the inclusion criteria to hospitals with 10 or more referrals of brain-dead potential donors per year might limit the generalizability of the findings for lower-volume hospitals.

## Conclusions

This cluster randomized clinical trial was inconclusive in determining whether guiding clinical management by using an evidence-based, goal-directed checklist for donor care can reduce the loss of potential organ donors to cardiac arrest. Providing a checklist, per se, appeared to have limited effectiveness if appropriate measures were not taken to enhance the adherence to the recommended actions.

## Notes

Supplement 1.
Trial Protocol and Statistical Analysis Plan

Supplement 2. **eTable 1.** Exclusion Criteria for Brain-Dead Potential Organ Donors and Number of Exclusion Criteria Identified in Each Study Arm **eFigure 1.** Logic Model for Study Development **eFigure 2.** Description of the Adherence Measurement **eMethods 1.** Elaboration of the Evidence-Based Clinical Practice Guideline for the Management of Potential Brain-Dead Donors **eTable 2.** Family Interview Support Guide Based on the Spanish Model of Communication in Critical Situations **eMethods 2.** Exploratory Outcomes According to the Statistical Analysis Plan **eTable 3.** Post Hoc Analyses and Reasons for Their Selection **eFigure 3.** Geographic Distribution of the Included Sites **eTable 4.** Post Hoc Analysis Considering Adherence to the Intervention According to the Time of Brain-Dead Potential Donor Inclusion **eFigure 4.** Subgroup Analysis of the Primary Outcome (Loss of Brain-Dead Potential Organ Donors Due to Cardiac Arrest) **eTable 5.** Sensitivity Analysis for the Primary Outcome **eTable 6.** Sensitivity Analysis of Secondary Outcomes Regarding Adherence Considering Potential Organ Donor Adherence to the Intervention **eTable 7.** Characteristics of the Brain-Dead Potential Organ Donors at Baseline Considering Adherence to the Intervention at the Potential Organ Donor Level **eTable 8.** Characteristics of the Sites and the Brain-Dead Potential Organ Donors at Baseline Considering Site Adherence to the Intervention at the Site Level **eTable 9.** Post Hoc Analysis of Secondary Outcomes Regarding Adherence Considering Site Adherence to the Intervention **eTable 10.** Post Hoc Analysis for the Primary Outcome Combining Site and Brain-Dead Potential Organ Donor Adherence to the Intervention **eTable 11.** Comparison of Physiologic and Treatment Goals at Baseline Between the Intervention and Control Groups and Between High (>77.5%) and Low Adherence (≤77.5%) Centers **eTable 12.** Comparison of Goal Achievement Between the Intervention and Control Groups **eTable 13.** Individual Adherence to the Goals Over Time in the Intervention Group Comparing Participants Whose Physiology Met Parameters at the Baseline to Those Who Did Not Meet Parameters **eFigure 5.** Correlation Between Adherence to the Intervention per Site and at Individual Level (Potential Donor Adherence) **eTable 14.** Descriptive Analysis for a Proxy for Site Quality **eTable 15.** Post Hoc Analysis by Proxy for Site Quality Using a Directed Acyclic Graph (DAG) **eTable 16.** Post Hoc Analysis Adjusted for the Characteristics of Sites and Brain-Dead Potential Organ Donors Considering Site Adherence to the Intervention and Using a Directed Acyclic Graph (DAG)

Supplement 3. **Nonauthor Collaborators.** DONORS Investigators and BRICNet

Supplement 4.
Data Sharing Statement
