# Supplementary material for: Evidence-Based Checklist to Delay Cardiac Arrest in Brain-Dead Potential Organ Donors: The DONORS Cluster Randomized Clinical Trial
Source: JAMA Netw Open. 2023 Dec 14;6(12):e2346901. doi: 10.1001/jamanetworkopen.2023.46901 (PMC10722341; doi:10.1001/jamanetworkopen.2023.46901)
Supplement: Supplement 1. — Trial Protocol and Statistical Analysis Plan [file jamanetwopen-e2346901-s001.pdf]

Overall statement

In order to comply with transparency, we present here the final version approved by the Research Ethics Committee (REC) of the coordinator center and by the REC of all the 63 participating sites. In our context REC is similar to the Institutional Review Board, although is focused on regulatory and ethics issues. The statistical analysis plan (SAP) was not included in the protocol. Some statistical methods were updated and published* after REC approval however, before database lock, and are presented in the published SAP. The updating had no impact on protocol conduction or ethical aspects. To avoid misunderstanding we included a statement in the sessions containing updated information as was presented in the published SAP. This protocol is less detailed than the published protocol**, but is consistent with the clinicaltrials.gov record (NCT03179020).

*Giordani NE, Robinson CC, Westphal GA, et al. Statistical analysis plan for a cluster-randomised trial assessing the effectiveness of implementation of a bedside evidence-based checklist for clinical management of brain-dead potential organ donors in intensive care units: DONORS (Donation Network to Optimise Organ Recovery Study). *Trials.* 2020;21(1):540.

**Westphal GA, Robinson CC, Biasi A, et al. DONORS (Donation Network to Optimise Organ Recovery Study): Study protocol to evaluate the implementation of an evidence-based checklist for brain-dead potential organ donor management in intensive care units, a cluster randomised trial. BMJ Open. 2019;9(6):e028570.

ASSOCIAÇÃO HOSPITALAR MOINHOS DE VENTO

ADULT INTENSIVE CARE CENTER

Implementation of a clinical protocol for maintenance of potential donors in the intensive care unit: a cluster-randomized clinical trial

DONORS - ICU Trial: Donation (National) Network to Optimize Organ Recovery Study

Research Project

Version 3

Porto Alegre, 2018

TITLE

Implementation of a clinical protocol for maintenance of potential donors in the intensive care unit: a cluster-randomized clinical trial (DONORS - ICU Trial: Donation (National) Network to Optimize Organ Recovery Study)

PRINCIPAL INVESTIGATOR

Cassiano Teixeira Adult Intensive Care Unit, Hospital Moinhos de Vento, Porto Alegre, RS.

RESEARCH TEAM AT HOSPITAL MOINHOS DE VENTO:

Glauco Adrieno Westphal. Intensivist.

Regis Goulart Rosa. Intensivist.

Maicon Falavigna. Epidemiologist.

Caroline Cabral Robinson. Physical Therapist.

Cátia Moreira Guterres. Pharmacist.

Itiana Cardoso Madalena. Biologist.

Natalia Elis Giordani. Statistician.

Adriane Isabel Rohden. Pharmacist.

Sabrina Souza da Silva. Pharmacist.

Luiza Vitelo Andrighetto. Dietitian.

SPONSOR: Associação Hospitalar Moinhos de Vento (AHMV).

EXECUTOR: Associação Hospitalar Moinhos de Vento (AHMV).

DATE: December 20, 2018.

VERSION 3

Version	Amendment
**1 to 2**	Version 1 was revised to adjust text format and bureaucratic issues and to agree with the clinicaltrials.gov record, as follow:<br>1) Title page: Version was updated from 1 to 2.<br>2) Title page: Affiliation with the “Instituto de Educação e Pesquisa do Hospital Moinhos de Vento” was removed (internal institutional rearrangement).<br>3) Title page: Coordinator center staff was updated.<br>4) Summary: updated.<br>5) An Abbreviations list was inserted.<br>6) Abstract: the number of estimated participating hospitals was corrected; the eligibility criteria for hospitals were included; the keywords were corrected.<br>7) 1 INTRODUCTION: the rationale was included as a separate sub-session in the end of the introduction.<br>8) 1.2 Hypothesis: “guided by a checklist of goals” was added to the hypothesis’s description.<br>9) 2.2. Secondary objectives: “guided by a checklist of goals” was added to the objective’s description. A third secondary objective was included: “decrease the overall number of cardiac arrests”.<br>10) 3.2 Participants: Cluster definition: it was clarified that each cluster consist of the group of intensive care units in the same hospital, willing to participate in the study.<br>11) 3.3 Cluster eligibility criteria: The reference for potential donors records would be the two years previous the selection process instead of only

	2014.<br>12) 3.4 Randomization of the participating hospitals: Randomization parameters were corrected and expected number of randomized hospitals was changed from 30 to 35.<br>13) 3.5.1 Intervention: All the information regarding the intervention was assembled in this session. Any information regarding the checklist items was removed.<br>14) 3.6 Co-intervention: The information regarding the face-to face training for communication with the family was included.<br>15) 3.7.2 Secondary outcomes: a third secondary outcome was included "Number of overall cardiac arrests in the potential organ donors."<br>16) 3.7.3 Exploratory outcomes related with to care processes: the session was included to describe that exploratory outcomes were related with the process of care.<br>17) 3.8 DATA COLLECTION: The session "3.8.1 Parameters of interest" regarding the data that will be collected was inserted. Eligibility criteria for potential donors was included as sub-session "3.8.2". A sub-session for data monitoring description was included: 3.8.3 Monitoring of collected data.<br>18) 3.9.1 Sample size: the expected number of included hospitals were updated to 70.<br>19) 3.9.2 Statistical analysis: The information regarding statistical software was corrected.<br>20) 3.10 ETHICAL ASPECTS: All the information regarding ethical aspects was assembled in this session as well as the reasons for waiver of

	individual consent.<br>21) 5. ORGANIZATIONAL ASPECTS OF THE STUDY: this session was included to state the role of the coordinator center and partner organizations.<br>22) SCHEDULE and BUDGET were presented as separated documents from the study protocol.
**2 to 3**	The Steering Committee decided to remove the secondary outcome “Number of overall cardiac arrests in the potential organ donors”. The decision was based on the empirical observation that cardiac resuscitation of potential donors is not a regular practice in most the ICUs. Therefore, this result would bring information similar to the primary outcome.<br>The description regarding the minimum number of clusters participating in the study and the sample size was corrected from 70 to 60 in the Abstract and in the “3.9.1 Sample size” session.

SUMMARY

Abbreviations 8
ABSTRACT 9
1 INTRODUCTION 10
1.1 Rationale 11
1.2 Hypothesis 13
2 OBJECTIVES 14
2.1 Primary objective 14
2.2. Secondary objectives 14
3 METHODS 15
3.1 Study design 15
3.2 Participants 15
3.4 Randomization of participating facilities 16
3.5 Study arms 16
3.6 Co-intervention 17
3.7 Endpoints 17
3.8 Data collection 18
3.9 Sample and statistical analysis 21
3.10 Ethical aspects 23
4 EXPECTED RESULTS 26
5. ORGANIZATIONAL ASPECTS OF THE STUDY 27
6 REFERENCES 28

Abbreviations

ABTO – Brazilian Organ Transplantation Association (Associação Brasileira de Transplantes de Órgãos)

AMIB – Brazilian Association of Intensive Care Medicine (Associação de Medicina Intensiva Brasileira)

ANVISA – National Health Surveillance Agency

BD – brain death

BRICNet – Brazilian Research in Intensive Care Network

CGSNT – General Coordination of the Brazilian National Transplant System (Coordenação Geral do Sistema Nacional de Transplantes)

CGSNT – General Coordination of the Brazilian National Transplant System (Coordenação Geral do Sistema Nacional de Transplantes)

CNS – Brazilian National Health Council (Conselho Nacional de Saúde)

HIV – human immunodeficiency virus

ICF – Informed Consent Form

ICU – Intensive Care Unit

ID – identifier

IHTC – Intrahospital transplant coordination

MAP – mean arterial pressure

ONT – National Transplant Organization of Spain

PEEP – positive end-expiratory pressure

PROADI-SUS – Brazilian Unified Health System Institutional Development Support Program (Programa de Apoio ao Desenvolvimento Institucional do Sistema Único de Saúde)

SAPS – Simplified Acute Physology Score

SBP – systolic blood pressure

SOCATI – Santa Catarina Intensive Care Society (Sociedade Catarinense de Terapia Intensiva)

VIP – Ventilation, Infusion, and Pumping

WHO – World Health Organization

ABSTRACT

This is a cluster-randomized clinical trial of Brazilian intensive care units (ICUs). Up to 70 institutions will be selected to participate in the study, according to the criteria set forth in Brazilian Ministry of Health resolutions RDC 7/2010 and RDC 26/2012 and taking into account the reported volume of potential donors in the 2 years prior to selection. The institutions will be randomized in a 1:1 ratio to (1) Intervention Group: implementation of a goal-directed checklist based on the Brazilian guideline for maintenance of potential organ donors; or (2) Control Group: usual care. Randomization will be stratified according to the number of potential donors reported at each institution. Information will be collected from up to 30 potential consecutive organ donors at each institution. The primary outcome of interest will be the rate of cardiac arrest in potential donors.

Keywords: organ donation, cardiac arrest, goal protocol, cluster randomized clinical trial.

1 INTRODUCTION

Organ transplantation is the only therapeutic alternative for many patients with end-stage organ failure. Brazil has a well-established transplant program, but careful stewardship is required for the growth achieved in recent years to be sustained. This growth depends on the several professionals working in sequence, from the identification of potential multiple-organ donors to actual performance of the transplant itself. [1-4]

Brazil has the world's largest publicly funded and operated transplant system, performing approximately 20,000 procedures each year; the country accounts for the second highest absolute number of kidney (n = 5433) and liver (n = 1723) transplants. On the other hand, considering the size of the population, as of 2014 the country ranked 35th worldwide in kidney transplants (28.5 per million population, pmp) and 25th in liver transplants (9.0 pmp), with the vast majority of grafts coming from deceased donors (80%). [3] In 2014, only 8 states reached a rate of more than 15 donors pmp. Despite a 7.6% increase in 2014 (14.2 pmp), the donation rate was around 6% below the projection made in 2007 (15.0 pmp). This increase does not meet the continuous, growing demand of transplant waiting lists. The main causes of this mismatch between supply and demand for organs are family refusals and cardiac arrests in potential donors due to failure of clinical management. [1-3] In Brazil, it is estimated that up to 24.5% of potential valid donors are lost to failed management. This rate is still a long way from the 3.2% observed in the Spanish Model. [5].

In this context, the present study aims to assess whether implementation of a protocol for the ICU management of potential donors guided by a checklist of goals can reduce the loss of donors to cardiac arrest and thus increase the donor pool and the number of organs donated per donor.

1.1 Rationale

Preventing the unnecessary loss of potential organ donors to failure of proper clinical maintenance is essential to increasing the number of actual donors. [1,2,4] Clinical care protocols can be useful to prevent medical error, increase the likelihood of using evidence-based interventions, and improve patient outcomes.

Checklists have been widely used in aviation and manufacturing industry to avoid omissions while performing complex procedures. More recently, successful cases have been reported with the use of checklists in healthcare. Notable examples include use of the World Health Organization (WHO) Safe Surgery checklist, the Keystone ICU Project checklist to prevent central line-associated bloodstream infection, and a proposed checklist to improve processes of care in medical ICUs. [6,7,8]

Achieving care goals for potential donors is associated with a greater number of transplanted organs per donor and a reduction in donor losses to cardiac arrest. Salim et al (2005) demonstrated that an aggressive strategy for management of potential deceased donors reduced cardiac arrests in potential donors by 87% and increased the number of actual donors by 82%. [9] Molinoski et al (2012) and Patel et al (2014) demonstrated that the achievement of care goals guided by a checklist until the time of organ removal resulted in a greater number of organs donated per donor, and was an independent predictor of obtaining four or more organs. [10,11]

The Brazilian guidelines for maintenance of potential organ donors, published in 2011 [12,13], served as the basis for development of a maintenance checklist that was tested in a before-and-after pilot study conducted in two hospitals in the state of Santa Catarina. Adherence to the checklist items was associated with a reduction in the cardiac arrest rate in potential donors from 27.5% to zero. In addition, there was an increase in the actual donor rate from 40% (9 of 18) to 80% (17 of 20). [14].

We recently carried out a prospective observational study that evaluated the effectiveness of a program to improve the quality of care provided to potential organ donors in 27 hospitals across Santa Catarina between the years 2012 and 2014. The program included the development of a checklist of care goals and the training of teams from all state intrahospital transplant coordination (IHTC) in maintenance protocol management. The goal checklist served as a reference tool for case management. In addition, the ICU staff and IHTC teams of the eight hospitals with the highest number of reported cases of brain death in the state received on-site training led by instructors from the State Transplant Center and the Santa Catarina Intensive Care Society (SOCATI). [15] Over the past two years, the checklist has been improved, and now consists of nine goals distributed across three basic items that are part of the VIP shock management strategy: (1) mechanical Ventilation, (2) volume Infusion, and (3) evaluation of heart Pump effectiveness. [16] Implementation of the strategy led to a significant decline in losses to cardiac arrest (28% vs. 14.6; p=0.002) and an increase in the organ donation rate from 38% to 46%. Use of the checklist resulted in a reduced risk of loss of potential donors to cardiac arrest (OR 0.43, 95% CI 0.20–0.62, p<0.001). Adherence to more than four interventions on the management checklist reduced the odds of such loss 8.3-fold (OR 0.12; 95% CI 0.04–0.31, p<0.001). [15].

Thus, potential donor management based on goal checklists seems to contribute to a reduction in organ donor losses. However, the impressive results achieved with the use of checklists should be interpreted with caution due to non-random allocation of the intervention and the fact that analysis does not take the cluster effect into account. In addition, although some observational studies have shown an association between the use of potential donor maintenance checklists and an increase in the number of transplanted organs per donor, as well as a reduction in cardiac arrests, [9-11,14,15,17,18] there are no randomized, large-scale studies to prove these findings definitively.

1.2 Hypothesis

Implementation of a protocol for clinical management of potential organ donors guided by a checklist of goals would be able to reduce loss of potential donors to cardiac arrest, thus contributing to an increase in multiple organ donation rates.

2 OBJECTIVES

2.1 Primary objective

Assess whether implementation of a checklist-guided clinical management protocol for potential organ donors can reduce donor losses due to cardiac arrest.

2.2. Secondary objectives

Assess whether implementation of the checklist-guided clinical management protocol for potential donors can increase the number of actual organ donors and the number of donated organs per donor.

3 METHODS

3.1 Study design

Parallel cluster-randomized trial of intensive care units (ICUs) in Brazil.

3.2 Participants

Adult ICUs of Brazilian hospitals. Each cluster is made up of the set of participating ICUs from a single hospital.

3.3 Cluster eligibility criteria

Hospitals that have at least one adult ICU and reported at least 10 potential donors per year to their respective IHTC in the 2 years preceding selection (information confirmed through records provided by the Organ Notification, Procurement and Distribution Centers of all 26 states of Brazil and the Federal District to the National Transplant System), and which demonstrate institutional willingness to implement the protocol for clinical management of potential donors in at least one ICU will be eligible to participate in the study.

Coronary care units, step-down/high-dependency units, and emergency departments will not be eligible, nor will facilities that already have methods in place for management of potential donors like those proposed as the intervention of this study. Institutions that already use a protocol systematically for management of potential donors will be excluded. The criteria for identifying whether there is already systematic application of a goal-oriented protocol in place were: (1) Intended for management of all brain-dead potential donors; (2) Has been in use for at least 6 months; (3) Includes some form of warning device used at the bedside; (4) Documented in a printout or digital file; (5) Focused on actions and goals to be achieved; (6) Provides for real-time warning and activation of the ICU team.

3.4 Randomization of participating hospitals

The unit of randomization is the hospital. Hospital will be randomized into two groups: **Intervention** (up to 35) and **Control** (up to 35). Stratified block randomization will be performed, with block size varying depending on the expected number of brain deaths reported. Two strata will be considered: hospitals estimated to report up to 29 brain deaths and hospitals estimated to report more than 29 brain deaths. This cutoff point was defined considering the median number of brain deaths reported in the 2 years preceding selection.

3.5 Study arms

3.5.1 Intervention:

Application of a clinical management protocol for potential donors consisting of a goal-directed checklist.

Detailed description of the intervention: All sites in the intervention group must use the Checklist (a protocol based on the 2011 guidelines for maintenance of potential organ donors endorsed by the Brazilian Association of Intensive Care Medicine [AMIB], Brazilian Organ Transplantation Association [ABTO], and National Transplant System [SNT]), which consists of clinical decision support algorithms in the form of a goal-directed checklist. The main objective is to make the process easier for providers by briefly presenting management guidelines for potential donors. The first application of the Checklist should occur at the time of inclusion of the participant in the study, which will occur if he or she meets all of the eligibility criteria. The checklist will be reapplied every 6 hours from the moment of inclusion. Ideally, the checklist will be applied by the IHTC team, which will then alert the ICU team in charge of clinical management of any items that are not being met.

3.5.2 Control:

Management of potential donors in ICUs allocated to the control arm will be carried out as per routine local practice.

3.6 Co-intervention

Training will be offered, both in person and by remote learning tools, to provide guidance on the stages of the family interview for organ donation and communication in critical scenarios. All ICU and IHTC teams participating in the study, regardless of group allocation will receive this intervention.

3.7 Endpoints

3.7.1 Primary outcome:

Number of cardiac arrests (losses of potential donors due to cardiac arrest).

3.7.2 Secondary outcomes:

Number of actual donors.

Number of organs recovered per donor.

3.7.3 Exploratory outcomes related to care processes:

Core temperature ≥35°C in all potential donors with MAP >60 mmHg for more than 1 hour and no need to increase pressors; MAP ≥65 mmHg or SBP ≥90 mmHg; administration of vasopressin to all potential donors on noradrenaline or dopamine; urinary output 1–4 mL/kg; if on corticosteroids, blood glucose <180 mg/dL; PEEP ≥8 cmH2O; tidal volume 6–8 mL/kg; and sodium <155 mEq/L.

This session was updated in the published SAP to:

Exploratory outcomes according to the statistical analysis plan

1)	***Proportion of potential donors with adequate respiratory parameters, defined as PaO_2/FiO_2 ratio ≥200. In the absence of simultaneously measured PaO_2 and FiO_2, adequate respiration was defined as SaO_2/FiO_2 ≥240 (if positive end-expiratory pressure [PEEP] <8 cm H_2O), ≥259 (if PEEP 8–12 cm H_2O), or ≥234 (if PEEP >12 cm H_2O)[1] (presented as risk ratio [RR] and 95% confidence interval [CI]).***
2)	***Proportion of potential donors with adequate body temperature, defined as 34–35°C if hemodynamically stable and >35°C if mean arterial pressure (MAP) was <65 mm Hg or norepinephrine or dopamine was required (presented as RR and 95% CI).***
3)	***Proportion of potential donors with adequate circulatory parameters, considering the following as inadequate: MAP <65 mm Hg or norepinephrine ≥0.1 mcg/kg/min or dopamine ≥15 mcg/kg/min (presented as RR and 95% CI).***
4)	***Sequential Organ Failure Assessment (SOFA) score, as per Vincent et al. (1996)[2] (presented as mean difference [MD] and 95% CI).***
5)	***Proportion of potential donors receiving lung-protective ventilation: tidal volume (Vt) 6–8 mL/kg of predicted body weight and PEEP ≥8 cm H_2O (presented as RR and 95% CI).***
6)	***Proportion of potential donors receiving vasopressin if on norepinephrine or dopamine (presented as RR and 95% CI).***
7)	***Proportion of potential donors receiving hydrocortisone if on norepinephrine or dopamine (presented as RR and 95% CI).***
8)	***Proportion of potential donors with Na^+ <155 mEq/L (presented as RR and 95% CI).***
9)	***Proportion of potential donors with Mg^{++} >1.6 mEq/L (presented as RR and 95% CI).***
10)	***Proportion of potential donors with K^+ 3.5 to 5.5 mEq/L (presented as RR and 95% CI).***
11)	***Proportion of potential donors with capillary blood glucose <180 mg/dL (presented as RR and 95% CI).***
12)	***Proportion of potential donors receiving antibiotics, among those with infection (presented as RR and 95% CI).***

Source: Giordani NE, Robinson CC, Westphal GA, et al. Statistical analysis plan for a cluster-randomised trial assessing the effectiveness of implementation of a bedside evidence-based checklist for clinical management of brain-dead potential organ donors in intensive care units: DONORS (Donation Network to Optimise Organ Recovery Study). Trials. 2020;21(1):540.

[1] Pandharipande PP, Shitani AK, Hagerman HE, St Jacques PJ, Rice TW, Sanders NW, et al. Derivation and validation of Spo2/Fio2 ratio to impute for Pao2/Fio2 ratio in the respiratory component of the Sequential Organ Failure Assessment score. Crit Care Med. 2009;37(4):1317–21.21.

[2] Vincent JL, Moreno R, Takala J, Willatts S, De Mendonça A, Bruining H, et al. The SOFA (Sepsis-related Failure Assessment) score to describe organ disfunction/failure. Intensive Care Med. 1996;22:707–10.

3.8 Data collection

Data will be collected by the ICU and/or IHTC team, based on guidance provided during an induction visit. Data available on the medical records of up to 30 potential valid donors, as reported by the participating ICUs during the study period, will be collected.

3.8.1 Parameters of interest:

The following information on potential donors and provision of care will be collected from medical records when available:

- Identification of potential donor: Hospital record number, sex, date of birth;
- Background information: date and time of hospital admission, date and time of ICU

admission, reported or measured weight, predicted weight, height, cause of brain death, date and time of first clinical test of brain death, SAPS (Simplified Acute Physology Score) III on ICU admission;

- Respiratory variables: ventilation mode, tidal volume (mL), respiratory rate (bpm), PEEP (cmH_2O), plateau pressure if volume-controlled (cmH_2O), peak pressure (cmH_2O), FiO_2 (%), PaO_2 (mmHg), SaO_2 (%), $PaCO_2$ (mmHg);
- Hemodynamic variables: mean arterial pressure (mmHg), heart rate (bpm), noradrenaline or dopamine dose (mcg/kg/min), vasopressin dose (IU/hour), central venous pressure (mmHg), ΔPp (%), arterial lactate (mmol/L), central venous saturation (%), central venous CO_2 (mmHg), hemoglobin (g/dL);
- Fluid balance: fluids infused since first test for brain death (mL), urinary output since first test for brain death (mL), other fluid losses since first test for brain death (mL), water balance since the 1st test for ME (mL).
- Metabolic variables: creatinine (mg/dL), platelets ($/mm^3$), bilirubins (mg/dL), sodium (mEq/L), potassium (mEq/L), magnesium (mEq/L); phosphorus (mEq/L), calcium (mEq/L).
- Protocol endpoints: Organ removal, and which organs were donated; family refusal and causes thereof; cardiac arrest; contraindication due to poor clinical status.

The information will be collected after the first positive clinical evaluation of BD, as soon as the potential donor first presents criteria for the collection of study data, and at 6 hours, 12 hours, 24 hours, and every 24 hours thereafter, until one of the following occurs (whichever comes first): death from cardiac arrest; discontinuation of ventilatory support or cardiac arrest after confirmation of BD, until the time of organ removal; or the 7th day of follow-up since first data collection if cardiac arrest does not occur in the interim.

3.8.1 Eligibility criteria for potential donors

Inclusion criteria:

- Potential donors aged 14 to 90 years (all patients with suspected BD and who have a positive first clinical test for brain death (BD) will be considered potential donors);
- Admitted to ICU at the time of the first clinical test for BD or transferred to the ICU within 3 hours of the first clinical test for BD;
- Defined cause of BD;
- Body temperature 35°C or higher during the first clinical test for BD;
- Blood pressure (SBP) >90 mmHg during the first clinical test for BD;
- Proper washout time for any CNS depressants.
- First clinical examination consistent with diagnosis of BD.

Exclusion criteria:

- Positive HIV or HTLV-I/II serology;
- Acute hepatitis, malaria, acute viral infections (rubella, rabies, West Nile virus, adenovirus, enterovirus, parvovirus), meningoencephalitis of viral or unknown etiology, cryptococcal meningoencephalitis, prion diseases;
- Active tuberculosis with less than 2 months of treatment;
- Uncontrolled sepsis (active infection and pressor dose >1 mcg/kg/min noradrenaline or continuous fever despite 48 hours of antibiotic therapy);
- Donor colonization by bacteria resistant to all antibiotics;
- History of breast cancer, melanoma, soft-tissue sarcoma, hematological malignancies, or metastasis;
- Group 3 primary CNS neoplasm (according to the Operational Manual);
- Imminent cardiac arrest expected;
- First clinical examination for diagnosis of BD performed more than 3 hours before ICU admission.

3.8.3 Monitoring of collected data

All data will be reviewed by the trial coordinator, who may request supplemental information or correction of inconsistent information. The trial coordinator will also be responsible for a monthly review of all inclusions, data consistency, and form completeness. In addition to the remote monitoring carried out via digital or telephone contact, on-site visits to the participating institutions are expected to take place after the fifth participant included.

3.9 Sample and statistical analysis

3.9.1 Sample size

Up to 70 facilities, each including a minimum of 10 and a maximum of 30 potential donors, will be included. With 60 ICUs and an average of 20 potential donors enrolled per facility, or at least 1,200 potential donors in total, the study will have 80% statistical power (with a type I error rate of 5%), to detect an absolute reduction in cardiac arrests of 10% (from 28% in the control group to 18% in the experimental group), considering an intracluster coefficient (ICC) of 0.05 and an appropriate analysis, with adjustment for cluster effect. To ensure the minimum number of participating ICUs, we intend to screen at least 171 potential centers for eligibility and start the process of REC review of the study protocol in 80.

This session was updated in the published SAP to: With 60 ICUs and an average of 19 potential donors enrolled per facility, or at least 1140 potential organ donors in total (…). Considering a possible variation in cluster size and its impact on statistical power, we intend to include a minimum of 60 ICUs with at least 1200 potential organ donors, not allowing more than 30 participants in each cluster.

3.9.2 Statistical analysis

All statistical analyses will be based on the intention-to-treat principle. Thus, the data collected by the participating institutions will be analyzed according to the group to which they were allocated (e.g., institutions allocated to the intervention group will be analyzed in the intervention group), even if nonadherence or crossover (in whole or in part) occurs.

All statistical analyses will be based on the intention-to-treat principle. Thus, the data collected by the participating institutions will be analyzed according to the group to which they were allocated (e.g., institutions allocated to the intervention group will be analyzed in the intervention group), even if nonadherence or crossover (in whole or in part) occurs. Normality of data distribution will be assessed by visual inspection of histograms and application of the D'Agostino–Pearson test. Baseline characteristics will be described as absolute and relative frequencies, mean (SD), or median (IQR) as appropriate, for the intervention and control groups, using variables at both the cluster and individual levels. The primary outcome will be assessed by random effects logistic regression with adjustment for the baseline incidence of cardiac arrests. Secondary outcomes will be analyzed with generalized linear mixed models, adjusting the distribution of the outcome variable accordingly. All analyses of secondary outcomes will include adjustment for baseline values (measured 2 years prior to enrollment of each facility) of the corresponding variables.

Statistical analyses will be carried out in R software, version 3.5.2. We do not expect any substantial loss to follow-up for outcome data such as cardiac arrest, family refusals, and number of donations. Nevertheless, if loss to follow-up does occur, multiple imputation techniques will be used to make up for the missing data. For all other outcomes, analyses will be performed only on patients for whom complete data are available.

This session was updated in the SAP to: Normality of data distribution will be assessed by visual inspection of histograms. Baseline characteristics will be described as absolute and relative frequencies, mean (SD), or median (IQR) as appropriate, for the

intervention and control groups, using variables at both the cluster and individual levels. The primary outcome will be assessed by survival analysis adjusted for cluster effect (frailty model). Secondary outcomes will be analyzed with generalized estimating equations (GEE) with the appropriate distribution and adjustment for cluster effect - Poisson distribution with log-link function for estimation of risk ratio (RR) and Poisson distribution with identity link function for estimation of risk difference (RD). Analyses for primary and secondary outcomes will be based on participants for whom outcome data are available (that is, available case analysis). Thus, no imputation will be performed for primary or secondary outcomes.

3.10 Ethical aspects

The study was designed in compliance with Brazilian National Health Council Resolution No. 466/12. The study was approved by the Research Ethics Committee of the coordinating center (Associação Hospitalar Moinhos de Vento; opinion number 5399616.0.1001.5330). Individual approval will be requested from the ethics committees of each participating institution.

3.10.1 Risks

The only risks involving the conduct of this study concern breach of confidentiality and exposure of identifying information. To mitigate and/or prevent these risks, the principal investigator and other investigators involved in the study make an individual and collective commitment to use only data derived from the study, only for the purposes of this study, and to comply with the guidelines and regulatory standards set forth in Resolution No. 466/12 and other applicable resolutions, with respect to the secrecy and confidentiality of the data collected. Furthermore, individual information on the participating institutions and potential

donors will be anonymized; names will be replaced with numeric identifiers, and the most recent recommendations on confidentiality will be followed.

3.10.2 Benefits

If this study finds that implementation of the experimental intervention (which includes a maintenance checklist) is capable of improving outcomes, i.e., reducing the incidence of cardiac arrests in potential donors or other patient-relevant outcomes, the proposed interventions may be widely used in intensive care and transplant coordination, including in resource-constrained settings. In addition, if the intervention is found to be beneficial, institutions allocated to the control group will receive training in the use of the checklist at the end of the study.

3.10.3 Waiver of informed consent

Waiver of informed consent for the present study is based on the following arguments:

1) The DONORS study intervention will be applied in ICU settings and consists of an evidence-based clinical goal-directed checklist. Given the cluster design, the actual research participants will not be individual patients, but rather each intensive care unit in its entirety, which will be instructed to apply goal-directed checklist based on the 2011 guidelines for maintenance of potential organ donors endorsed by AMIB, ABTO, and SNT. The main objective is to facilitate the work of the ICU staff by briefly presenting management guidelines for potential donors, saving time, streamlining management, and improving the logistics of care provision. The intervention group will differ from the control only in implementation of a checklist that aims to systematize application of the aforementioned recommendations in order to increase incorporation of evidence-based practices into routine care. Current best recommendations for clinical management of potential donors will be available to both groups.

2) Group allocation will occur by hospital (cluster), not by participant. Therefore, we will request written institutional assent from the facility and the ICU coordinator by means of the Institutional Assent Form before the study is conducted.

4 EXPECTED RESULTS

In the present study, we expect to observe significantly lower rates of cardiac arrest among potential donors managed at hospitals in the intervention group. The study protocol may undergo adjustments, which will be previously submitted to the Research Ethics Committee for evaluation.

5. ORGANIZATIONAL ASPECTS OF THE STUDY

Hospital Moinhos de Vento is the sponsor and coordinator of the study, through the Unified Health System Institutional Development Support Program (PROADI-SUS), under the supervision of the General Coordination of the Brazilian National Transplant System (CGSNT). The study is supported by the Brazilian Research in Intensive Care Network (BRICNet), the Organ Donation and Transplant Committee of the Brazilian Association of Intensive Care Medicine (AMIB), the Brazilian Organ Transplantation Association (ABTO), the National Transplant Organization of Spain (ONT), and the various State Organ Notification, Procurement, and Distribution Centers.

The study Steering Committee is composed of critical care specialists, transplant coordinators, and epidemiologists with experience in conducting multicenter trials. This committee has been and will be involved in the conception, design, and supervision of the study, as well as in writing the final manuscript.

6 REFERENCES

1. The Madrid resolution on organ donation and transplantation: national responsibility in meeting the needs of patients, guided by the WHO principles. Transplantation. 2011;91 Suppl 11:S29-31.

2. España. Ministério de Sanidad, Política Social e Igualdad. Organización Nacional de Trasplantes. Guía de buenas prácticas en el proceso de La donación de órganos. España; 2011. Available at: http://www.ont.es/publicaciones/Documents/GUIA_BUENAS_PRACTICAS_DONACION_ORGANOS.pdf

3. Associação Brasileira de Transplantes de Órgãos (ABTO). Dimensionamento dos Transplantes no Brasil e em cada estado (2006-2013). 2013; 19(4). Available at: http://www.abto.org.br/abtov03/Upload/file/RBT/2014/rbt2014-lib.pdf

4. DuBose J, Salim A. Aggressive Organ Donor Management Protocol. J Intensive Care Med 2008 23: 367-375

5. Knihs NS Schirmer J, Roza BA. Adaptación del modelo español de gestión en trasplante para la mejora en la negativa familiar y mantenimiento del donante potencial. Texto Contexto Enferm2011; 20 (Esp): 59-65.

6. Haynes AB1, Weiser TG, Berry WR et al. A surgical safety checklist to reduce morbidity and mortality in a global population. N Engl J Med. 2009 Jan 29;360(5):491-9.

7. Pronovost P1, Needham D, Berenholtz S et al. An intervention to decrease catheter-related bloodstream infections in the ICU. N Engl J Med 2006;355:2725- 32

8. Weiss CH, Moazed F, McEvoy CA et al. Prompting physicians to address a daily checklist and process of care and clinical outcomes: a single-site study. Am J Respir Crit Care Med. 2011; 184(6):680-6.

9. Salim A, Velmahos GC, Brown C, et al. Aggressive organ donor management significantly increases the number of organs available for transplantation. J Trauma 2005;58(5):991-4.

10. Malinoski DJ, Patel MS, Daly MC, Oley-Graybill C, Salim A; UNOS Region 5 DMG workgroup. The impact of meeting donor management goals on the number of organs transplanted per donor: results from the United Network for Organ Sharing Region 5 prospective donor management goals study. Crit Care Med. 2012 Oct;40(10):2773-80.

11. Patel MS, Zatarain J, De La Cruz S, et al. The Impact of Meeting Donor Management Goals on the Number of Organs Transplanted per Expanded Criteria Donor: A Prospective Study From the UNOS Region 5 Donor Management Goals Workgroup. JAMA Surg. 2014 Sep 1;149(9):969-975

12. Westphal GA, Caldeira Filho M, Vieira KD, Zaclikevis VR, Bartz MC, Wanzuita R, et al. Guidelines for potential multiple organ donors (adult). Part I. Rev Bras Ter Intensiva. 2011;23(3):255-68.

13. Westphal GA, Caldeira Filho M, Fiorelli A, Vieira KD, Zaclikevis VR et al. Guidelines for maintenance of adult patients with brain death and potential for multiple organ donations: the Task Force of the Brazilian Association of Intensive Medicine the Brazilian Association of Organs Transplantation, and the Transplantation Center of Santa Catarina. Transplant Proc. 2012 Oct;44(8):2260- 7.

14. Westphal GA, Zaclikevis VR, Vieira. KD et al.A managed protocol for treatment of deceased potential donors reduces the incidence of cardiac arrest before organ explant. Rev Bras Ter Intensiva. 2012; 24(4):334-340

15. Machado MC, Montemezzo A, Cani F et al. Meeting clinical goals for the maintenance of the potential organ donor can reduce the loss of donors by cardiac arrest. Crit Care 2015: 19(Suppl 2): P88. [Abstract].

16. Westphal GA. A simple bedside approach to therapeutic goals achievement during the management of deceased organ donors - An adapted version of the "VIP" approach. Clin Transplant. 2016 Feb;30(2):138-44

17. Helms AK, Torbey MT, Hacein-Bey L, et al. Standardized protocols increase organ and tissue donation rates in the neurocritical care unit. Neurology. 2004;63(10):1955-7.

18. Franklin GA1, Santos AP, Smith JW. Optimization of Donor Management Goals Yields Increased Organ Use. Am Surg 2010 Jun;76(6):587-94

19. Malinoski DJ, Daly MC, Patel MS, Oley-Graybill C, Foster CE 3rd, Salim A. Achieving donor management goals before deceased donor procurement is associated with more organs transplanted per donor. J Trauma. 2011 Oct;71(4):990-5.

20. Diretrizes para avaliação e validação do potencial doador de órgãos em morte encefálica (AMIB/ABTO). Rev Bras Ter Intensiva 2016 [Submetido]

21. Rech TH, Rodrigues EM. Entrevista familiar e consentimento. Rev Bras Ter Intensiva 2007; 19(1)

22. Vincent A, Logan L. Consent for organ donation. Br J Anaesth 2012; 108 (suppl 1): i80-i87

23. Carmen Segovia, Manuel Serrano. Comunicación en Situaciones Críticas. Organización Nacional de Trasplantes de España. Available at: http://agora.ceem.org.es/wpcontent/uploads/documentos/bioetica/comunicacionensituacionescriticasONT.pdf

24. Lautrette A, Darmon M, Megarbane B et al. A Communication Strategy and Brochure for Relatives of Patients Dying in the ICU. N Engl J Med 2007;356:469- 78.

25. Curtis JR, Patrick DL, Shannon SE. The family conference as a focus to improve communication about end-of-life care in the intensive care unit: Opportunities for improvement. Crit Care Med 2001; 29[Suppl.]:N26–N33

26. Niemann CU, Feiner J, Swain S et al. Therapeutic hypothermia in deceased organ donors and kidney-graft function. N Engl J Med 2015:373:405.

ASSOCIAÇÃO HOSPITALAR MOINHOS DE VENTO

DONORS - ICU Trial

Donation Network to Optimize Organ Recovery Study

Study registry: ClinicalTrials.gov number, NCT03179020.

Statistical analysis plan

Principal Statistician: Natalia Elis Giordani, MSc.

Porto Alegre, 2018

Summary

1. Sample size 3
2. Randomization 3
3. Overall principles 3
4. Flow of participants 4
5. Adherence to study intervention 4
6. Missing data 4
7. Baseline characteristics 5
8. Primary outcome 5
9. Secondary outcomes 7
10. Exploratory outcomes 7

References 9

Appendix 1 10

Appendix 2 11

1. **Sample size**

To detect an absolute reduction of donor losses due to cardiac arrests of 10% (from 28% in the control group to 18% in the intervention group) it is necessary 60 hospitals and 1140 potential donors (19 brain-dead potential organ donors per site)[1], considering an intraclass correlation coefficient of 0.05, power of 80%, and a two-sided alpha level of 5%. Considering a possible variation in cluster size and its impact on statistical power, we intend to include a minimum of 60 hospitals with at least 1200 potential organ donors, not allowing more than 30 participants in each cluster. WinPepi software version 11.65 (http://www.brixtonhealth.com/pepi4windows.html) and the StatsToDo website (www.statstodo.com/index.php) were used for sample size determination.

2. **Randomization**

Randomization will occur at the cluster level. To avoid contamination, the group of the adult intensive care units willing to participate in the study in an included hospital is considered a cluster. Brazilian hospitals with an average of at least ten annual notifications of potential organ donors (neurologic criteria) in the 2 years preceding study selection will be randomly allocated to the intervention or control group in a 1:1 ratio using blocks of variable sizes (2 and 4), then stratified dichotomously by the estimated median number of annual notifications of brain-dead potential organ donors (≤ 29 vs. > 29 notifications). Consecutive brain-dead potential organ donors (as confirmed by the first clinical examination consistent with brain death) aged 14–90 years will be screened. Only patients already in the ICU or admitted to the ICU within 3 hours of initial assessment for brain death will be included. A statistician will be responsible for the randomization processes.

3. **Overall principles**

Analysis will start when all data have been obtained from the last included patient, the database has been cleaned and locked, and the plan has been accepted for publication. Interim analysis will not be conducted. All analyses will be performed in the R software environment (R Foundation for Statistical Computing).[2]

At the cluster level, the population includes all randomised hospitals that recruited at least one brain-dead potential organ donor. At the subject level, the population includes up to 30 consecutive brain-dead potential donors in each cluster.

All potential donors will be included in the analysis and will be analysed according to their allocated treatment group (control or intervention), regardless of the adherence to the protocol (intention to treat). The main analysis for each outcome will be performed at the potential organ donor level. All analyses will account for the cluster-randomised design, thus ensuring correct type I error rates and confidence intervals. A significance level of 0.05, adjusted for multiplicity as appropriate, will be adopted for all comparisons.

4. Flow of participants

The flow of participants will be presented displayed in a detailed diagram that meets the criteria of the Consolidated Standards of Reporting Trials (CONSORT) extension for cluster-randomised trials. The description will include information on eligibility criteria and loss to follow-up at both the cluster and subject levels.

5. Adherence to study intervention

The intervention consists of the use of the checklist for management of brain-dead potential organ donors with 13 goals and 14 actions. Adherence to each specific action will be considered complete if the recommended course of action was performed or (b) there was no need for action according to the checklist. Adherence to each individual component will be presented as a proportion, considering the ratio between the total number of actions adhered and the total number of potential actions to be performed. Estimation of adherence will consider the checklists applied at baseline, 6 h, 12 h, 24 h, 48 h, 72 h, 96 h, 120 h, 144 h, and 168 h as available.

6. Missing data

We expect missing values for exploratory outcomes. Nevertheless, the coordinating centre will contact site investigators to retrieve any missing data values or data will be monitored by in-site visits.

For exploratory outcomes, data imputation will be performed for the SOFA score components, as follow:

- For all SOFA components, the information from the latest available time point of assessment will be imputed;
- For the respiratory component, if the partial oxygen pressure (PaO_2)/fraction of inspired oxygen (FIO_2) ratio was not available, this score will be estimartedon the

basis of peripheral oxygen saturation (SaO_2)/FIO_2 ratio adjusted to the positive end-expiratory pressure (PEEP), according to a previously validated method.[3]

- For the coagulation, liver, and renal components, if there are missing values at any point during follow-up, we will impute a score of 0 (corresponding to normality), except if pre-existing comorbidities are present (participants with renal impairment requiring dialysis will be imputed a score of 4 and participants with liver cirrhosis a score of 2).[4]

7. Baseline characteristics

The baseline characteristics of all participants, stratified by study arm, will be presented in Table 1 (Appendix 1). Continuous variables will be presented as mean and standard deviation (SD) or median and interquartile range (IQR), as appropriate. Categorical variables will be presented as absolute (*n*) and relative (%) frequencies. Statistical comparisons between groups will not pe conducted.

8. Primary outcome

The primary outcome is loss of brain-dead potential organ donors to cardiac arrest (defined as any loss from irreversible or unreversed cardiac arrest that occurs after patient enrolment while the potential donor remains eligible for organ donation, i.e., with no contraindications and after family approval or with family decision pending). Data will be recorded up to 14 days after participant enrolment. Loss to cardiac arrest occurring after family refusal for organ donation or detection of a contraindication to donation will not be recorded as a primary outcome. To assess the effect of study interventions on loss due to cardiac arrest, we will use survival analysis adjusted for cluster effect (frailty model).[5] Participants will be considered at risk for occurrence of the outcome of interest only while under consideration as brain-dead potential organ donors. Thus, data will be censored in the following circumstances: (a) family refusal, (b) contraindications to organ donation, or (c) organ retrieval. Results will be presented as hazard ratio (HR) and 95% confidence interval (CI). Model assumptions (e.g., proportional hazards and residuals analysis) will be assessed using appropriate tests and plots. Primary outcome will be presented as shown in Table 2 (Appendix 2).

Sensitivity analyses

Sensitivity analysis of the primary outcome will be adjusted for:

1) adherence to study intervention;
2) time elapsed between first clinical examination consistent with brain death and inclusion in the study;
3) occurrence of failures in the screening of consecutive potential organ donors;
4) estimated number of brain death notifications in each hospital (≤ 29 vs. > 29, according to the stratification variable);
5) donation rate for each site before the study.

For analyses 1 and 2, the cut-off will be the median observed across sites. For analysis 3, in order to estimate the number of recruitment failures per site, the total number of brain death notifications according to the Brazilian National Transplant System records during the study period will be considered. The outcome will be simulated considering a binomial distribution with probability of success equal to the percentage of losses of brain-dead potential organ donors due to cardiac arrest observed in the control group. The total follow-up time for each recruitment failure will be simulated considering the minimum, median, and maximum values observed in the sites, according to the observed simulated outcome. Analysis 4 will consider the dichotomous stratification used for random allocation, based on the estimated annual number of notifications of brain death in each site (≤ 29 vs. > 29). Analysis 5 will consider the donation rate for each centre for the year 2016, which is available in the Brazilian National Transplant System records.

Subgroup analyses

There will be three subgroup analyses defined for the primary outcome, considering the variables age > 60 years, cause of the insult leading to potential brain death (traumatic vs. non-traumatic), and patient severity upon ICU admission defined by the Simplified Acute Physiology Score 3 (SAPS 3; cut-off will be established regarding the overall median score). The consistency of intervention effects across the mentioned subgroups will be assessed by tests of interaction. The Bonferroni correction will be applied to adjust the multiple subgroup analysis. With the conduction of three tests will be performed the critical alpha will be 0.017.

9. Secondary outcomes

Number of actual organ donors, indexed to brain-dead potential donors (proportion): differences in actual donor ratios between the intervention and control groups will be analysed using generalised estimating equations (GEE), with the appropriate distribution and adjustment for cluster effect (Poisson distribution with log-link function for estimation of risk ratio [RR] and Poisson distribution with identity link function for estimation of risk difference [RD][5,6,7,8,9]. This outcome will be presented as RR and RD with 97.5% CI, adjusted for multiple comparisons by Bonferroni correction. We will conduct a sensitivity analysis considering the number of kidneys harvested.

Number of solid organs recovered per actual donor (from zero to seven organs per donor, as follows: liver, heart, pancreas, two lungs, and two kidneys):
Between-group differences in the mean number of solid organs recovered per actual donor will be compared using GEE with the appropriate distribution and adjustment for cluster effect (Poisson distribution with identity link function). This outcome will be presented as mean difference (MD) and 97.5% CI adjusted for multiple comparisons with Bonferroni correction. The Bonferroni correction will be used to adjust the analyses of secondary outcomes for multiplicity, considering two comparisons of interest; thus, the critical alpha will be 0.025.

Secondary outcomes will be presented as shown in Table 2 (Appendix 2).

10. Exploratory outcomes

1. Proportion of potential donors with adequate respiratory parameters, defined as PaO_2/FIO_2 ratio ≥ 200. In the absence of PaO_2 and FIO_2 parameters measured simultaneously, adequate respiration will be defined as $SaO_2/FIO_2 \geq 240$ (if PEEP < 8), ≥ 259 (if PEEP 8–12), or ≥ 234 (if PEEP > 12)[3] (will be presented as RR and 95% CI).
2. Proportion of potential donors with adequate body temperature, defined as 34–35 °C if haemodynamically stable and > 35 °C if mean arterial pressure (MAP) < 65 mmHg or noradrenaline or dopamine is required (will be presented as RR and 95% CI).

3. Proportion of potential donors with adequate circulatory parameters, considering the following as inadequate: MAP < 65 mmHg or noradrenaline ≥ 0.1 µg/kg/min or dopamine ≥15 µg/kg/min (will be presented as RR and 95% CI).
4. SOFA score[4] (will be presented as MD and 95% CI).
5. Proportion of potential donors receiving protective ventilation: tidal volume (Vt) 6–8 ml/kg of predicted body weight and PEEP ≥ 8 cmH_2O (will be presented as RR and 95% CI).
6. Proportion of potential donors receiving vasopressin if on noradrenaline or dopamine (will be presented as RR and 95% CI).
7. Proportion of potential donors receiving hydrocortisone if on noradrenaline or dopamine (will be presented as RR and 95% CI).
8 Proportion of potential donors with Na < 155 mEq/L (will be presented as RR and 95% CI).
9. Proportion of potential donors with Mg > 1.6 mEq/L (will be presented as RR and 95% CI).
10. Proportion of potential donors with K 3.5–5.5 mEq/L (will be presented as RR and 95% CI).
11. Proportion of potential donors with capillary blood glucose < 180 mg/dl (will be presented as RR and 95% CI).
12. Proportion of potential donors receiving antibiotics (among those with infection). Will be presented as RR and 95% CI.
13. All exploratory outcomes will be analysed using a repeated-measures GEE considering each 24-h fraction of the follow-up period (Poisson distribution with log-link function for outcomes 1, 2, 3, and 5 to 12 and normal distribution with identity link function for outcome 4), with exception of exploratory outcome number 12.
No adjustment for multiple comparisons will be performed for exploratory outcomes.

References

1 Westphal GA, Coll E, de Souza RL, Wagner S, Montemezzo A, Cani de Souza FC, et al. Positive impact of a clinical goal-directed protocol on reducing cardiac arrests during potential brain-dead donor maintenance. Crit Care. 2016;20:323.

2 R Core Team. R: a language and environment for statistical computing. Vienna: R Foundation for Statistical Computing; 2018. https://www.R-project.org/. Accessed 29 Jul 2019.

3 Pandharipande PP, Shitani AK, Hagerman HE, St Jacques PJ, Rice TW, Sanders NW, et al. Derivation and validation of Spo2/Fio2 ratio to impute for Pao2/Fio2 ratio in the respiratory component of the Sequential Organ Failure Assessment score. Crit Care Med. 2009;37(4):1317–21.

4 Vincent JL, Moreno R, Takala J, Willatts S, De Mendonça A, Bruining H, et al. The SOFA (Sepsis-related Failure Assessment) score to describe organ disfunction/failure. Intensive Care Med. 1996;22:707–10.

5 Collet D. Modelling survival data in medical research. 2nd ed. Boca Raton: Chapman and Hall/CRC; 2003.

6 Twisk JWR. Applied longitudinal data analysis for epidemiology: a practical guide. New York: Cambridge University Press; 2003.

7 Pedroza C, Thanh Trong VT. Performance of models for estimating absolute risk difference in multicenter trials with binary outcome. BMC Med Res Methodol. 2016;16:113.

8 Leyrat C, et al. Cluster randomized trials with a small number of clusters: which analyses should be used? Int J Epidemiol. 2018;47(1):321–31.

9 Pedroza C, Truong VTT. Estimating relative risks in multicenter studies with a small number of centers — which methods to use? A simulation study. Trials. 2017;18:512.

Appendix 1

Table 1. Baseline characteristics of study participants (sites and potential organ donors)

	Intervention arm	Control arm
Hospital characteristics		
Number of hospital beds, central tendency (dispersion), [n]	xx.x (xx.x)	xx.x (xx.x)
Number of ICU beds, central tendency (dispersion), [n]	xx.x (xx.x)	xx.x (xx.x)
Number of ICU beds/hospital beds, central tendency (dispersion), [n]	xx.x (xx.x)	xx.x (xx.x)
Type of ICU		
Surgical, n/total (%)	xx/xx (xx.x)	xx/xx (xx.x)
Medical, n/total (%)	xx/xx (xx.x)	xx/xx (xx.x)
Mixed, n/total (%)	xx/xx (xx.x)	xx/xx (xx.x)
Hospital type		
Public, n/total (%)	xx/xx (xx.x)	xx/xx (xx.x)
Private, n/total (%)	xx/xx (xx.x)	xx/xx (xx.x)
Teaching activity, n/total (%)	xx/xx (xx.x)	xx/xx (xx.x)
Transplant centre, n/total (%)	xx/xx (xx.x)	xx/xx (xx.x)
Number of brain death notifications per year[1], central tendency (dispersion), [n]	xx.x (xx.x)	xx.x (xx.x)
Participant characteristics		
Age in years, central tendency (dispersion), [n]	xx.x (xx.x)	xx.x (xx.x)
Age > 60 years, n/total (%)	xx/xx (xx.x)	xx/xx (xx.x)
Female sex, n/total (%)	xx/xx (xx.x)	xx/xx (xx.x)
Male sex, n/total (%)	xx/xx (xx.x)	xx/xx (xx.x)
SAPS 3 score at ICU admission, central tendency (dispersion), [n]	xx.x (xx.x)	xx.x (xx.x)
Comorbidities		
Diabetes mellitus, n/total (%)	xx/xx (xx.x)	xx/xx (xx.x)
Hypertension, n/total (%)	xx/xx (xx.x)	xx/xx (xx.x)
Dialytic renal failure, n/total (%)	xx/xx (xx.x)	xx/xx (xx.x)
Chronic respiratory disease, n/total (%)	xx/xx (xx.x)	xx/xx (xx.x)
Heart failure, n/total (%)	xx/xx (xx.x)	xx/xx (xx.x)
Chronic liver disease, n/total (%)	xx/xx (xx.x)	xx/xx (xx.x)
Cause of brain injury		
Trauma, n/total (%)	xx/xx (xx.x)	xx/xx (xx.x)
Stroke, n/total (%)	xx/xx (xx.x)	xx/xx (xx.x)
Anoxia, n/total (%)	xx/xx (xx.x)	xx/xx (xx.x)
Other, n/total (%)	xx/xx (xx.x)	xx/xx (xx.x)
SOFA score at enrolment, central tendency (dispersion), [n]	xx.x (xx.x)	xx.x (xx.x)
Use of antimicrobial medication[2], n/total (%)	xx/xx (xx.x)	xx/xx (xx.x)
Length of hospital stay in days before brain death diagnosis, central tendency (dispersion), [n]	xx.x (xx.x)	xx.x (xx.x)

[1] Number of brain death notifications per year considers the percentage of brain-dead potential organ donors clinically managed in the intensive care unit.
[2] Identified at the time of first clinical examination
SAPS: Simplified Acute Physiology Score. Chronic respiratory disease: Restrictive, obstructive, or vascular, severe enough to limit performance of the activities of daily living; or chronic hypoxia, hypercapnia, polycythaemia, pulmonary hypertension, or ventilator dependence. Chronic liver disease: Biopsy-proven cirrhosis or proven portal hypertension or previous history of hepatic insufficiency, encephalopathy, or coma. SOFA: Sequential Organ Failure Assessment.

Appendix 2

Table 2. Primary and secondary study outcomes.

Outcomes	Intervention arm	Control arm	Type of effect estimate	Effect estimate (CI)	*p*-value[a]
Primary					
Potential organ donors lost due to cardiac arrest, n/total (%)[b]	x/x (xx.x)	x/x (xx.x)	HR	x.xx (x.xx-x.xx)*	x.xx
Secondary					
Actual organ donors, n/total (%)	x/x (xx.x)	x/x (xx.x)	RR<br>RD	x.xx (x.xx-x.xx)**<br>x.xx (x.xx-x.xx)**	x.xx
Organs recovered per actual donor, central tendency (dispersion)	xx.x (xx.x)	xx.x (xx.x)	MD	x.xx (x.xx-x.xx)**	x.xx

CI: Confidence interval. HR: Hazard ratio. RR: Risk ratio. RD: Risk difference. MD: mean difference.

[a] Adjusted for multiple comparisons with Bonferroni correction when appropriate

* 95% confidence interval

** 97.5% confidence interval

[b] Intracluster correlation coefficient
