# Supplementary material for: Evidence-Based Checklist to Delay Cardiac Arrest in Brain-Dead Potential Organ Donors: The DONORS Cluster Randomized Clinical Trial
Source: JAMA Netw Open. 2023 Dec 14;6(12):e2346901. doi: 10.1001/jamanetworkopen.2023.46901 (PMC10722341; doi:10.1001/jamanetworkopen.2023.46901)
Supplement: Supplement 2. — eTable 1. Exclusion Criteria for Brain-Dead Potential Organ Donors and Number of Exclusion Criteria Identified in Each Study Arm eFigure 1. Logic Model for Study Development eFigure 2. Description of the Adherence Measurement eMethods 1. Elaboration of the Evidence-Based Clinical Practice Guideline for the Management of Potential Brain-Dead Donors eTable 2. Family Interview Support Guide Based on the Spanish Model of Communication in Critical Situations eMethods 2. Exploratory Outcomes According to the Statistical Analysis Plan eTable 3. Post Hoc Analyses and Reasons for Their Selection eFigure 3. Geographic Distribution of the Included Sites eTable 4. Post Hoc Analysis Considering Adherence to the Intervention According to the Time of Brain-Dead Potential Donor Inclusion eFigure 4. Subgroup Analysis of the Primary Outcome (Loss of Brain-Dead Potential Organ Donors Due to Cardiac Arrest) eTable 5. Sensitivity Analysis for the Primary Outcome eTable 6. Sensitivity Analysis of Secondary Outcomes Regarding Adherence Considering Potential Organ Donor Adherence to the Intervention eTable 7. Characteristics of the Brain-Dead Potential Organ Donors at Baseline Considering Adherence to the Intervention at the Potential Organ Donor Level eTable 8. Characteristics of the Sites and the Brain-Dead Potential Organ Donors at Baseline Considering Site Adherence to the Intervention at the Site Level eTable 9. Post Hoc Analysis of Secondary Outcomes Regarding Adherence Considering Site Adherence to the Intervention eTable 10. Post Hoc Analysis for the Primary Outcome Combining Site and Brain-Dead Potential Organ Donor Adherence to the Intervention eTable 11. Comparison of Physiologic and Treatment Goals at Baseline Between the Intervention and Control Groups and Between High (>77.5%) and Low Adherence (≤77.5%) Centers eTable 12. Comparison of Goal Achievement Between the Intervention and Control Groups eTable 13. Individual Adherence to the Goals Over Time in the Intervention Group [file jamanetwopen-e2346901-s002.pdf]

Supplemental Online Content

Westphal GA, Robinson CC, Giordani NE, et al; DONORS (Donation Network to Optimize Organ Recovery Study) Investigators, BRICNet (Brazilian Research in Intensive Care Network). Evidence-based checklist to delay cardiac arrest in brain-dead potential organ donors: the DONORS cluster randomized clinical trial. *JAMA Netw Open*. 2023;6(12):e2346901. doi:10.1001/jamanetworkopen.2023.46901

eTable 1. Exclusion Criteria for Brain-Dead Potential Organ Donors and Number of Exclusion Criteria Identified in Each Study Arm

eFigure 1. Logic Model for Study Development

eFigure 2. Description of the Adherence Measurement

eMethods 1. Elaboration of the Evidence-Based Clinical Practice Guideline for the Management of Potential Brain-Dead Donors

eTable 2. Family Interview Support Guide Based on the Spanish Model of Communication in Critical Situations

eMethods 2. Exploratory Outcomes According to the Statistical Analysis Plan

eTable 3. Post Hoc Analyses and Reasons for Their Selection

eFigure 3. Geographic Distribution of the Included Sites

eTable 4. Post Hoc Analysis Considering Adherence to the Intervention According to the Time of Brain-Dead Potential Donor Inclusion

eFigure 4. Subgroup Analysis of the Primary Outcome (Loss of Brain-Dead Potential Organ Donors Due to Cardiac Arrest)

eTable 5. Sensitivity Analysis for the Primary Outcome

eTable 6. Sensitivity Analysis of Secondary Outcomes Regarding Adherence Considering Potential Organ Donor Adherence to the Intervention

eTable 7. Characteristics of the Brain-Dead Potential Organ Donors at Baseline Considering Adherence to the Intervention at the Potential Organ Donor Level

eTable 8. Characteristics of the Sites and the Brain-Dead Potential Organ Donors at Baseline Considering Site Adherence to the Intervention at the Site Level

eTable 9. Post Hoc Analysis of Secondary Outcomes Regarding Adherence Considering Site Adherence to the Intervention

eTable 10. Post Hoc Analysis for the Primary Outcome Combining Site and Brain-Dead Potential Organ Donor Adherence to the Intervention

eTable 11. Comparison of Physiologic and Treatment Goals at Baseline Between the Intervention and Control Groups and Between High (>77.5%) and Low Adherence (≤ 77.5%) Centers

eTable 12. Comparison of Goal Achievement Between the Intervention and Control Groups

eTable 13. Individual Adherence to the Goals Over Time in the Intervention Group Comparing Participants Whose Physiology Met Parameters at the Baseline to Those Who Did Not Meet Parameters

eFigure 5. Correlation Between Adherence to the Intervention per Site and at Individual Level (Potential Donor Adherence)

eTable 14. Descriptive Analysis for a Proxy for Site Quality

eTable 15. Post Hoc Analysis by Proxy for Site Quality Using a Directed Acyclic Graph (DAG)

eTable 16. Post Hoc Analysis Adjusted for the Characteristics of Sites and Brain-Dead Potential Organ Donors Considering Site Adherence to the Intervention and Using a Directed Acyclic Graph (DAG)

This supplemental material has been provided by the authors to give readers additional information about their work.

© 2023 Westphal GA et al. *JAMA Network Open*

eTable 1. Exclusion criteria for brain-dead potential organ donors and number of exclusion criteria identified in each study arm

Criterion	Arm	
	Intervention	Control
Age <14 or >90 years	5	3
Not clinically managed in the ICU		
Not admitted to the ICU	16	9
First clinical examination consistent with brain death, but completed more than 3 hours before ICU admission	7	10
Failure to complete the diagnosis of brain death		
Undefined cause of brain injury	0	0
Body temperature ≤35°C during the first clinical examination for brain death diagnosis	8	10
Systolic blood pressure >90 mm Hg during the first clinical examination for brain death diagnosis	1	0
Withdrawal time for central nervous system depressants was respected	0	0
First clinical examination not consistent with brain death	1	1
Cancer-related contraindications		
Metastatic cancer, breast tumors, melanoma, soft tissue sarcoma, hematologic malignancy	19	14
Primary tumors of the central nervous system – Group 3 (anaplastic astrocytoma – grade III, glioblastoma multiforme, medulloblastoma, anaplastic oligodendroglioma – Schmidt C and D, malignant ependymoma, pineoblastoma, anaplastic/malignant meningioma, intracranial sarcoma, germ cell tumor – except well-differentiated teratoma, chordoma, and primary cerebral lymphoma)	15	22
Infectious contraindications		
Human immunodeficiency virus (HIV), human T-cell lymphotropic virus (HTLV)-I and II	18	9
Acute hepatitis, malaria, acute viral infections (e.g., rubella, rabies, West Nile virus, adenovirus, enterovirus, parvovirus, and viral meningoencephalitis or of unknown cause), cryptococcal meningoencephalitis, Prion diseases	10	14
Active tuberculosis with <2 months of treatment	4	4
Uncontrolled sepsis	20	28
Bacterial colonization of the donor without antibiotic treatment options (resistant to all antibiotics)	8	4
Contraindications due to hemodynamic instability		
Severe clinical instability with the expectation of imminent cardiac arrest	9	8
Other contraindications		
Contraindication to donation by the State Transplant Center	8	1
**Total number of patients not included (% in relation to screened)**	119 (13.8%)	117 (12.9%)

Note: Exclusion criteria were grouped by affinity (italics) for flowchart summary (figure 1 of the main document). The sum of each category is different from that presented in the flowchart because some screened patients met more than one exclusion criterion. The flowchart provides the first criterion met by the patient according to the study protocol, following the order presented in the table.

© 2023 Westphal GA et al. *JAMA Network Open*

eFigure 1. Logic model for study development

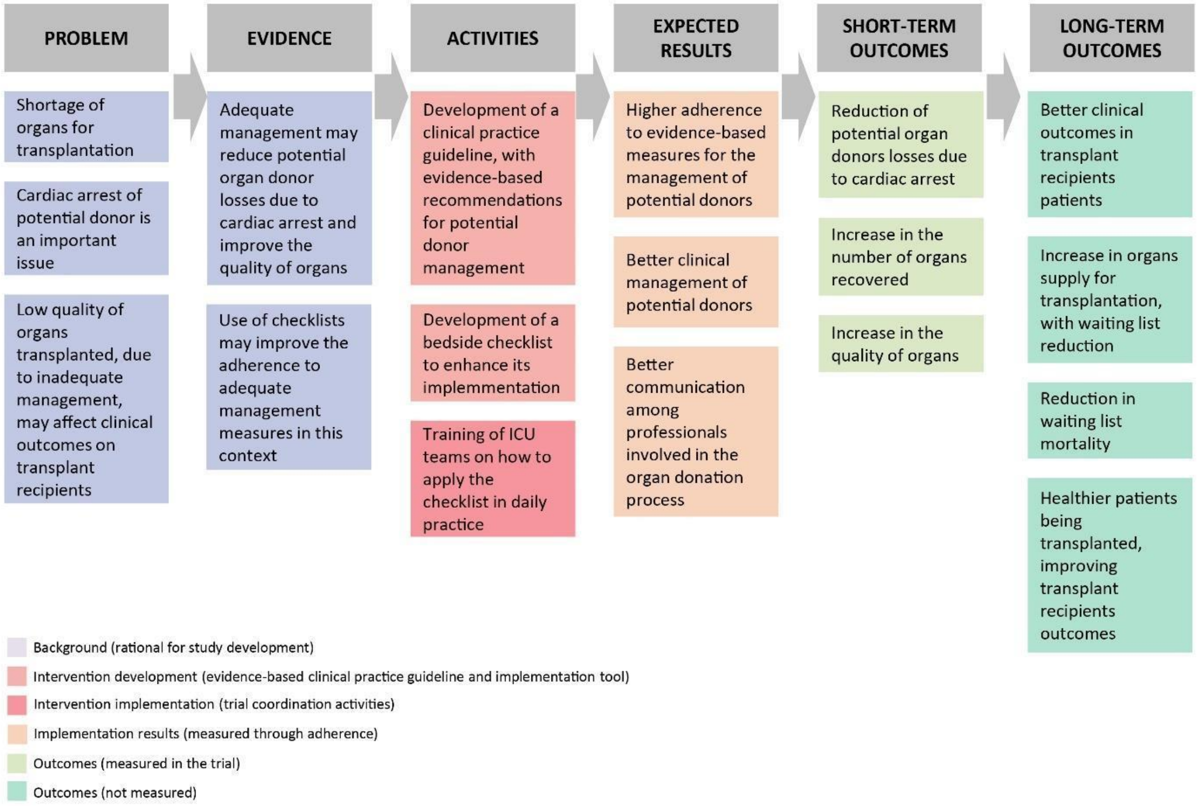

Adapted from study protocol: Westphal GA, Robinson CC, Biasi A, et al. DONORS (Donation Network to Optimise Organ Recovery Study): Study protocol to evaluate the implementation of an evidence-based checklist for brain-dead potential organ donor management in intensive care units, a cluster randomised trial. BMJ Open. 2019;9(6):e028570.

© 2023 Westphal GA et al. *JAMA Network Open*

eFigure 2. Description of the adherence measurement

The following combination of goals and immediate action were used to measure adherence to the checklist to each potential donor:

GOALS TO BE ACHIEVED	STATUS			IMMEDIATE ACTIONS WHEN STATUS = "NO"	ACTION TAKEN?	
$SaO_2 \geq 90\%$? **1**	□ Yes	□ No	□ NA	Adjust FiO_2 and/or PEEP to $SaO_2 \geq 90\%$ **1**	□ Yes	□ No
Vt of 6 to 8 mL/kg of predicted weight? **2**	□ Yes	□ No	□ NA	Adjust Vt to 6 to 8 mL/kg **2**	□ Yes	□ No
PEEP ≥ 8 cm H_2O? **3**	□ Yes	□ No	□ NA	Adjust PEEP to ≥ 8 cm H_2O **3**	□ Yes	□ No
MAP ≥ 65 mmHg and good tissue perfusion after a crystalloid bolus? **4**	□ Yes	□ No	□ NA	Continue fluid infusion while there is volume responsiveness (ex.: ΔPp ≥ 13% / ΔMAP ≥ 8% / ΔSV ≥ 10% / CVP < 8 mmHg) **4**	□ Yes	□ No
MAP ≥ 65 mmHg and good tissue perfusion after volume adjustment?	□ Yes	□ No	□ NA	Maintain / initiate noradrenaline (dopamine if bradycardia)	□ Yes	□ No
Vasopressin and hydrocortisone were associated after maintaining / initiating noradrenaline / dopamine? **5**	□ Yes	□ No	□ NA	Add vasopressin (1 IU bolus + 0.5-2.4 IU / h) and **5**<br>Add hydrocortisone 100 mg 8/8 h **6**	□ Yes<br>□ Yes	□ No<br>□ No
Diuresis < 4 mL/kg/h? **6**	□ Yes	□ No	□ NA	Assess need for volume replacement<br>Maintain / initiate vasopressin or desmopressin (IV) **7**	□ Yes	□ No
Na^+ < 155 mEq/L? **7**	□ Yes	□ No	□ NA	Correct and order laboratory control in 6 h **8**	□ Yes	□ No
K^+ between 3.5 and 5.5 mEq/L? **8**	□ Yes	□ No	□ NA	Correct and order laboratory control in 6 h **9**	□ Yes	□ No
Mg^{++} > 1.6 mEq/L? **9**	□ Yes	□ No	□ NA	Correct and order laboratory control in 6 h **10**	□ Yes	□ No
Capillary glycaemia < 180 mg/dL? **10**	□ Yes	□ No	□ NA	Insulin IV to maintain glycaemia between 140 and 180 mg/dL **11**	□ Yes	□ No
Haemoglobin ≥ 7 g/dL? **11**	□ Yes	□ No	□ NA	Transfuse red blood cells to Hb ≥ 7g/dL **12**	□ Yes	□ No
Absence of infection? **12**	□ Yes	□ No	□ NA	Initiate / maintain antibiotic therapy **13**	□ Yes	□ No
Proper body temperature? **13**<br>- No vasopressor: Goal: 34-35°C (after clinical tests)<br>- With vasopressor: > 35°C	□ Yes	□ No	□ NA	Get 34 to 35°C if without vasopressor □ NA<br>Get > 35°C if with vasopressor □ NA **14**	□ Yes<br>□ Yes	□ No<br>□ No

1. **Goal 1** combines with Immediate action when status = 'no' **1**
2. **Goal 2** combines with Immediate action when status = 'no' **2**
3. **Goal 3** combines with Immediate action when status = 'no' **3**
4. **Goal 4** combines with Immediate action when status = 'no' **4**
5. **Goal 5** combines with Immediate action when status = 'no' **5**
6. **Goal 5** combines with Immediate action when status = 'no' **6**
7. **Goal 6** combines with Immediate action when status = 'no' 7
8. **Goal 7** combines with Immediate action when status = 'no' **8**
9. **Goal 8** combines with Immediate action when status = 'no' **9**
10. **Goal 9** combines with Immediate action when status = 'no' **10**
11. **Goal 10** combines with Immediate action when status = 'no' **11**
12. **Goal 11** combines with Immediate action when status = 'no' **12**
13. **Goal 12** combines with Immediate action when status = 'no' **13**
14. **Goal 13** combines with Immediate action when status = 'no' **14**

$$Total\ adherence = \frac{\sum \quad Sum\ of\ achieved\ goals\ (checked\ as\ 'yes')\ or\ Immediate\ action\ conducted\ when\ status = 'no' (checked\ as\ 'yes')}{14 * Sum\ of\ checklists\ expected\ to\ be\ applied\ according\ to\ the\ following\ period}$$

Example: For a potential donor followed by 20 hours, we expected checklist application at: 1) enrollment time; 2) 6 hours after enrollment; 3) 12 hours after enrollment.

$$Total\ adherence = \frac{Enrollment\ time\ [\sum \quad] + \ 6\ hours\ after\ enrollment\ [\sum \quad] + 12\ hours\ after\ enrollmnet\ [\sum \quad]}{14 * 3}$$

Then, we have in the database:

Potential donor ID	site ID	Potential donor adherence (%)
1	1	xx.x
.	.	.
1.535	63	xx.x

To compute site adherence, we used descriptive measures (median e interquartile range) from the adherence of potential donors enrolled in the site.

© 2023 Westphal GA et al. *JAMA Network Open*

eMethods 1. Elaboration of the evidence-based clinical practice guideline for the management of potential brain-dead donors

As a preparatory phase for the trial, the study investigators convened a taskforce to elaborate an evidence-based clinical practice guideline for the management of potential brain-dead donors
The taskforce was composed by composed by the Coordination of the National Transplant System/Brazilian Ministry of Health, the Brazilian Association of Intensive Care Medicine, the Brazilian Association of Organ Transplantation, and the Brazilian Research in Intensive Care Network
This effort resulted in the current Brazilian guidelines for the management of brain-dead potential organ donors.[25]
The guidelines were developed using GRADE Methodology[26] in accordance with standards of the Guidelines International Network and the U.S. Institute of Medicine (IOM).[27]
The outcomes considered for decision-making were cardiac arrest, number of organs recovered per donor, and graft function/survival.
A total of 19 recommendations were drawn from the expert panel, including mechanical ventilation, vasoactive support, hormonal supplementation, electrolyte control, body temperature control, and the administration of antibiotics and blood products
The recommendations of the guideline served as the basis for the evidence-based clinical practice checklist tested in the study.

© 2023 Westphal GA et al. *JAMA Network Open*

eTable 2. Family interview support guide based on the Spanish model of communication in critical situations

PREPARING FOR THE FAMILY INTERVIEW	
**GROUNDS: Establishing an aid relationship with family members**<br>**Triad: Respect, Empathy, and Authenticity**	
**READ THE ACTIONS BELOW CAREFULLY BEFORE EACH STEP OF THE FAMILY INTERVIEW**	
**1. Arranging the location of the interview**	□ Well-ventilated place or room<br>□ Restricted access (avoid interferences)<br>□ Enough space and chairs for all participants<br>□ No barriers between interviewer and interviewee (e.g., table, chairs, etc.)<br>□ Facial tissues and water are available<br>□ Phones are turned off
**2. Defining the interview participants**	□ ICU physician<br>□ Transplant co-ordinator and/or ICU nurse are present<br>□ 1st[a]/2nd[b] degree relatives or legally authorised representative[1,c]
**3. Reviewing the components of non-verbal communication**	□ Have all family members sitting down<br>□ Leave land-line phones off the hook and turn off mobile phones<br>□ Avoid crossing your arms or legs<br>□ Have a trustful look and a serene expression<br>□ Speak in a gentle voice<br>□ Speak in a fine cadence, use pauses<br>□ Tolerate periods of silence<br>□ Give full attention to what family members say, "Listen more and talk less"
**4. Reviewing the components of verbal communication**	□ Greet everyone and introduce yourself<br>□ Refer to the patient by his/her name<br>□ Find out what the family knows about the case<br>□ Ask family members what they want to know<br>□ Summarize previous clinical data<br>□ Use simple language, avoid unnecessary technical jargon<br>□ Make your message clear, keep it short<br>□ Acknowledge emotions and negative reactions Avoid expressions like "do not cry", "keep calm", "I know how you feel"

© 2023 Westphal GA et al. *JAMA Network Open*

PREPARING FOR THE FAMILY INTERVIEW	
**STEP 1 - FIRST FAMILY CONFERENCE**<br>**COMMUNICATING THE ESTABLISHMENT OF A BRAIN DEATH PROTOCOL – 1st clinical examination**	
**Key points of the first conference**	□ **The ICU physician is responsible for** communicating about the possibility of death<br>□ **Communicate the <u>possibility</u> of brain death to the family**<br>- **<u>DO NOT</u>** talk about donation<br>- Inform that **further tests** will be performed<br>□ **<u>Review</u>** and **<u>confirm</u>** that the family understands what a suspected death is and that further tests will be performed<br>□ **<u>Make sure</u>** the family knows how to reach you for questions
**STEP 2 - SECOND FAMILY CONFERENCE**<br>**COMMUNICATING THE BRAIN DEATH – after 2 clinical tests and neuro-imaging evidence**	
**Key points of the second conference**	□ **The ICU physician is responsible for** communicating about the confirmation of brain death<br>□ **Communicate the confirmation of brain death to the family**<br>- Preferably use the word ‘death’ instead of the expression ‘brain death’. (despite all efforts, unfortunately your loved one died...)<br>□ **<u>DO NOT</u>** talk about donation<br>□ **Wait silently** for the family’s reactions and needs<br>□ **<u>Review</u>** and **<u>confirm</u>** that the family understands that the patient is dead<br>**<u>Ask the family</u>** if they have any questions
**IMPORTANT:** “Proceed to STEP 3 only after making sure that the family understands the death”	

© 2023 Westphal GA et al. *JAMA Network Open*

PREPARING FOR THE FAMILY INTERVIEW	
**STEP 3 - THIRD FAMILY CONFERENCE**<br>**INTERVIEW FOR MULTI-ORGAN DONATION - after the family's understanding of the death**	
**Key points of the third conference**	**Person leading the interview:**<br>□ 1st option: IHTC/OPO member<br>□ 2nd option: ICU physician or nurse<br><br>**Aspects of the interview:**<br>□ Check whether the family **understands** the meaning of the diagnosis of brain death (understands that their loved one is dead)<br>□ Explain to the family that the death occurred under **circumstances that allow them to help** other people by means of organ donation<br>□ Ask the family if their loved one had expressed a wish in life to be an organ donor<br>□ Offer the family, in view of this special situation, the opportunity to discuss about the possibility of organ donation (it is optional)<br>□ **Make sure** the family knows how to reach you for questions
**STEP 4 - PLANNING THE APPROACH ACCORDING TO THE FAMILY'S DECISION**	
□ **FAMILY CONSENT FOR DONATION**<br>**- Obtain the Family Consent Form**, fully and correctly completed<br>- Complete the **"Death Certificate"**	□ **FAMILY REFUSAL FOR DONATION**<br><br>**- Evaluate the possibility of a rescue interview** for donation after family conflicts have been resolved<br>**- Consider withdrawing therapeutic support** "The physician is legally and ethically entitled to withdraw therapeutic support, including mechanical ventilation, and release the body to the family." ¶<br>- Complete the **"Death Certificate"**

PREPARING FOR THE FAMILY INTERVIEW	
**GROUNDS: Establishing an aid relationship with family members**<br>**Triad: Respect, Empathy, and Authenticity**	
**STEP 4 - PLANNING THE APPROACH ACCORDING TO THE FAMILY'S DECISION**	
**DEATH CERTIFICATE or FORENSIC MEDICAL EXAMINATION**<br>**ICU physician's responsibility**	
□ **NON-VIOLENT DEATH**<br><br>- Complete the **"Death Certificate"** including the **date and time of death** and the data of the **last examination performed** (2nd clinical examination) **or** neuro-imaging evidence.	□ **VIOLENT DEATH**<br><br>- Complete the **"Forensic Medical Examination Referral Form"** including the **date and time of death** and the data of the **last examination performed** (2nd clinical examination) **or** neuro-imaging evidence.<br>- Request the Forensic Medical Institute for **AUTHORISATION TO REMOVE ORGANS OR TISSUES**

Source: Westphal GA, Robinson CC, Biasi A, et al. DONORS (Donation Network to Optimise Organ Recovery Study): Study protocol to evaluate the implementation of an evidence-based checklist for brain-dead potential organ donor management in intensive care units, a cluster randomised trial. BMJ Open. 2019;9(6):e028570.
[a] 1st degree relatives: father, mother, children, full siblings.
[b] 2nd degree relatives: grandparents, grandchildren.
[1,c] Legally authorised representative: surrogate/ judicial (documented)
[1] Brazilian Federal Law No. 10211 of March 23, 2001.
[2] Brazilian Federal Board of Medicine – Resolution No. 1826 of December 6, 2007.

© 2023 Westphal GA et al. *JAMA Network Open*

eMethods 2. Exploratory outcomes according to the statistical analysis plan

**Exploratory outcomes according to the statistical analysis plan**
Proportion of potential donors with adequate respiratory parameters, defined as PaO_2/FiO_2 ratio ≥200. In the absence of simultaneously measured PaO_2 and FiO_2, adequate respiration was defined as SaO_2/FiO_2 ≥240 (if positive end-expiratory pressure [PEEP] <8 cm H_2O), ≥259 (if PEEP 8–12 cm H_2O), or ≥234 (if PEEP >12 cm H_2O)[1] (presented as risk ratio [RR] and 95% confidence interval [CI]).
Proportion of potential donors with adequate body temperature, defined as 34–35°C if hemodynamically stable and >35°C if mean arterial pressure (MAP) was <65 mm Hg or norepinephrine or dopamine was required (presented as RR and 95% CI).
Proportion of potential donors with adequate circulatory parameters, considering the following as inadequate: MAP <65 mm Hg or norepinephrine ≥0.1 mcg/kg/min or dopamine ≥15 mcg/kg/min (presented as RR and 95% CI).
Sequential Organ Failure Assessment (SOFA) score, as per Vincent et al. (1996)[2] (presented as mean difference [MD] and 95% CI).
Proportion of potential donors receiving lung-protective ventilation: tidal volume (Vt) 6–8 mL/kg of predicted body weight and PEEP ≥8 cm H_2O (presented as RR and 95% CI).
Proportion of potential donors receiving vasopressin if on norepinephrine or dopamine (presented as RR and 95% CI).
Proportion of potential donors receiving hydrocortisone if on norepinephrine or dopamine (presented as RR and 95% CI).
Proportion of potential donors with Na^+ <155 mEq/L (presented as RR and 95% CI).
Proportion of potential donors with Mg^{++} >1.6 mEq/L (presented as RR and 95% CI).
Proportion of potential donors with K^+ 3.5 to 5.5 mEq/L (presented as RR and 95% CI).
Proportion of potential donors with capillary blood glucose <180 mg/dL (presented as RR and 95% CI).
Proportion of potential donors receiving antibiotics, among those with infection (presented as RR and 95% CI).

Source: Giordani NE, Robinson CC, Westphal GA, et al. Statistical analysis plan for a cluster-randomised trial assessing the effectiveness of implementation of a bedside evidence-based checklist for clinical management of brain-dead potential organ donors in intensive care units: DONORS (Donation Network to Optimise Organ Recovery Study). Trials. 2020;21(1):540.

[1] Pandharipande PP, Shitani AK, Hagerman HE, St Jacques PJ, Rice TW, Sanders NW, et al. Derivation and validation of Spo2/Fio2 ratio to impute for Pao2/Fio2 ratio in the respiratory component of the Sequential Organ Failure Assessment score. Crit Care Med. 2009;37(4):1317–21.21.

[2] Vincent JL, Moreno R, Takala J, Willatts S, De Mendonça A, Bruining H, et al. The SOFA (Sepsis-related Failure Assessment) score to describe organ disfunction/failure. Intensive Care Med. 1996;22:707–10.

© 2023 Westphal GA et al. *JAMA Network Open*

eTable 3. Post hoc analyses and reasons for their selection

Analysis	Requested by	Reason/Objective
Characteristics of the sites and the brain-dead potential organ donors at baseline considering site adherence to the intervention	Independent statistical board	To present the characteristics of sites and potential donors as a function of adherence at the site level, allowing to assess comparability between groups (selection bias).
Adherence to each checklist item (at the brain-dead potential organ donor level)	Steering committee	To describe adherence to each item individually in order to provide greater transparency about adherence to each item of the checklist at the potential donor level.
Adherence to the intervention per site	Steering committee	To describe adherence per site individually in order to provide greater transparency about adherence at the site level.
Post hoc analysis for the primary outcome combining site and brain-dead potential organ donor adherence to the intervention	Steering committee	To check the consistency of adherence to the checklist at the potential donor level stratified by adherence at the site level in order to investigate potential confounders or the occurrence of reverse causality.
Characteristics of the brain-dead potential organ donors at baseline considering adherence to the intervention at the potential organ donor level	Steering committee	To present the characteristics of sites and potential donors as a function of adherence at the potential organ donor, allowing to assess comparability between groups (selection bias).
Descriptive analysis for a proxy for site quality	Steering committee	To compare the sites with the highest and lowest adherence to the checklist, descriptively, using proxies for site quality based on study data (time from inclusion in the study to organ harvesting, and time from inclusion in the study to the primary outcome) and based on data recorded in 2016 in the Brazilian National Transplant System (donation and cardiac arrest rates), allowing to assess comparability between groups and to investigate selection bias and confounding bias.
Post hoc analysis by proxy for site quality using a directed acyclic graph (DAG)	Steering committee	To check the consistency of adherence to the checklist using proxies for site quality based on the 2016 data from the Brazilian National Transplant System (donation and cardiac arrest rates) in order to investigate potential confounders or the occurrence of reverse causality.

© 2023 Westphal GA et al. *JAMA Network Open*

Analysis	Requested by	Reason/Objective
Post hoc analysis adjusted for the characteristics of sites and brain-dead potential organ donors considering site adherence to the intervention and using a directed acyclic graph (DAG)	Independent statistical board	To check the consistency of adherence to the checklist at the site level, adjusting the primary outcome for potential confounders of effects.
Post hoc analysis adjusted for the characteristics of brain-dead potential organ donors considering their adherence to the intervention and using a directed acyclic graph (DAG)	Steering committee	To check the consistency of adherence to the checklist at the potential donor level, adjusting the primary outcome for potential confounders of effects.
Post hoc ancillary analysis of the primary outcome according to adherence to the intervention at the potential donor level by quintiles of adherence	Steering committee	To describe the primary outcome rate according to quintiles of adherence at the potential donor level in order to investigate a dose-response gradient.
Post hoc analysis considering adherence to the intervention according to the order of brain-dead potential donor inclusion	Steering committee	To describe adherence at the potential donor level according to the order of inclusion in the study (every 10 inclusions).
Post hoc analysis considering adherence to the intervention according to the time of brain-dead potential donor inclusion	Steering committee	To check the consistency of adherence to the checklist at the potential donor level, adjusting the primary outcome for potential confounders of effects related to the site's learning curve for using the checklist.
Post hoc analysis of the secondary outcomes considering adherence to the intervention in the potential organ donor level	Steering committee	To check the consistency of secondary regarding adherence to the intervention.
Post hoc analysis of the secondary outcomes considering adherence to the intervention in the site level	Steering committee	To check the consistency of secondary regarding adherence to the intervention.
Descriptive analysis	Steering committee	Rate of actual donors per site

eFigure 3. Geographic distribution of the included sites

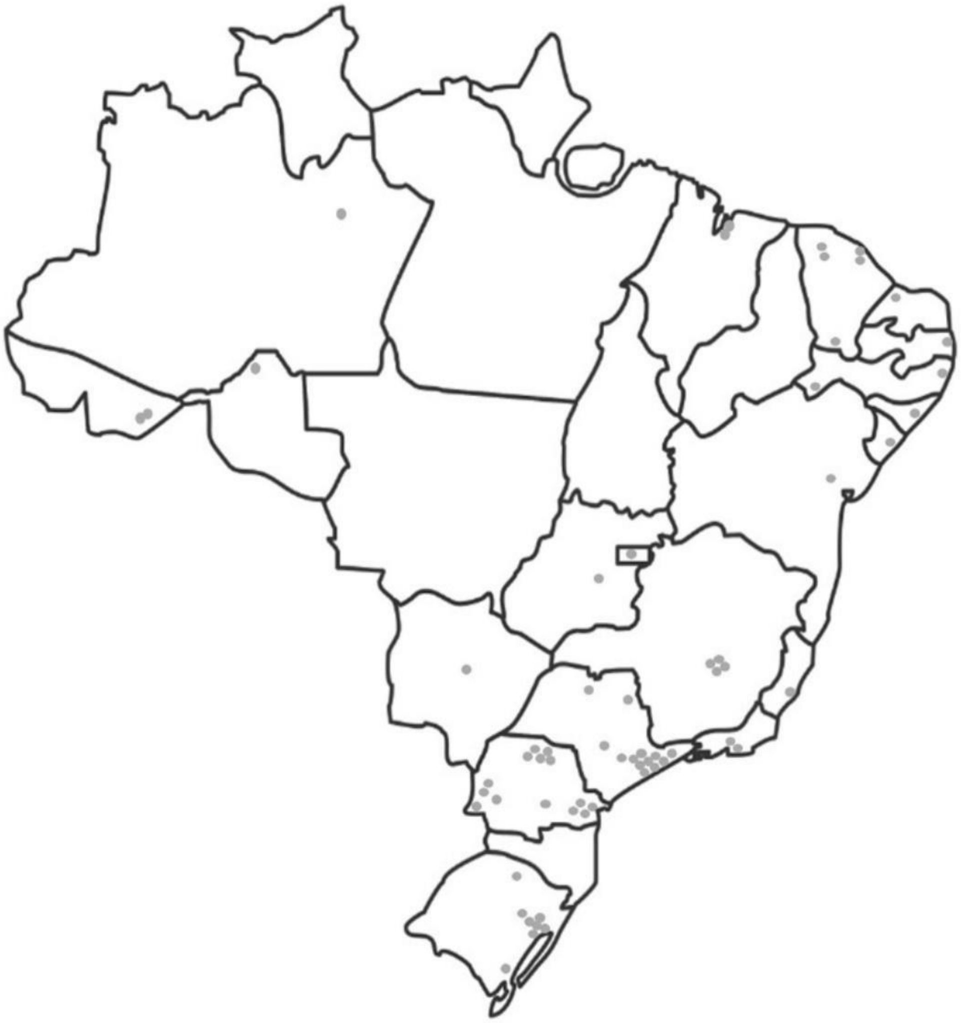

Source: Westphal GA, Robinson CC, Biasi A, et al. DONORS (Donation Network to Optimize Organ Recovery Study): Study protocol to evaluate the implementation of an evidence-based checklist for brain-dead potential organ donor management in intensive care units, a cluster randomized trial. BMJ Open. 2019;9(6):e028570.

eTable 4. Post hoc analysis considering adherence to the intervention according to the time of brain-dead potential donor inclusion

Analysis	Intervention arm n/total (%)	Control arm n/total (%)	RR (95% CI)	P value
Potential organ donors lost due to cardiac arrest adjusted for time since first inclusion, 187/1535 (12.2%)	70/743 (9.4)	117/792 (14.8)	0.70 (0.46 to 1.08)	0.108
Potential organ donors lost due to cardiac arrest adjusted for order of inclusion, 187/1535 (12.2%)	70/743 (9.4)	117/792 (14.8)	0.70 (0.46 to 1.08)	0.105
Time from inclusion to cardiac arrest in hours, median (IQR)	26 (11.8 to 54.5)	32.3 (15.1 to 71.3)	-	0.19

Abbreviations: HR, hazard ratio; CI, confidence interval; IQR, interquartile range, Time since first inclusion is defined as the time difference between potential inclusion and first inclusion per site; Inclusion order is defined as the order of inclusion of the potential donor per site.

© 2023 Westphal GA et al. *JAMA Network Open*

eFigure 4. Subgroup analysis of the primary outcome (loss of brain-dead potential organ donors due to cardiac arrest)

CI, confidence interval; ICU, intensive care unit; SAPS, Simplified Acute Physiology Score.

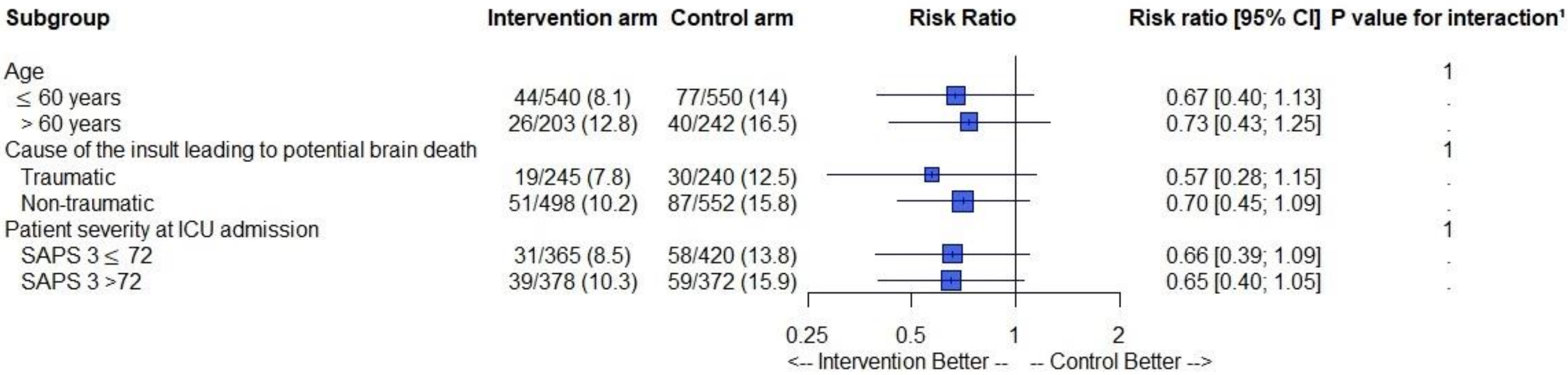

© 2023 Westphal GA et al. *JAMA Network Open*

eTable 5. Sensitivity analysis for the primary outcome

Analysis	n/N (%)	n/N (%)	RR (95% CI)	P value	P value for interaction
**1. Adherence to the intervention**					
Intervention arm – adherence >79.0% vs adherence ≤79.0%	19/361 (5.3)	51/382 (13.4)	0.42 (0.25 to 0.73)	0.0021	
Intervention arm – adherence ≤79.0% vs Control arm	51/382 (13.4)	117/792 (14.8)	0.93 (0.1 to 1.42)	0.75	
Intervention arm – adherence > 79.0% vs Control arm.	19/361 (5.3)	117/792 (14.8)	0.41 (0.22 to 0.78)	0.0059	
	**Intervention arm**	**Control arm**			
**2. Time elapsed between first clinical examination consistent with brain death and inclusion in the study**					
Time elapsed between first clinical examination consistent with brain death and inclusion in the study = 0	31/349 (8.9)	83/513 (16.2)	0.63 (0.37 to 1.09)	0.101	0.90
Time elapsed between first clinical examination consistent with brain death and inclusion in the study > 0	39/394 (9.9)	34/279 (12.2)	0.88 (0.45 to 1.73)	0.71	
**3. Failures in the screening of consecutive participants (N=1000 simulations)**	102/958 (10.6)	129/873 (14.8)	0.78 (0.42 to 1.14)	0.331	
**4. Estimated number of brain death notifications in each ICU (≤29 vs. >29, according to the stratification variable)**					
Estimated number of brain death notifications in each ICU ≤29	41/436 (9.4)	58/462 (12.6)	0.84 (0.47 to 1.49)	0.55	0.30
Estimated number of brain death notifications in each ICU >29	29/307 (9.4)	59/330 (17.9)	0.54 (0.29 to 0.99)	0.047	
**5. Donation rate for each site before the study**					
Donation rate for each site before the study ≤29.0%	46/380 (12.1)	66/393 (16.8)	0.79 (0.47 to 1.31)	0.36	0.46
Donation rate for each site before the study >29.0%	24/363 (6.6)	51/399 (12.8)	0.58 (0.29 to 1.17)	0.13	

Abbreviations: P value for interaction does not apply to analyses 1 and 3; ICU, intensive care unit; CI, confidence interval; HR, hazard ratio.

© 2023 Westphal GA et al. *JAMA Network Open*

eTable 6. Sensitivity analysis of secondary outcomes regarding adherence considering potential organ donor adherence to the intervention

Group	Descriptive measures<br>Actual donors (%)<br>Organs recovered (Mean)	Actual donor		Organs recovered
		RR	RD	MD
Adherence ≤ 79% vs. Control Arm	38.5% vs. 41.2%<br>2.9 vs. 2.8	0.96 (0.77 to 1.18)<br>P = 0.68	-1.79 (-10.2 to 6.60)<br>P = 0.68	0.09 (-0.11 to 0.30)<br>P = 0.37
Adherence > 79% vs. Control Arm	49.9% vs. 41.2%<br>2.7 vs. 2.8	1.13 (0.93 to 1.39)<br>P = 0.23	5.47 (-3.49 to 14.40)<br>P = 0.23	0.01 (-0.25 to 0.28)<br>P = 0.93
Adherence > 79% vs. Adherence ≤ 79%	49.9% vs. 38.5%<br>2.7 vs. 2.9	1.11 (0.91 to 1.36)<br>P = 0.31	4.44 (-4.21to 13.1)<br>P = 0.31	-0.06 (-0.35 to 0.23)<br>P = 0.69

Abbreviations: RR, risk ratio; RD, risk difference; MD, mean difference; P.

© 2023 Westphal GA et al. *JAMA Network Open*

eTable 7. Characteristics of the brain-dead potential organ donors at baseline considering adherence to the intervention at the potential organ donor level

Characteristic	Control arm (N=792)	Intervention arm		
		Overall (N=743)	Adherence >79% (N=361)	Adherence ≤79% (N=382)
Age in years – median (IQR)	51.5 (36.8 - 62.9)	50.8 (35.8 - 61.2)	51.9 (37.9 - 62.3)	49.35 (34.5 - 60.4)
Age > 60 years – no. (%)	242 (30.6)	203 (27.3)	104 (28.8)	99 (25.9)
Female sex – no. (%)	314 (39.6)	312 (42)	165 (45.7)	147 (38.5)
SOFA score at enrollment – median (IQR)	10 (9 - 12)	11 (9 - 13)	11 (9 - 13)	11 (9 - 13)
SAPS 3 score at ICU admission – median (IQR)	72 (63 - 80)	73 (64 - 80)	72 (63 - 80)	73 (65 - 80)
Comorbidities				
Diabetes mellitus – no. (%)	89 (11.2)	96 (12.9)	44 (12.2)	52 (13.6)
Hypertension – no. (%)	314 (39.6)	311 (41.9)	154 (42.7)	157 (41.1)
Renal failure requiring dialysis – no. (%)	20 (2.5)	16 (2.2)	7 (1.9)	9 (2.4)
Chronic respiratory disease – no. (%)	11 (1.4)	14 (1.9)	5 (1.4)	9 (2.4)
Heart failure – no. (%)	21 (2.7)	17 (2.3)	12 (3.3)	5 (1.3)
Chronic liver disease – no. (%)	2 (0.3)	2 (0.3)	2 (0.6)	0 (0.0)
Cause of brain injury				
Trauma – no. (%)	240 (30.3)	245 (33.0)	113 (31.3)	132 (34.6)
Stroke – no. (%)	468 (59.1)	409 (55.0)	210 (58.2)	199 (52.1)
Anoxia – no. (%)	52 (6.6)	56 (7.5)	26 (7.2)	30 (7.9)
Other – no. (%)	32 (4.0)	33 (4.4)	12 (3.3)	21 (5.5)
Use of antimicrobial medication[a] – no. (%)	500 (63.1)	467 (62.9)	221 (61.2)	246 (64.4)
Length of hospital stay in days before brain death diagnosis – median (IQR)	4.11 (1.9 - 7.9)	4.3 (1.8 - 8.5)	4.4 (1.7 - 8.4)	4.11 (2.1 - 8.6)

Abbreviations: IQR, interquartile range; SOFA, Sequential Organ Failure Assessment; SAPS, Simplified Acute Physiology Score.
[a] Identified at the time of first clinical examination.

© 2023 Westphal GA et al. *JAMA Network Open*

eTable 8. Characteristics of the sites and the brain-dead potential organ donors at baseline considering site adherence to the intervention at the site level

Characteristic	Control arm	Intervention arm		
		Overall	Adherence ≤77.5%	Adherence >77.5%
**Included sites (N=63)**	**N=32**	**N=31**	**N=16**	**N=15**
Number of hospital beds – median (IQR)	248.5 (198.5 - 469.2)	282.0 (193.0 - 607.0)	292.5 (196.0 - 501.0)	216.0 (197.5 - 816.0)
Number of ICU beds – median (IQR)	39.0 (28.0 - 68.0)	50.0 (29.5 - 68.5)	52.0 (31.8 - 61.8)	42.0 (29.5 - 101.0)
Number of ICU beds/hospital beds – median (IQR)	14.1 (12.1 - 18.1)	13.2 (10.7 - 17.9)	12.9 (10.7 - 18.4)	13.7 (10.9 - 16.1)
Number of adult ICU beds – median (IQR)	26.0 (20.0 - 47.2)	34.0 (24.0 - 50.5)	34.0 (25.5 - 40.2)	42.0 (22.0 - 68.5)
ICU type				
Surgical – no. (%)	0 (0)	1 (3)	0 (0)	1 (7)
Mixed – no. (%)	32 (100)	30 (97)	16 (100)	14 (93)
Hospital type				
Public – no. (%)	17 (53)	17 (55)	10 (63)	7 (47)
Private – no. (%)	15 (47)	14 (45)	6 (38)	8 (53)
Teaching activity – no. (%)	26 (81)	23 (74)	11 (69)	12 (80)
Transplant center – no. (%)	15 (47)	10 (32)	5 (31)	5 (33)
Number of annual brain death notifications[a] – median (IQR)	22.0 (16.1 - 37.2)	24.0 (16.0 - 34.5)	22.5 (14.8 - 40.5)	24.0 (21.0 - 29.5)
**Included brain-dead potential organ donors (N=1535)**	**N=792**	**N=743**	**N=351**	**N=392**
Age in years – median (IQR)	51.5 (36.8 - 62.9)	50.8 (35.8 - 61.2)	47.5 (32.4 - 60.9)	51.8 (40.2 - 61.4)
Age > 60 years – no. (%)	242 (30.6)	203 (27.3)	96 (27.4)	107 (27.3)
Female sex – no. (%)	314 (39.6)	312 (42.0)	140 (39.9)	172 (43.9)
SOFA score at enrollment – median (IQR)	10.0 (9.0 - 12.0)	11.0 (9.0 - 13.0)	11.0 (9.0 - 13.0)	11.0 (9.0 - 12.0)
SAPS 3 score at ICU admission – median (IQR)	72.0 (63.0 - 80.0)	73.0 (64.0 - 80.0)	72.0 (63.0 - 80.0)	73.0 (65.0 - 81.0)
Comorbidities				

Diabetes mellitus – no. (%)	89 (11.2)	96 (12.9)	46 (13.1)	50 (12.8)
Hypertension – no. (%)	314 (39.6)	311 (41.9)	133 (37.9)	178 (45.4)
Renal failure requiring dialysis – no. (%)	20 (2.5)	16 (2.2)	8 (2.3)	8 (2.0)
Chronic respiratory disease – no. (%)	11 (1.4)	14 (1.9)	7 (2.0)	7 (1.8)
Heart failure – no. (%)	21 (2.7)	17 (2.3)	5 (1.4)	12 (3.1)
Chronic liver disease – no. (%)	2 (0.3)	2 (0.3)	0 (0.0)	2 (0.5)
Cause of brain injury				
Trauma – no. (%)	240 (30.3)	245 (33.0)	134 (38.2)	111 (28.3)
Stroke – no. (%)	468 (59.1)	409 (55.0)	167 (47.6)	242 (61.7)
Anoxia – no. (%)	52 (6.6)	56 (7.5)	27 (7.7)	29 (7.4)
Other – no. (%)	32 (4.0)	33 (4.4)	23 (6.6)	10 (2.6)
Use of antimicrobial medication[b] – no. (%)	500 (63.1)	467 (62.9)	229 (65.2)	238 (60.7)
Length of hospital stay in days before brain death diagnosis – median (IQR)	4.1 (1.9 - 7.9)	4.3 (1.8 - 8.5)	4.7 (2.1 - 9.1)	4.0 (1.7 - 7.8)

Intervention sites were grouped according to the global median adherence of the intervention sites.

Abbreviations: ICU, intensive care unit; IQR, interquartile range; SAPS, Simplified Acute Physiology Score; SOFA, Sequential Organ Failure Assessment; Chronic respiratory disease is defined as restrictive, obstructive, or vascular disease severe enough to limit performance of the activities of daily living or chronic hypoxia, hypercapnia, polycythemia, pulmonary hypertension, or ventilator dependence; Chronic liver disease is defined as biopsy-proven cirrhosis or proven portal hypertension or previous history of hepatic insufficiency, encephalopathy, or coma.

[a] Number of annual brain death notifications considers the percentage of brain-dead potential organ donors clinically managed in the intensive care unit.

[b] Identified at the time of first clinical examination.

© 2023 Westphal GA et al. *JAMA Network Open*

eTable 9. Post hoc analysis of secondary outcomes regarding adherence considering site adherence to the intervention

Group	Descriptive measures<br>Actual donors (%)<br>Organs recovered<br>(Mean)	Actual donor		Organs recovered
		RR	RD	MD
Adherence ≤ 77.5% vs. Control Arm	35.5% vs. 41.2%<br>2.9 vs. 2.8	0.84 (0.68 to 1.05)<br>P = 0.12	-6.49 (-14.5 to 1.55)<br>P = 0.11	0.11 (-0.07 to 0.30)<br>P = 0.22
Adherence > 77.5% vs. Control Arm	51.8% vs. 41.2%<br>2.7 vs. 2.8	1.25 (1.03 to 1.52)<br>P = 0.026	10.30 (0.98 to 19.5)<br>P = 0.030	-0.01 (-0.30 to 0.27)<br>P = 0.93
Adherence > 77.5% vs. Adherence ≤ 77.5%	51.8% vs. 35.3%<br>2.7 vs. 2.9	1.48 (1.18 to 1.86)<br>P = 0.0007	16.80 (7.07 to 26.50)<br>P = 0.0007	-0.01 (-0.26 to 0.23)<br>P = 0.91

Abbreviations: RR, risk ratio; RD, risk difference; MD, mean difference; P.

© 2023 Westphal GA et al. *JAMA Network Open*

eTable 10. Post hoc analysis for the primary outcome combining site and brain-dead potential organ donor adherence to the intervention

Analysis	Adherence group	Intervention arm n/total (%)	Control arm n/total (%)	Effect estimate (95% CI)	P value
Potential organ donors lost due to cardiac arrest, 129/1076 (12.0%)	Site >77.5%<br>Potential donor >79%	12/284 (4.2)	117/792 (14.8)	RR 0.31 (0.16 to 0.61)	0.0007
Potential organ donors lost due to cardiac arrest, 132/900 (14.7%)	Site >77.5%<br>Potential donor ≤79%	15/108 (13.9)	117/792 (14.8)	RR 0.99 (0.55 to 1.81)	0.99
Potential organ donors lost due to cardiac arrest, 124/869 (14.3%)	Site ≤77.5%<br>Potential donor >79%	7/77 (9.1)	117/792 (14.8)	RR 0.64 (0.25 to 1.60)	0.34
Potential organ donors lost due to cardiac arrest, 153/1066 (14.4%)	Site ≤77.5%<br>Potential donor ≤79%	36/274 (13.1)	117/792 (14.8)	RR 0.90 (0.57 to 1.44)	0.67

Abbreviations: CI, confidence interval; HR, hazard ratio.

© 2023 Westphal GA et al. *JAMA Network Open*

eTable 11. Comparison of physiologic and treatment goals at baseline between the intervention and control groups and between high (>77.5%) and low adherence (≤ 77.5%) centers

	Intervention group Baseline	Control group Baseline	p value
Proportion of potential donors with adequate respiratory parameters.	510/743 (68.6%)	549/792 (69.3%)	0.77
Proportion of potential donors receiving protective ventilation.	260/743 (34.9%)	115/792 (14.5%)	<0.001
Proportion of potential donors with adequate circulatory parameters.	234/743 (31.5%)	253/792 (31.9%)	0.85
Proportion of potential donors receiving hydrocortisone if on noradrenaline or dopamine.	341/743 (45.8%)	323/792 (40.7%)	0.04
Proportion of potential donors receiving vasopressin if on noradrenaline or dopamine.	336/743 (45.2%)	214/792 (27.0%)	<0.001
Proportion of potential donors with Na <155 mEq/L.	520/743 (69.9%)	506/792 (63.8%)	0.01
Proportion of potential donors with Mg > 1.6 mEq/L.	403/743 (54.2%)	357/792 (45.1%)	<0.001
Proportion of potential donors with K 3.5–5.5 mEq/L.	496/743 (66.7%)	515/792 (65.0%)	0.47
Proportion of potential donors with capillary blood glucose <180 mg/dL.	421/743 (56.6%)	424/792 (53.5%)	0.22
	**Intervention group Adherence >77.5%**	**Intervention group Adherence ≤77.5%**	**p value**
Proportion of potential donors with adequate respiratory parameters.	256/392 (65.31%)	170/351 (48.43%)	<0.001
Proportion of potential donors receiving protective ventilation.	335/392 (85.46%)	264/351 (75.21%)	0.004
Proportion of potential donors with adequate circulatory parameters.	304/392 (77.55%)	253/351 (72.08%)	0.09
Proportion of potential donors receiving hydrocortisone if on noradrenaline or dopamine.	189/392 (48.21%)	173/351 (49.29%)	1.00
Proportion of potential donors receiving vasopressin if on noradrenaline or dopamine.	189/392 (48.21%)	173/351 (49.29%)	0.77
Proportion of potential donors with Na <155 mEq/L.	275/392 (70.15%)	215/351 (61.25%)	0.01
Proportion of potential donors with Mg > 1.6 mEq/L.	233/392 (59.44%)	180/351 (51.28%)	0.02
Proportion of potential donors with K 3.5–5.5 mEq/L.	272/392 (69.39%)	222/351 (63.25%)	0.08
Proportion of potential donors with capillary blood glucose <180 mg/dL.	267/392 (68.11%)	217/351 (61.82%)	0.07

© 2023 Westphal GA et al. *JAMA Network Open*

eTable 12. Comparison of goal achievement between the intervention and control groups

Outcome	Intervention arm	Control arm	Effect estimate (95% CI)
1. Potential donors with adequate respiratory parameters - % (n/N)	63.4 (336/530)	63.0 (423/672)	RR 1.04 (0.94 to 1.15)
2. Potential donors with adequate body temperature - % (n/N)	66.1 (388/587)	65.4 (496/758)	RR 1.00 (0.92 to 1.09)
3. Potential donors with adequate circulatory parameters - % (n/N)	52.8 (317/601)	44.7 (340/761)	RR 1.20 (1.04 to 1.38)
4. Potential donors receiving lung-protective ventilation - % (n/N)	56.7 (314/554)	20.8 (141/676)	RR 2.65 (2.16 to 3.26)
5. Potential donors receiving vasopressin if on norepinephrine or dopamine - % (n/N)	45.3 (272/601)	23.6 (180/763)	RR 1.82 (1.53 to 2.17)
6. Potential donors receiving hydrocortisone if on norepinephrine or dopamine - % (n/N)	65.2 (322/494)	71.2 (343/482)	RR 0.94 (0.85 to 1.03)
7. Potential donors with Na^+ <155 mEq/L - % (n/N)	66.2 (312/471)	56.6 (333/588)	RR 1.15 (1.02 to 1.29)
8. Potential donors with Mg^{++} >1.6 mEq/L - % (n/N)	87.3 (275/315)	91.6 (295/322)	RR 0.95 (0.89 to 1.01)
9. Potential donors with K^+ 3.5–5.5 mEq/L - % (n/N)	69.7 (325/466)	66.8 (396/593)	RR 1.04 (0.95 to 1.13)
10. Potential donors with capillary blood glucose <180 mg/dL - % (n/N)	63.7 (300/471)	58.4 (330/565)	RR 1.08 (0.96 to 1.22)
11. Potential donors receiving antibiotics (among those with infection) - % (n/N)	97.9 (373/381)	99.0 (390/394)	RR 0.99 (0.97 to 1.01)

Abbreviations: CI, confidence interval; SD, standard deviation; MD, mean difference; RR, risk ratio.
Note: The exploratory analysis considered the correlation between repeated measures within-subject adjusted for site; For exploratory outcomes 2 to 12, the denominator (N) is the number of potential donors with at least the data of one time point available.

© 2023 Westphal GA et al. *JAMA Network Open*

eTable 13. Individual adherence to the goals over time in the intervention group comparing participants whose physiology met parameters at the baseline to those who did not meet parameters

Goals met at baseline	6 hours	12 hours	24 hours	48 hours	72 hours
Protective ventilation - Met	501/566 (88.5%)	424/493 (86%)	262/330 (79.3%)	78/105 (74.2%)	26/44 (59.1%)
Protective ventilation - Not met	59/124 (47.5%)	65/106 (61.3%)	43/75 (57.3%)	18/32 (56.2%)	10/17 (58.8%)
Sodium < 155 mEq/L - Met	326/459 (71.0%)	256/391 (65.4%)	148/265 (55.8%)	47/84 (55.9%)	18/36 (50%)
Sodium < 155 mEq/L - Not met	34/231 (14.7%)	51/208 (24.5%)	41/140 (29.2%)	16/53 (30.1%)	10/25 (40%)
Magnesium > 1,6 mEq/L - Met	239/393 (60.8%)	210/341 (61.5%)	134/223 (60.1%)	45/73 (61.6%)	20/30 (66.6%)
Magnesium > 1,6 mEq/L - Not met	89/297 (29.9%)	71/258 (27.5%)	58/182 (31.8%)	14/64 (21.8%)	1/31 (3.2%)
Potassium 3.5-5.5 mEq/L - Met	319/468 (68.1%)	262/405 (64.6%)	157/276 (56.8%)	50/99 (50.5%)	22/45 (48.9%)
Potassium 3.5-5.5 mEq/L - Not met	74/222 (33.3%)	88/194 (45.3%)	54/129 (41.8%)	12/38 (31.5%)	4/16 (25.0%)
Glycaemia < 180 mg/dL - Met	319/449 (71.0%)	240/391 (61.3%)	144/253 (56.9%)	48/91 (52.7%)	27/46 (58.7%)
Glycaemia < 180 mg/dL - Not met	71/241 (29.4%)	73/208 (35.1%)	40/152 (26.3%)	14/46 (30.4%)	3/15 (20%)
Adequate hemodynamics - Met	311/526 (59.1%)	266/460 (57.8%)	175/318 (55.0%)	47/110 (42.7%)	20/47 (42.5%)
Adequate hemodynamics - Not met	52/164 (31.7%)	49/139 (35.2%)	33/87 (37.9%)	6/27 (22.2%)	3/14 (21.4%)
Vasopressin - Met	293/346 (84.6%)	251/305 (82.3%)	167/215 (77.6%)	54/75 (72%)	19/37 (51.3%)
Vasopressin - Not met	76/344 (22.1%)	67/294 (22.7%)	49/190 (25.7%)	17/62 (27.4%)	4/24 (16.7%)
Hydrocortisone - Met	296/346 (85.5%)	256/305 (83.9%)	168/215 (78.1%)	55/75 (73.3%)	20/37 (54.0%)
Hydrocortisone - Not met	83/344 (24.1%)	71/294 (24.1%)	53/190 (27.89%)	20/62 (32.2%)	6/24 (25.0%)

© 2023 Westphal GA et al. *JAMA Network Open*

eFigure 5. Correlation between adherence to the intervention per site and at individual level (potential donor adherence)

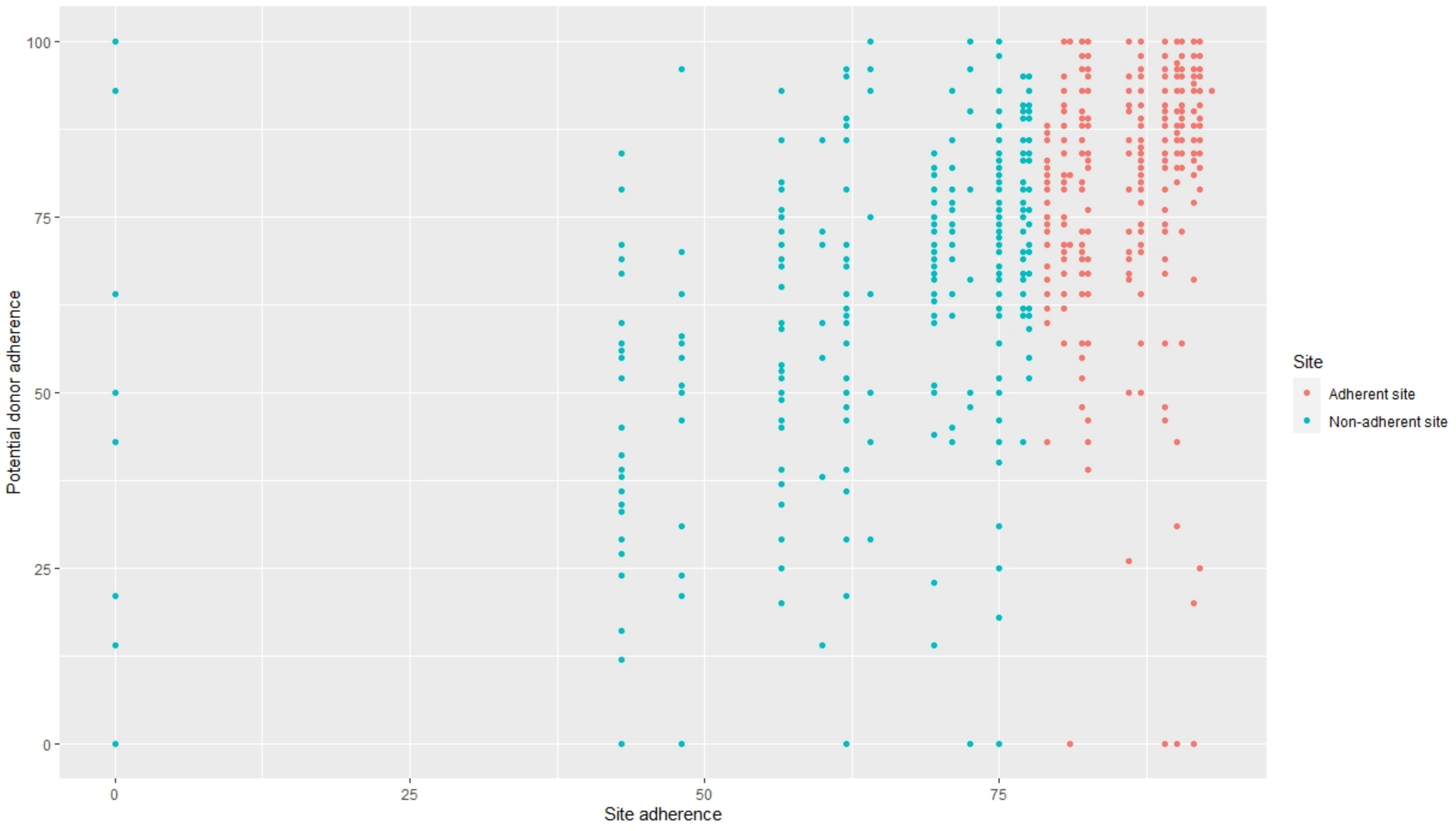

© 2023 Westphal GA et al. *JAMA Network Open*

eTable 14. Descriptive analysis for a proxy for site quality

Proxy	Adherent site (>77.5%) (N=15)	Non-adherent site (≤77.5%) (N=16)	P value[a]
Donation rate in 2016 (%) – median (IQR)	34.3 (20.3 - 42.5)	25.7 (21.9 - 32.0)	0.51
Cardiac arrest rate in 2016 (%) – median (IQR)	13.0 (8.1 - 16.0)	12.6 (7.4 - 22.6)	0.62
Time to organ harvesting (hours) – median (IQR)	29.6 (22.6 - 34.7)	28.1 (26.2 - 47.3)	0.34
Time to event (hours) – median (IQR)	25.9 (22.0 - 31.7)	25.6 (21.3 - 34.0)	0.91

Abbreviations: IQR, interquartile range.
[a] Wilcox on test.

© 2023 Westphal GA et al. *JAMA Network Open*

eTable 15. Post hoc analysis by proxy for site quality using a directed acyclic graph (DAG)

Proxy	Effect estimate (95% CI)	P value
–	RR 0.70 (0.46 to 1.08)	0.108
Donation rate in 2016	RR 0.71 (0.46 to 1.09)	0.12
Cardiac arrest rate in 2016	RR 0.69 (0.47 to 1.02)	0.060

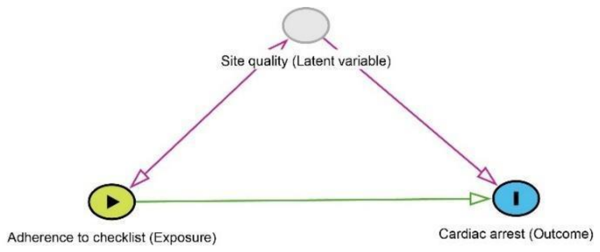

Abbreviations: CI, confidence interval; HR, hazard ratio.

© 2023 Westphal GA et al. *JAMA Network Open*

eTable 16. Post hoc analysis adjusted for the characteristics of sites and brain-dead potential organ donors considering site adherence to the intervention and using a directed acyclic graph (DAG)

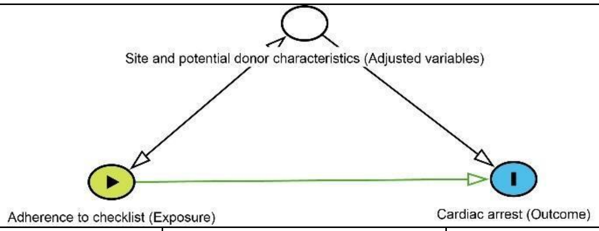

Analysis	Adherence ≤77.5% vs. Control arm RR (95% CI)	Adherence >77.5% vs. Control arm RR (95% CI)	Adherence >77.5% vs. Adherence ≤77.5% RR (95% CI)
**Unadjusted model**	0.90 (0.56 to 1.42)	0.52 (0.29 to 0.95)	0.57 (0.32 to 0.99)
**Model adjusted for site characteristics**			
Number of hospital beds	0.91 (0.57 to 1.45)	0.48 (0.25 to 0.94)	0.61 (0.36 to 1.05)
Number of ICU beds	0.96 (0.6 to 1.54)	0.48 (0.25 to 0.94)	0.61 (0.35 to 1.09)
Number of ICU beds/hospital beds	0.88 (0.56 to 1.39)	0.50 (0.28 to 0.89)	0.56 (0.32 to 0.97)
Number of adult ICU beds	0.95 (0.6 to 1.52)	0.47 (0.25 to 0.89)	0.60 (0.34 to 1.06)
ICU type	– (–)	– (–)	– (–)
Hospital type	0.88 (0.55 to 1.42)	0.53 (0.29 to 0.97)	0.57 (0.31 to 1.06)
Teaching activity	0.84 (0.54 to 1.33)	0.52 (0.30 to 0.92)	"0.66 (0.38 to 1.16)
Transplant center	0.84 (0.52 to 1.37)	0.49 (0.28 to 0.87)	0.59 (0.33 to 1.04)
Number of annual brain death notifications [a]	0.89 (0.56 to 1.42)	0.55 (0.30 to 0.99)	0.54 (0.31 to 0.94)
**Model adjusted for potential donor characteristics**			
Age in years	0.88 (0.55 to 1.41)	0.52 (0.28 to 0.97)	0.55 (0.32 to 0.97)
Age >60 years	0.90 (0.56 to 1.44)	0.54 (0.29 to 0.99)	0.57 (0.32 to 1.00)
Female sex	0.90 (0.57 to 1.43)	0.54 (0.3 to 0.97)	0.57 (0.33 to 1.00)
SOFA score at enrollment	0.86 (0.55 to 1.35)	0.53 (0.3 to 0.95)	0.58 (0.34 to 1.01)
SAPS 3 score at ICU admission	0.88 (0.55 to 1.41)	0.52 (0.28 to 0.94)	0.56 (0.32 to 0.98)
Comorbidities			

© 2023 Westphal GA et al. *JAMA Network Open*

Diabetes mellitus	0.90 (0.57 to 1.43)	0.52 (0.29 to 0.95)	0.56 (0.32 to 0.98)
Hypertension	0.90 (0.57 to 1.43)	0.51 (0.28 to 0.94)	0.56 (0.32 to 0.98)
Renal failure requiring dialysis	0.89 (0.56 to 1.42)	0.52 (0.28 to 0.95)	0.57 (0.32 to 1.00)
Chronic respiratory disease	0.89 (0.56 to 1.41)	0.51 (0.28 to 0.94)	0.57 (0.32 to 0.99)
Heart failure	0.89 (0.56 to 1.42)	0.52 (0.29 to 0.95)	0.57 (0.32 to 1.00)
Chronic liver disease	– (–)	– (–)	– (–)
Cause of brain injury	0.90 (0.56 to 1.43)	0.54 (0.30 to 0.98)	"0.55 (0.31 to 0.97)
Use of antimicrobial medication [b]	0.92 (0.57 to 1.47)	0.52 (0.28 to 0.94)	0.56 (0.32 to 0.99)
Length of hospital stay in days before brain death diagnosis	0.88 (0.56 to 1.40)	0.52 (0.29 to 0.94)	0.57 (0.32 to 1.00)

Abbreviations: CI, confidence interval; HR, hazard ratio; ICU, intensive care unit; SAPS, Simplified Acute Physiology Score; SOFA, Sequential Organ Failure Assessment; Chronic respiratory disease is defined as restrictive, obstructive, or vascular disease severe enough to limit performance of the activities of daily living or chronic hypoxia, hypercapnia, polycythemia, pulmonary hypertension, or ventilator dependence; Chronic liver disease is defined as biopsy-proven cirrhosis or proven portal hypertension or previous history of hepatic insufficiency, encephalopathy, or coma.

[a] Number of annual brain death notifications considers the percentage of brain-dead potential organ donors clinically managed in the intensive care unit.

[b] Identified at the time of first clinical examination.

© 2023 Westphal GA et al. *JAMA Network Open*
