# Supplementary material for: Evidence-Based Checklist to Delay Cardiac Arrest in Brain-Dead Potential Organ Donors: The DONORS Cluster Randomized Clinical Trial
Source: JAMA Netw Open. 2023 Dec 14;6(12):e2346901. doi: 10.1001/jamanetworkopen.2023.46901 (PMC10722341; doi:10.1001/jamanetworkopen.2023.46901)
Supplement: Supplement 3. — Nonauthor Collaborators. DONORS Investigators and BRICNet [file jamanetwopen-e2346901-s003.pdf]

Supplemental Online Content: Nonauthor Collaborators
*First name, last name, and suffix (if applicable) are required and will appear in PubMed.

***Group Names: DONORS (Donation Network to Optimize Organ Recovery Study) Investigators and BRICNet (Brazilian Research in Intensive Care Network)**								
***First Name and Middle Initial(s)**	***Last Name**	***Suffix (eg, Jr, III)**	Academic Degrees	Institution	Location (city, state/province, country)	Role or Contribution, eg, chair, principal investigator	Group (if more than 1 Group listed in the byline) and/or Subgroup (eg, Steering Committee)	
Laercio M	de Stefano		MD	Hospital das Clínicas	Botucatu/ São Paulo/ Brazil	principal investigator		
Marina CA	Cleto		RN	Hospital das Clínicas	Botucatu/ São Paulo/ Brazil	subinvestigator		
Aline R	Zambrini		RN	Hospital das Clínicas	Botucatu/ São Paulo/ Brazil	subinvestigator		
Cintia	Banin		RN	Hospital das Clínicas	Botucatu/ São Paulo/ Brazil	subinvestigator		
Maria OG	Douglas		MD	Hospital Municipal Irmã Dulce	Praia Grande/ São Paulo/ Brazil	principal investigator		
Renato L	Borba		MD	Hospital Municipal Irmã Dulce	Praia Grande/ São Paulo/ Brazil	subinvestigator		
Daniela	Boni		MD	Hospital Municipal Irmã Dulce	Praia Grande/ São Paulo/ Brazil	subinvestigator		
Eliza MP	Monteiro		RN	Hospital Municipal Irmã Dulce	Praia Grande/ São Paulo/ Brazil	subinvestigator		
Airton LO	Manoel		MD	Hospital Paulistano	São Paulo/ São Paulo/ Brazil	principal investigator		
Ciro	Parioto Neto		MD	Hospital Paulistano	São Paulo/ São Paulo/ Brazil	subinvestigator		
Wilson J	Lovato		PhD	Hospital das Clínicas da Faculdade de	Ribeirão Preto/ São	principal investigator		
Rodrigo B	Cerantola		Psy	Hospital das Clínicas da Faculdade de	Ribeirão Preto/ São	subinvestigator		
Leonardo C	Palma		PhD	Hospital das Clínicas da Faculdade de	Ribeirão Preto/ São	subinvestigator		
Salomon O	Rojas		PhD	Hospital Beneficência Portuguesa	São Paulo/ São Paulo/ Brazil	principal investigator		
Viviane C	Veiga		PhD	Hospital Beneficência Portuguesa	São Paulo/ São Paulo/ Brazil	subinvestigator		
Luciana S	Freitas		RN	Hospital Beneficência Portuguesa	São Paulo/ São Paulo/ Brazil	subinvestigator		

Supplemental Online Content: Nonauthor Collaborators
*First name, last name, and suffix (if applicable) are required and will appear in PubMed.

*First Name and Middle Initial(s)	*Last Name	*Suffix (eg, Jr, III)	Academic Degrees	Institution	Location (city, state/province, country)	Role or Contribution, eg, chair, principal investigator	Group (if more than 1 Group listed in the byline) and/or Subgroup (eg, Steering Committee)	
Roberto	Marco		PhD	Irmandade da Santa Casa de Miseric	São Paulo/ São Paulo/ Brazil	principal investigator		
Fabiano	Hirata		MD	Irmandade da Santa Casa de Miseric	São Paulo/ São Paulo/ Brazil	subinvestigator		
Cinthia C	Vieira		MD	Irmandade da Santa Casa de Miseric	São Paulo/ São Paulo/ Brazil	subinvestigator		
Miriam	Jackiu		MD	Hospital São Paulo	São Paulo/ São Paulo/ Brazil	subinvestigator		
Alessandra D	Santiago		RN	Hospital São Paulo	São Paulo/ São Paulo/ Brazil	subinvestigator		
Márcia R	Bertin		MD	Santa Casa de Misericórdia	Sorocaba/ São Paulo/ Brazil	principal investigator		
Luiz	Otsubo		MD	Santa Casa de Misericórdia	Sorocaba/ São Paulo/ Brazil	subinvestigator		
Ana L P	Marques		RN	Santa Casa de Misericórdia	Sorocaba/ São Paulo/ Brazil	subinvestigator		
Josileide F	de Almeida		RN	Santa Casa de Misericórdia	Sorocaba/ São Paulo/ Brazil	subinvestigator		
Martha P	Torres		Psy	Hospital Geral	Sorocaba/ São Paulo/ Brazil	principal investigator		
Gileade G	dos Santos		RN	Hospital Geral	Taipas/ São Paulo/ Brazil	subinvestigator		
Márcia C	Gomes		SW	Hospital Regional do Vale do Paraíba	Taubaté/ São Paulo/ [illegible]	principal investigator	(in memorian)	
Caio L S	Nunes		MD	Hospital Regional do Vale do Paraíba	Taubaté/ São Paulo. Brazil	subinvestigator		
Felipe A	Moreira		RN	Hospital Regional do Vale do Paraíba	Taubaté/ São Paulo. Brazil	subinvestigator		
Daniele S M V	Simões		PhD	Coordenação Geral do Sistema Nacio	Brasília/ Distrito Federal/ Brazil	National coordination		
Leonardo d S	Reis		MBA	Coordenação Geral do Sistema Nacio	Brasília/ Distrito Federal/ Brazil	National coordination		
Mariane S L	de Souza		MsC	Coordenação Geral do Sistema Nacio	Brasília/ Distrito Federal/ Brazil	National coordination		
Rismaria M R	de Castro		MBA	Coordenação Geral do Sistema Nacio	Brasília/ Distrito Federal/ [illegible]	National coordination		

Supplemental Online Content: Nonauthor Collaborators
*First name, last name, and suffix (if applicable) are required and will appear in PubMed.

***First Name and Middle Initial(s)**	***Last Name**	***Suffix (eg, Jr, III)**	Academic Degrees	Institution	Location (city, state/province, country)	Role or Contribution, eg, chair, principal investigator	Group (if more than 1 Group listed in the byline) and/or Subgroup (eg, Steering Committee)	
Valeska M N	Gameiro		MBA	Coordenação Geral do Sistema Nacic	Brasília/ Distrito Federal/	National coordination		
Regiane C	Ferrari		Psy	Central Estadual de Transplantes do	Rio Branco/ Acre/ Brazil	Regional coordinator		
Daniela B	Ramos		MsC	Central Estadual de Transplantes de	Maceió/ Alagoas/ Brazil	Regional coordinator		
Leny N M	Passos		MD	Central Estadual de Transplantes do	Manaus/ Amazonas/ Brazil	Regional coordinator		
América C B M	Sodré		RN	Central Estadual de Transplantes da	Salvador/ Bahia/ Brazil	Regional coordinator		
Rita C M P	Pedrosa		MD	Central Estadual de Transplantes da	Salvador/ Bahia/ Brazil	Regional coordinator		
Eliana R B	de Almeida		MD	Central Estadual de Transplantes do	Fortaleza/ Ceará/ Brazil	Regional coordinator		
Camila	Hirata		RN	Central Estadual de Transplantes do	Brasília/ Distrito Federal/ Brazil	Regional coordinator		
Raquel DC	Matiello		RN	Central Estadual de Transplantes do	Vitória/ Espírito Santo/ Brazil	Regional coordinator		
Maria S	Machado		RN	Central Estadual de Transplantes do	Vitória/ Espírito Santo/ Brazil	Regional coordinator		
Fernando	Castro		MBA	Central Estadual de Transplantes de	Goiânia/ Goiás/ Brazil	Regional coordinator		
Gustavo P	Gonçalves		MD	Central Estadual de Transplantes de	Goiânia/ Goiás/ Brazil	Regional coordinator		
Maria I G	de Oliveira		MD	Central Estadual de Transplantes do	São Luis/ Maranhão/ Brazil	Regional coordinator		
Omar L	Cançado	Jr	MD	Central Estadual de Transplantes de	Belo Horizonte/ Minas	Regional coordinator		
Claire C	Miozzo		Pharm	Central Estadual de Transplantes do	Campo Grande/ Mato	Regional coordinator		
Gyanna L M M	Montenegro		MD	Central Estadual de Transplantes da	João Pessoa/ Paraíba/	Regional coordinator		
Noemy A C	Gomes		MD	Central Estadual de Transplantes da	Recife/ Pernambuco/Brazil	Regional coordinator		
Arlene T C G	Badoch		MD	Central Estadual de Transplantes do	Curitiba/ Paraná/ Brazil	Regional coordinator		
Rodrigo A	Sarlo		MD	Central Estadual de Transplantes do	Rio de Janeiro/ Rio de Janeiro/ Brazil	Regional coordinator		
Gabriel T M	Pereira		MD	Central Estadual de Transplantes do	Rio de Janeiro/ Rio de Janeiro/ Brazil	Regional coordinator		
Raissa M	Marques		MD	Central Estadual de Transplantes do	Natal/ Rio Grande do Norte/ Brazil	Regional coordinator		
Suely L A	Toledo		RN	Central Estadual de Transplantes de	Porto Velho/ Rondônia/ Brazil	Regional coordinator		
Ricardo K	Ruhling		MD	Central Estadual de Transplantes do	Porto Alegre/ Rio Grande	Regional coordinator		

***First Name and Middle Initial(s)**	***Last Name**	***Suffix (eg, Jr, III)**	Academic Degrees	Institution	Location (city, state/province, country)	Role or Contribution, eg, chair, principal investigator	Group (if more than 1 Group listed in the byline) and/or Subgroup (eg, Steering Committee)	
Benito O	Fernandez		RN	Central Estadual de Transplantes de	Aracajú/ Sergipe/ Brazil	Regional coordinator		
Agenor	Spalini		MD	Central Estadual de Transplantes de São Paulo	São Paulo/ São Paulo/ Brazil	Regional coordinator		
Francisco A S	Monteiro		MD	Central Estadual de Transplantes de São Paulo	São Paulo/ São Paulo/ Brazil	Regional coordinator		
Marizete P	Medeiros		MD	Central Estadual de Transplantes de São Paulo	São Paulo/ São Paulo/ Brazil	Regional coordinator		
Caroline L	de Oliveira		Medical Scientist	Moinhos de Vento Hospital	Porto Alegre/Rio Grande do Sul/ Brazil	Study coordinator team		
Gabriela S	Rech		Statisticia n	Moinhos de Vento Hospital	Porto Alegre/Rio Grande do Sul/ Brazil	Study coordinator team		
Patrícia S	Benck		Psy	Moinhos de Vento Hospital	Porto Alegre/Rio Grande do Sul/ Brazil	Study coordinator team		
Silvana R	Tomé		SW	Moinhos de Vento Hospital	Porto Alegre/Rio Grande do Sul/ Brazil	Study coordinator team		
Silvia EM	de Mendonça		Attorney	Moinhos de Vento Hospital	Porto Alegre/Rio Grande do Sul/ Brazil	Study coordinator team		
Vitor A	Wecki		Statisticia n	Moinhos de Vento Hospital	Porto Alegre/Rio Grande do Sul/ Brazil	Study coordinator team		
Miriam MV	Machado		PhD	Centro Hospitalar Unimed	Joinville/Santa Catarina/ Brazil	Donor managment instructor		
Carmen	Segovia		RN	Organización Nacional de Trasplantes	Madrid/ Spain	Family interview instructor		
Aline	Ghellere		RN	Central Estadual de Transplantes de Santa Catarina	Florianópolis/ Santa Catarina/ Brazil	Family interview instructor		
Charlene V	da Silva		RN	Central Estadual de Transplantes de Santa Catarina	Florianópolis/ Santa Catarina/ Brazil	Family interview instructor		
Dagoberto F	da Rocha		RN	Central Estadual de Transplantes do Rio Grande do Sul	Porto Alegre/Rio Grande do Sul/ Brazil	Family interview instructor		
Eduardo	Berbigier		RN	Central Estadual de Transplantes de Santa Catarina	Florianópolis/ Santa Catarina/ Brazil	Family interview instructor		
Edvaldo	Leal		MD	Central Estadual de Transplantes de São Paulo	São Paulo/ São Paulo/ Brazil	Family interview instructor		

Supplemental Online Content: Nonauthor Collaborators
*First name, last name, and suffix (if applicable) are required and will appear in PubMed.

*First Name and Middle Initial(s)	*Last Name	*Suffix (eg, Jr, III)	Academic Degrees	Institution	Location (city, state/province, country)	Role or Contribution, eg, chair, principal investigator	Group (if more than 1 Group listed in the byline) and/or Subgroup (eg, Steering Committee)	
Felipe	Pfuetzenreifer		MD	Central Estadual de Transplantes de Santa Catarina	Florianópolis/ Santa Catarina/ Brazil	Family interview instructor		
Fernando	Bourscheit		MD	Central Estadual de Transplantes do Rio Grande do Sul	Porto Alegre/Rio Grande do Sul/ Brazil	Family interview instructor		
José L	Toribio		MD	Central Estadual de Transplantes do Rio Grande do Sul	Porto Alegre/Rio Grande do Sul/ Brazil	Family interview instructor		
Luana	Tannous		MD	Central Estadual de Transplantes do Paraná	Curitiba/ Paraná/ Brazil	Family interview instructor		
Luana	Heberle		RN	Central Estadual de Transplantes do Paraná	Curitiba/ Paraná/ Brazil	Family interview instructor		
Neide	Knihs		RN	Central Estadual de Transplantes de Santa Catarina	Florianópolis/ Santa Catarina/ Brazil	Family interview instructor		
Paulo RC	Cardoso		MD	Hospital de Clínicas de Porto Alegre	Porto Alegre/Rio Grande do Sul/ Brazil	Family interview instructor		
Marcelo	Grando		MD	Hospital de Urgência e Emergência de Rio Branco	Rio Branco/Acre/ Brazil	principal investigator		
Ludmylla BV	Veras		MD	Hospital de Urgência e Emergência de Rio Branco	Rio Branco/Acre/ Brazil	subinvestigator		
Gigliane MA	Albuquerque		RN	Hospital de Urgência e Emergência de Rio Branco	Rio Branco/Acre/ Brazil	subinvestigator		
Régis A	Hashimoto		MD	Hospital de Urgência e Emergência de Rio Branco	Rio Branco/Acre/ Brazil	subinvestigator		
Mateus RT	Teles		MD	Hospital das Clínicas de Rio Branco	Rio Branco/Acre/ Brazil	principal investigator		
Rosely B	Cruz		MD	Hospital das Clínicas de Rio Branco	Rio Branco/Acre/ Brazil	subinvestigator		
Nelson AC	Pinheiro	Jr	RN	Hospital das Clínicas de Rio Branco	Rio Branco/Acre/ Brazil	subinvestigator		
Janapina MMF	de Moraes		MD	Hospital Geral Professor Osvaldo Brandão Vilela	Maceió/Alagoas/ Brazil	principal investigator		
Claudete M	Balzan		RN	Hospital Geral Professor Osvaldo Brandão Vilela	Maceió/Alagoas/ Brazil	subinvestigator		

Supplemental Online Content: Nonauthor Collaborators
*First name, last name, and suffix (if applicable) are required and will appear in PubMed.

***First Name and Middle Initial(s)**	***Last Name**	***Suffix (eg, Jr, III)**	Academic Degrees	Institution	Location (city, state/province, country)	Role or Contribution, eg, chair, principal investigator	Group (if more than 1 Group listed in the byline) and/or Subgroup (eg, Steering Committee)	
Lúcia RA	Leite		RN	Hospital Geral Professor Osvaldo Brandão Vilela	Maceió/Alagoas/ Brazil	subinvestigator		
Lis DP	Oliveira		MD	Hospital Geral Professor Osvaldo Brandão Vilela	Maceió/Alagoas/ Brazil	subinvestigator		
Thattyane B	Pereira		MD	Hospital de Pronto Socorro João Paulo II	Porto Velho/ Rondônia/ Brazil	principal investigator		
Edwin F	Novillo		MD	Hospital de Pronto Socorro João Paulo II	Porto Velho/ Rondônia/ Brazil	subinvestigator		
Maxwendell G	Batista		MD	Hospital de Pronto Socorro João Paulo II	Porto Velho/ Rondônia/ Brazil	subinvestigator		
Silvecler C	de Campos		MD	Hospital de Pronto Socorro João Paulo II	Porto Velho/ Rondônia/ Brazil	subinvestigator		
Marcelo S	Ferreira		MD	Hospital de Pronto Socorro Dr. João Lúcio Pereira Machado	Manaus/Amazonas/ Brazil	principal investigator		
Helen CA	Bezerra		RN	Hospital de Pronto Socorro Dr. João Lúcio Pereira Machado	Manaus/Amazonas/ Brazil	subinvestigator		
Paulo HL	Matos		RN	Hospital de Pronto Socorro Dr. João Lúcio Pereira Machado	Manaus/Amazonas/ Brazil	subinvestigator		
Janaína	Feijó		MD	Hospital de Urgência de Sergipe	Aracaju/Sergipe / Brazil	principal investigator		
Dernivania A	Ferreira		RN	Hospital de Urgência de Sergipe	Aracaju/Sergipe / Brazil	subinvestigator		
Ana PRB	Machado		RN	Hospital de Urgência de Sergipe	Aracaju/Sergipe / Brazil	subinvestigator		
Poliana N	Santos		RN	Hospital de Urgência de Sergipe	Aracaju/Sergipe / Brazil	subinvestigator		
Felipe FR	de Souza		MD	Hospital Geral Cleriston Andrade	Feira de Santana/Bahia/ Brazil	subinvestigator		
Daniela C	de Oliveira		RN	Hospital Geral Cleriston Andrade	Feira de Santana/Bahia/ Brazil	subinvestigator		
Graças M	Dias Reis		MD	Hospital Geral Cleriston Andrade	Feira de Santana/Bahia/ Brazil	subinvestigator		
Ana V	Rolim		MD	Hospital Instituto Dr. José Frota	Fortaleza/Ceará/ Brazil	principal investigator		
Samira R	Magalhães		RN	Hospital Instituto Dr. José Frota	Fortaleza/Ceará/ Brazil	subinvestigator		
Cleiriane	Reis			Hospital Instituto Dr. José Frota	Fortaleza/Ceará/ Brazil	subinvestigator		
Joel I	Costa		MD	Hospital Geral de Fortaleza	Fortaleza/Ceará/ Brazil	principal investigator		
Larissa SP	Carneiro		MD	Hospital Geral de Fortaleza	Fortaleza/Ceará/ Brazil	subinvestigator		

Supplemental Online Content: Nonauthor Collaborators
*First name, last name, and suffix (if applicable) are required and will appear in PubMed.

*First Name and Middle Initial(s)	*Last Name	*Suffix (eg, Jr, III)	Academic Degrees	Institution	Location (city, state/province, country)	Role or Contribution, eg, chair, principal investigator	Group (if more than 1 Group listed in the byline) and/or Subgroup (eg, Steering Committee)	
Márcia MVS	Passos		RN	Hospital Geral de Fortaleza	Fortaleza/Ceará/ Brazil	subinvestigator		
Gustavo M	dos Santos		MD	Hospital Regional do Cariri	Juazeiro do Norte/Ceará/ Brazil	principal investigator		
José WB	de Souza		RN	Hospital Regional do Cariri	Juazeiro do Norte/Ceará/ Brazil	subinvestigator		
Bruna BO	Marinho		RN	Hospital Regional do Cariri	Juazeiro do Norte/Ceará/ Brazil	subinvestigator		
Luiz DP	Salles	Jr	MD	Irmandade Santa Casa de Misericórdia de Sobral	Sobral/Ceará/ Brazil	principal investigator		
José H	Gurgel		MD	Irmandade Santa Casa de Misericórdia de Sobral	Sobral/Ceará/ Brazil	subinvestigator		
Iranildo P	Fontenele		RN	Irmandade Santa Casa de Misericórdia de Sobral	Sobral/Ceará/ Brazil	subinvestigator		
Layanny TL	Bezerra		RN	Irmandade Santa Casa de Misericórdia de Sobral	Sobral/Ceará/ Brazil	subinvestigator		
Cristiano A	Costa		MD	Irmandade Santa Casa de Misericórdia de Sobral	Sobral/Ceará/ Brazil	subinvestigator		
Israel F	da Costa		RN	Hospital Regional Norte	Sobral/Ceará/ Brazil	subinvestigator		
Diego BS	Pinheiro		RN	Hospital Regional Norte	Sobral/Ceará/ Brazil	subinvestigator		
Denise M	de Moura		RN	Hospital Regional Norte	Sobral/Ceará/ Brazil	subinvestigator		
Suzana	Cantidio		RN	Hospital Regional Tarcísio de Vasconcelos Maia	Mossoró/Rio Grande do Norte/ Brazil	subinvestigator		
Jéssica PSL	Moreira		RN	Hospital Regional Tarcísio de Vasconcelos Maia	Mossoró/Rio Grande do Norte/ Brazil	subinvestigator		
Telma A	Belém		SW	Hospital Regional Tarcísio de Vasconcelos Maia	Mossoró/Rio Grande do Norte/ Brazil	subinvestigator		
Symoni BFQ	Florentino		Psy	Hospital Regional Tarcísio de Vasconcelos Maia	Mossoró/Rio Grande do Norte/ Brazil	subinvestigator		
Bruna S	Carvalho		RN	Hospital Regional Tarcísio de Vasconcelos Maia	Mossoró/Rio Grande do Norte/ Brazil	subinvestigator		
Ciro L	Mendes		MD	Hospital Alberto Urquiza Wanderley	João Pessoa/Paraíba/ Brazil	principal investigator		

Supplemental Online Content: Nonauthor Collaborators
*First name, last name, and suffix (if applicable) are required and will appear in PubMed.

*First Name and Middle Initial(s)	*Last Name	*Suffix (eg, Jr, III)	Academic Degrees	Institution	Location (city, state/province, country)	Role or Contribution, eg, chair, principal investigator	Group (if more than 1 Group listed in the byline) and/or Subgroup (eg, Steering Committee)	
Igor M	do Nascimento		MD	Hospital Alberto Urquiza Wanderley	João Pessoa/Paraíba/ Brazil	subinvestigator		
Paloma E	Araújo		RN	Hospital Alberto Urquiza Wanderley	João Pessoa/Paraíba/ Brazil	subinvestigator		
Maryjane AO	Araújo		RN	Hospital Alberto Urquiza Wanderley	João Pessoa/Paraíba/ Brazil	subinvestigator		
Alexandre AG	Alves		PhD	Hospital Municipal Djalma Marques	São Luis/Maranhão/ Brazil	principal investigator		
Heloisa RFO	Lima		RN	Hospital Municipal Djalma Marques	São Luis/Maranhão/ Brazil	subinvestigator		
Silvia HCA	Carvalho		RN	Hospital Municipal Djalma Marques	São Luis/Maranhão/ Brazil	subinvestigator		
Clayton	Aragão		MD	Hospital Municipal Djalma Marques	São Luis/Maranhão/ Brazil	subinvestigator		
Marko AF	Santos		MD	Hospital Dr. Carlos Macieira	São Luis/Maranhão/ Brazil	principal investigator		
Luiza MN	Moraes		RN	Hospital Dr. Carlos Macieira	São Luis/Maranhão/ Brazil	subinvestigator		
Henrique LCN	Sobrinho		Phy	Hospital Dr. Carlos Macieira	São Luis/Maranhão/ Brazil	subinvestigator		
Sylvia HAL	Siqueira		MD	Hospital da Restauração	Recife/Pernambuco/ Brazi	subinvestigator		
Janaína R	Silva		RN	Hospital da Restauração	Recife/Pernambuco/ Brazi	subinvestigator		
Samyra P	Moraes		RN	Hospital de Ensino Doutor Washingt	Petrolina/Pernambuco/ Br	subinvestigator		
Janaína CW	Carvalho		RN	Hospital de Ensino Doutor Washingt	Petrolina/Pernambuco/ Br	subinvestigator		
Luiz H	Silva		MD	Hospital de Base do Distrito Federal	Brasília/Distrito Federal/ B	principal investigator		
Viviane M	Silva		RN	Hospital de Base do Distrito Federal	Brasília/Distrito Federal/ B	subinvestigator		
Jander P	Fornaciari		MD	Hospital Estadual de Urgência e Eme	Vitória/Espírito Santo/ Bra	principal investigator		
Ana PN	Curty		RN	Hospital Estadual de Urgência e Eme	Vitória/Espírito Santo/ Bra	subinvestigator		
Ivens G	Soares		RN	Hospital Estadual de Urgência e Eme	Vitória/Espírito Santo/ Bra	subinvestigator		
Caroline S	Pimentel		Psy	Hospital Estadual de Urgência e Eme	Vitória/Espírito Santo/ Bra	subinvestigator		
Marco AM	Castilho	Jr	MD	Hospital de Urgência de Goiânia	Goiânia/ Goiás/ Brazil	principal investigator		
Tânia LAA	Veiga		RN	Hospital de Urgência de Goiânia	Goiânia/ Goiás/ Brazil	subinvestigator		
Frederico B	Carvalho		MD	Hospital João XXIII	Belo Horizonte/Minas Gera	principal investigator		
Natasha P	Ferreira		RN	Hospital João XXIII	Belo Horizonte/Minas Gera	subinvestigator		
Sylmara JZ	Freitas		MD	Hospital João XXIII	Belo Horizonte/Minas Gera	subinvestigator		
Fernanda CP	Sousa		RN	Hospital João XXIII	Belo Horizonte/Minas Gera	subinvestigator		
Chen	Laura		RN	Hospital João XXIII	Belo Horizonte/Minas Gera	subinvestigator		

Supplemental Online Content: Nonauthor Collaborators
*First name, last name, and suffix (if applicable) are required and will appear in PubMed.

***First Name and Middle Initial(s)**	***Last Name**	***Suffix (eg, Jr, III)**	Academic Degrees	Institution	Location (city, state/province, country)	Role or Contribution, eg, chair, principal investigator	Group (if more than 1 Group listed in the byline) and/or Subgroup (eg, Steering Committee)	
Vandack A	Nobre	Jr	PhD	Hospital das Clínicas da Universidade Federal de Minas Gerais	Belo Horizonte/Minas Gera	principal investigator		
Christiane FMHM	Pereira		RN	Hospital das Clínicas da Universidade Federal de Minas Gerais	Belo Horizonte/Minas Gera	subinvestigator		
Amélia C	Gomes		Psy	Hospital das Clínicas da Universidade Federal de Minas Gerais	Belo Horizonte/Minas Gera	subinvestigator		
Jeová F	Oliveira		MD	Hospital Universitário Ciências M	Belo Horizonte/Minas Gera	principal investigator		
Paula G	Nasser		RN	Hospital Universitário Ciências M	Belo Horizonte/Minas Gera	subinvestigator		
Alexandre S	Ferreira		Phy	Hospital Universitário Ciências M	Belo Horizonte/Minas Gera	subinvestigator		
Mara R	Moura		RN	Irmandade Santa Casa de Misericórdia de Belo Horizonte	Belo Horizonte/Minas Gera	principal investigator		
Claudio D	Oliveira		MD	Irmandade Santa Casa de Misericórdia de Belo Horizonte	Belo Horizonte/Minas Gera	subinvestigator		
Andressa S	Moreira		RN	Irmandade Santa Casa de Misericórdia de Belo Horizonte	Belo Horizonte/Minas Gera	subinvestigator		
Raquel B	Santiago		RN	Irmandade Santa Casa de Misericórdia de Belo Horizonte	Belo Horizonte/Minas Gera	subinvestigator		
Marina F	Oliveira		RN	Irmandade Santa Casa de Misericórdia de Belo Horizonte	Belo Horizonte/Minas Gera	subinvestigator		
Ana OS	Neves		RN	minist	Campo Grande/Mato Grosso do Sul/ Brazil	subinvestigator		
Rodrigo CG	Silva		RN	Santa Casa de Campo Grande	Campo Grande/Mato Grosso do Sul/ Brazil	subinvestigator		
Roberto	Almeida		MD	Hospital Municipal Padre Germano Lauck	Foz do Iguaçu/Paraná/ Brazil	principal investigator		
Karin A	Zili		RN	Hospital Municipal Padre Germano Lauck	Foz do Iguaçu/Paraná/ Brazil	subinvestigator		
Alexsandro JS	Fernandes		RN	Hospital Municipal Padre Germano Lauck	Foz do Iguaçu/Paraná/ Brazil	subinvestigator		
Ana L	Mezzaroba		MD	Hospital Evangélico de Londrina	Londrina/Paraná/ Brazil	subinvestigator		

Supplemental Online Content: Nonauthor Collaborators
*First name, last name, and suffix (if applicable) are required and will appear in PubMed.

*First Name and Middle Initial(s)	*Last Name	*Suffix (eg, Jr, III)	Academic Degrees	Institution	Location (city, state/province, country)	Role or Contribution, eg, chair, principal investigator	Group (if more than 1 Group listed in the byline) and/or Subgroup (eg, Steering Committee)	
Josiane	Festti		MD	Hospital Evangélico de Londrina	Londrina/Paraná/ Brazil	subinvestigator		
Alexandre S	Larangeira		MD	Hospital Evangélico de Londrina	Londrina/Paraná/ Brazil	subinvestigator		
Thomas M	Dhaese		MD	Hospital Universitário Regional dos Campos Gerais	Ponta Grossa/Paraná/ Brazil	principal investigator		
Simone Macedo	Hanke		RN	Hospital Universitário Regional dos Campos Gerais	Ponta Grossa/Paraná/ Brazil	subinvestigator		
Guilherme	Arcaro		RN	Hospital Universitário Regional dos Campos Gerais	Ponta Grossa/Paraná/ Brazil	subinvestigator		
Pericles AD	Duarte		MD	Hospital Universitário de Cascavel do Oeste do Paraná	Cascavel/Paraná/ Brazil	principal investigator		
Elaine F	Padilha		RN	Hospital Universitário de Cascavel do Oeste do Paraná	Cascavel/Paraná/ Brazil	subinvestigator		
Cleber	Tchaicka		MD	Hospital Universitário de Cascavel do Oeste do Paraná	Cascavel/Paraná/ Brazil	subinvestigator		
Lizandra O	Ayres		RN	Hospital Universitário de Cascavel do Oeste do Paraná	Cascavel/Paraná/ Brazil	subinvestigator		
Marcos T	Tanita		MD	Hospital Universitário Regional do Norte do Paraná	Londrina/Paraná/ Brazil	subinvestigator		
Carlos A	Oliveira		RN	Hospital Universitário Regional do Norte do Paraná	Londrina/Paraná/ Brazil	subinvestigator		
Almir	Germano		MD	Hospital Universitário de Maringá	Maringá/Paraná/ Brazil	principal investigator		
Catia M	Dell´Agnolo		RN	Hospital Universitário de Maringá	Maringá/Paraná/ Brazil	subinvestigator		
Rosane A	Freitas		RN	Hospital Universitário de Maringá	Maringá/Paraná/ Brazil	subinvestigator		
Ellen S	Barbosa		MD	Irmandade Santa Casa de Misericórdia de Maringá	Maringá/Paraná/ Brazil	principal investigator		
Vanessa M	Mezzavila		RN	Irmandade Santa Casa de Misericórdia de Maringá	Maringá/Paraná/ Brazil	subinvestigator		
Renata	Santos			Irmandade Santa Casa de Misericórdia de Maringá	Maringá/Paraná/ Brazil	subinvestigator		
Pedro	Rigon	Jr		Irmandade Santa Casa de Misericórdia de Maringá	Maringá/Paraná/ Brazil	subinvestigator		
Itamar	Weiwanko		RN	Hospital Bom Jesus de Toledo	Toledo/Paraná/ Brazil	principal investigator		
Cristiano	Mroginski		MD	Hospital Bom Jesus de Toledo	Toledo/Paraná/ Brazil	subinvestigator		

Supplemental Online Content: Nonauthor Collaborators
*First name, last name, and suffix (if applicable) are required and will appear in PubMed.

***First Name and Middle Initial(s)**	***Last Name**	***Suffix (eg, Jr, III)**	Academic Degrees	Institution	Location (city, state/province, country)	Role or Contribution, eg, chair, principal investigator	Group (if more than 1 Group listed in the byline) and/or Subgroup (eg, Steering Committee)	
Waldir A	Pasa	Jr	MD	Hospital Bom Jesus de Toledo	Toledo/Paraná/ Brazil	subinvestigator		
Maikel	Ramthun		MD	Hospital Bom Jesus de Ponta Gro	Ponta Grossa/Paraná/ Brazil	principal investigator		
Jullye C	Pereira		RN	Hospital Bom Jesus de Ponta Gro	Ponta Grossa/Paraná/ Brazil	subinvestigator		
César AB	Flores		RN	Hospital Bom Jesus de Ponta Gro	Ponta Grossa/Paraná/ Brazil	subinvestigator		
Patrícia B	Cwiertnia		Psy	Hospital Bom Jesus de Ponta Gro	Ponta Grossa/Paraná/ Brazil	subinvestigator		
Ricardo GZ	Risson		MD	Hospital Nossa Senhora do Rocio de Campo Largo	Campo Largo/Paraná/ Braz	principal investigator		
Joseane A	Taborda		RN	Hospital Nossa Senhora do Rocio de Campo Largo	Campo Largo/Paraná/ Braz	subinvestigator		
Mayara F	Vieira		RN	Hospital Nossa Senhora do Rocio de Campo Largo	Campo Largo/Paraná/ Braz	subinvestigator		
Tatiana E	Cassolli		MD	Hospital e Maternidade Angelina Caron	Campina Grande do Sul/Paraná/ Brazil	principal investigator		
Mariana P	Singer		MD	Hospital e Maternidade Angelina	Campina Grande do Sul/Paraná/ Brazil	subinvestigator		
Rosiane O	Pereira		RN	Hospital e Maternidade Angelina	Campina Grande do Sul/Paraná/ Brazil	subinvestigator		
Jaciara R	Oliveira		RN	Hospital e Maternidade Angelina	Campina Grande do Sul/Paraná/ Brazil	subinvestigator		
Melina AS	Lemos		MD	Hospital Santa Rita de Maringá	Maringá/Paraná/ Brazil	principal investigator		
Vivianne CB	Toniol		RN	Hospital Santa Rita de Maringá	Maringá/Paraná/ Brazil	subinvestigator		
Mariza A	Souza		RN	Hospital Santa Rita de Maringá	Maringá/Paraná/ Brazil	subinvestigator		
Ângelo Y	Hayashi		MD	Hospital Norte Paranaense	Arapongas/Paraná/ Brazil	principal investigator		
Priscila LL	Paula		RN	Hospital Norte Paranaense	Arapongas/Paraná/ Brazil	subinvestigator		
Elza de Lara	Bezerra		RN	Hospital Norte Paranaense	Arapongas/Paraná/ Brazil	subinvestigator		
Fernanda GZ	Borges		MD	Hospital São Vicente de Paulo Gu	Guarapuava/Paraná/ Brazil	principal investigator		

Supplemental Online Content: Nonauthor Collaborators
*First name, last name, and suffix (if applicable) are required and will appear in PubMed.

*First Name and Middle Initial(s)	*Last Name	*Suffix (eg, Jr, III)	Academic Degrees	Institution	Location (city, state/province, country)	Role or Contribution, eg, chair, principal investigator	Group (if more than 1 Group listed in the byline) and/or Subgroup (eg, Steering Committee)	
Elaine S	Ramos		RN	Hospital São Vicente de Paulo Gu	Guarapuava/Paraná/ Brazil	subinvestigator		
Cibele A	Marochi		RN	Hospital São Vicente de Paulo Gu	Guarapuava/Paraná/ Brazil	subinvestigator		
Jessyca	Braga		RN	Hospital São Vicente de Paulo Gu	Guarapuava/Paraná/ Brazil	subinvestigator		
Alexander O	Sodré		MD	Hospital Geral de Nova Iguaçu	Nova Iguaçu/ Rio de Janeir	principal investigator		
Letícia AP	Entrago		MD	Hospital Geral de Nova Iguaçu	Nova Iguaçu/ Rio de Janeir	subinvestigator		
Thiago M	Barcelos		RN	Hospital Geral de Nova Iguaçu	Nova Iguaçu/ Rio de Janeir	subinvestigator		
Roberta C	de Jesus		RN	Hospital Geral de Nova Iguaçu	Nova Iguaçu/ Rio de Janeir	subinvestigator		
Vitor M	Vianna		MD	Hospital Estadual Getúlio Vargas	Rio de Janeiro/ Rio de Jane	principal investigator		
Mônica SFS	de Melo		MD	Hospital Estadual Getúlio Vargas	Rio de Janeiro/ Rio de Jane	subinvestigator		
Tais CB	Vaz		RN	Hospital Estadual Getúlio Vargas	Rio de Janeiro/ Rio de Jane	subinvestigator		
Flávio E	Santos		RN	Hospital Estadual Getúlio Vargas	Rio de Janeiro/ Rio de Jane	subinvestigator		
Vladimir S	Begni		RN	Hospital Estadual Getúlio Vargas	Rio de Janeiro/ Rio de Jane	subinvestigator		
Fábio F	Cardoso		MD	Hospital Bruno Born	Lajeado/ Rio Grande do Su	principal investigator		
Lucas	Mallmann		MD	Hospital Bruno Born	Lajeado/ Rio Grande do Su	subinvestigator		
Adriana	Calvi		RN	Hospital Bruno Born	Lajeado/ Rio Grande do Su	subinvestigator		
Nelson B	Franco	Neto	RN	Hospital Bruno Born	Lajeado/ Rio Grande do Su	subinvestigator		
Angélica	Oliveira		RN	Hospital Bruno Born	Lajeado/ Rio Grande do Su	subinvestigator		
Flávio AC	Alves		MD	Hospital Cristo Redentor	Porto Alegre/ Rio Grande d	principal investigator		
Manoel NO	Silveira		RN	Hospital Cristo Redentor	Porto Alegre/ Rio Grande d	subinvestigator		
Deisi LO	da Fonseca		MD	Hospital Cristo Redentor	Porto Alegre/ Rio Grande d	subinvestigator		
Susana	Santini		RN	Hospital Cristo Redentor	Porto Alegre/ Rio Grande d	subinvestigator		
Edison M	Rodrigues Filho		MD	Irmandade da Santa Casa de Mise	Porto Alegre/ Rio Grande d	principal investigator		
Fernanda P	Bonow		MD	Irmandade da Santa Casa de Mise	Porto Alegre/ Rio Grande d	subinvestigator		
Ruth	Susin		MD	Irmandade da Santa Casa de Mise	Porto Alegre/ Rio Grande d	subinvestigator		
Kellen PM	Machado		RN	Irmandade da Santa Casa de Mise	Porto Alegre/ Rio Grande d	subinvestigator		
Danielle M	de Aguiar		MD	Hospital de Pronto Socorro Nelso	Porto Alegre/ Rio Grande d	principal investigator		
Caroline S	Scheneider		MD	Hospital de Pronto Socorro Nelso	Porto Alegre/ Rio Grande d	subinvestigator		
Lidiane C	Braz		MD	Hospital de Pronto Socorro Nelso	Porto Alegre/ Rio Grande d	subinvestigator		
Carlos FP	do Bem		MD	Hospital de Pronto Socorro Nelso	Porto Alegre/ Rio Grande d	subinvestigator		

Supplemental Online Content: Nonauthor Collaborators
*First name, last name, and suffix (if applicable) are required and will appear in PubMed.

*First Name and Middle Initial(s)	*Last Name	*Suffix (eg, Jr, III)	Academic Degrees	Institution	Location (city, state/province, country)	Role or Contribution, eg, chair, principal investigator	Group (if more than 1 Group listed in the byline) and/or Subgroup (eg, Steering Committee)	
Tatiana H	Rech		MD	Hospital de Clínicas de Porto Ale	Porto Alegre/ Rio Grande d	subinvestigator		
Vivian W	de Aquino		MD	Hospital de Pronto Socorro de Po	Porto Alegre/ Rio Grande d	subinvestigator		
Luciano O	Teixeira		MD	Universitário São Francisco de Pa	Pelotas/ Rio Grande do Sul	subinvestigator		
Viviane A	Mendonça		RN	Universitário São Francisco de Pa	Pelotas/ Rio Grande do Sul	subinvestigator		
Silvia Z	Bizarro		RN	Universitário São Francisco de Pa	Pelotas/ Rio Grande do Sul	subinvestigator		
José O	Calvete		MD	Hospital São Vicente de Paulo	Passo Fundo/ Rio Grande d	principal investigator		
Lina	Maito		MD	Hospital São Vicente de Paulo	Passo Fundo/ Rio Grande d	subinvestigator		
Sabrina F	Henrich		MD	Hospital São Vicente de Paulo	Passo Fundo/ Rio Grande d	subinvestigator		
Larissa A	PeLlizzaro		MD	Hospital São Vicente de Paulo	Passo Fundo/ Rio Grande d	subinvestigator		
Giovana C	Mecatti		MD	Hospital Universitário São Franci	Bragança Paulista/São	principal investigator		
Thiago C	Filiponi		MD	Hospital Universitário São Franci	Bragança Paulista/São	subinvestigator		
Felipe FP	Barbosa		MD	Hospital Universitário São Franci	Bragança Paulista/São	subinvestigator		
Flávia	Gozzoli		Psy	Hospital Universitário São Franci	Bragança Paulista/São	subinvestigator		
André S	Ribeiro		MD	Casa de Saúde de Santos	Santos/São Paulo/Brazil	principal investigator		
Paulo HP	Rosateli		MD	Casa de Saúde de Santos	Santos/São Paulo/Brazil	subinvestigator		
Zeher M	Waked		MD	Casa de Saúde de Santos	Santos/São Paulo/Brazil	subinvestigator		
Ana P	Quintal		RN	Casa de Saúde de Santos	Santos/São Paulo/Brazil	subinvestigator		
Suzana MA	Lobo		MD	Hospital de Base de São José do I	São José do Rio Preto/Sã	subinvestigator		
Regiane	Sampaio		RN	Hospital de Base de São José do I	São José do Rio Preto/Sã	subinvestigator		
Marcos	Morais		RN	Hospital de Base de São José do I	São José do Rio Preto/Sã	subinvestigator		
James L	Rol		RN	Hospital de Base de São José do I	São José do Rio Preto/Sã	subinvestigator		
**AUTHORS**								
Bianca R	Orlando		MD	Universitário São Francisco de Pa	Pelotas/ Rio Grande do Sul	Center Principal investigator (top recruiter)		
Cintia MC	Grion		PhD	Hospital Universitário Regional do N	Londrina, Paraná, Brazil	Center Principal investigator (top recruiter)		
Fernando A	Bezerra		MD	Hospital Regional Tarcísio de Vascon	Mossoró, Rio Grande do N	Center Principal investigator (top recruiter)		
Fernando R	Roman		MD	Hospital Bom Jesus	Toledo, Paraná, Brazil	Center Principal investigator (top recruiter)		
Francisco O	Leite	Jr	MD	Irmandade Santa Casa de Misericórdia de Sobral	Sobral/Ceará/ Brazil	Center Principal investigator (top recruiter)		
Íris LF	Siqueira		MD	Hospital de Urgência e Emergência de Rio Branco	Rio Branco/Acre/ Brazil	Center Principal investigator (top recruiter)		

Supplemental Online Content: Nonauthor Collaborators
*First name, last name, and suffix (if applicable) are required and will appear in PubMed.

*First Name and Middle Initial(s)	*Last Name	*Suffix (eg, Jr, III)	Academic Degrees	Institution	Location (city, state/province, country)	Role or Contribution, eg, chair, principal investigator	Group (if more than 1 Group listed in the byline) and/or Subgroup (eg, Steering Committee)	
João FP	Oliveira		MD	Hospital de Base de São José do F	São José do Rio Preto/Sã	Center Principal investigator (top recruiter)		
LucioC	de Oliveira	Jr	MD	Hospital Geral Cleriston Andrade	Feira de Santana, Bahia, Br	Center Principal investigator (top recruiter)		
Maria FRB	de Melo		MD	Hospital da Restauração	Recife, Pernambuco, Brazil	Center Principal investigator (top recruiter)		
Patrícia BGP	Leal		MD	Santa Casa de Campo Grande	Campo Grande/Mato Grosso do Sul/ Brazil	Center Principal investigator (top recruiter)		
Pedro C	Diniz			3Hospital Universitário, Universidade	Petrolina, Pernambuco, Br	Center Principal investigator (top recruiter)		
Rafael B	Moraes		PhD	Hospital de Clínicas de Porto Alegre	Porto Alegre, Rio Grande d	Center Principal investigator (top recruiter)		
Daniela FS	Pontes			General Coordination Office, Nationa	Brasília, Distrito Federal, B	National coordination		
Joselio EA	Queiroz		MSc	General Coordination Office, Nationa	Brasília, Distrito Federal, B	National coordination		
**STEERING COMMITTEE**								
Glauco A	Westphal		PhD	Hospital Moinhos de Vento	Porto Alegre, Rio Grande do Sul, Brazil		Steering Committee	
Caroline C	Robinson		PhD	Hospital Moinhos de Vento	Porto Alegre, Rio Grande do Sul, Brazil		Steering Committee	
Adriane I	Rohden		PhD	Hospital Moinhos de Vento	Porto Alegre, Rio Grande do Sul, Brazil		Steering Committee	
Bruna P	Gimenes		BsC	Hospital Moinhos de Vento	Porto Alegre, Rio Grande do Sul, Brazil		Steering Committee	
Cátia M	Guterres		MSc	Hospital Moinhos de Vento	Porto Alegre, Rio Grande do Sul, Brazil		Steering Committee	
Itiana C	Madalena		MSc	Hospital Moinhos de Vento	Porto Alegre, Rio Grande do Sul, Brazil		Steering Committee	
Luiza V	Andrighetto		PhD	Hospital Moinhos de Vento	Porto Alegre, Rio Grande do Sul, Brazil		Steering Committee	
Natalia E	Giordani		PhD	Hospital Moinhos de Vento	Porto Alegre, Rio Grande do Sul, Brazil		Steering Committee	
Sabrina S	da Silva		BsC	Hospital Moinhos de Vento	Porto Alegre, Rio Grande do Sul, Brazil		Steering Committee	
Daiana B	da Silva		MSc	Hospital Moinhos de Vento	Porto Alegre, Rio Grande do Sul, Brazil		Steering Committee	
Daniel	Sganzerla		MSc	Hospital Moinhos de Vento	Porto Alegre, Rio Grande do Sul, Brazil		Steering Committee	
Maicon	Falavigna		PhD	Hospital Moinhos de Vento	Porto Alegre, Rio Grande do Sul, Brazil		Steering Committee	
Regis G	Rosa		PhD	Hospital Moinhos de Vento	Porto Alegre, Rio Grande do Sul, Brazil		Steering Committee	
Alexandre B	Cavalcanti		PhD	HCor Research Institute	São Paulo, São Paulo, Brazil		Steering Committee	
Cassiano	Teixeira		PhD	Hospital de Clínicas de Porto Alegre	Porto Alegre, Rio Grande do Sul, Brazil		Steering Committee	
Cristiano A	Franke		MD	Hospital de Pronto de Socorro	Porto Alegre, Rio Grande do Sul, Brazil		Steering Committee	
Flávia R	Machado		PhD	Universidade Federal de São Paulo	São Paulo, São Paulo, Brazil		Steering Committee	
Joel	de Andrade		PhD	Central Estadual de Transplantes de	Florianópolis, Santa Catarina, Brazil		Steering Committee	
Luciano CP	de Azevedo		PhD	Hospital Israelita Alber Einstein	São Paulo, São Paulo, Brazil		Steering Committee	

Supplemental Online Content: Nonauthor Collaborators
*First name, last name, and suffix (if applicable) are required and will appear in PubMed.

***First Name and Middle Initial(s)**	***Last Name**	***Suffix (eg, Jr, III)**	Academic Degrees	Institution	Location (city, state/province, country)	Role or Contribution, eg, chair, principal investigator	Group (if more than 1 Group listed in the byline) and/or Subgroup (eg, Steering Committee)	
Luciano S	Hammes		PhD	Hospital Moinhos de Vento	Porto Alegre, Rio Grande do Sul, Brazil		Steering Committee	
Maureen O	Meade		PhD	McMaster University	Hamilton, Ontario, Canada		Steering Committee	
