# Supplementary material for: Evidence-Based Checklist to Delay Cardiac Arrest in Brain-Dead Potential Organ Donors: The DONORS Cluster Randomized Clinical Trial
Source: JAMA Netw Open. 2023 Dec 14;6(12):e2346901. doi: 10.1001/jamanetworkopen.2023.46901 (PMC10722341; doi:10.1001/jamanetworkopen.2023.46901)
Supplement: Supplement 4. — Data Sharing Statement [file jamanetwopen-e2346901-s004.pdf]

Data Sharing Statement

Westphal. Evidence-Based Checklist to Delay Cardiac Arrest in Brain-Dead Potential Organ Donors. *JAMA Netw Open*. Published December 14, 2023.
doi:10.1001/jamanetworkopen.2023.46901

Data
Data available: Yes
Data types: Deidentified participant data
How to access data: glauco.ww@gmail.com
When available: With publication

Supporting Documents
Document types: Statistical/analytic code
How to access documents: with the mannuscript as on line supplement
When available: With publication

Additional Information
Who can access the data: anyone requesting the data
Types of analyses: for a specified purpose
Mechanisms of data availability: with a signed data access agreement
